# Synergistic Catalytic Sites in High‐Entropy Metal Hydroxide Organic Framework for Oxygen Evolution Reaction

**DOI:** 10.1002/adma.202408114

**Published:** 2024-11-14

**Authors:** Arkendu Roy, Sourabh Kumar, Ana Guilherme Buzanich, Carsten Prinz, Emilia Götz, Anika Retzmann, Tilmann Hickel, Biswajit Bhattacharya, Franziska Emmerling

**Affiliations:** ^1^ Federal Institute of Materials Research and Testing (BAM) Richard‐Willstätter‐Str 11 12489 Berlin Germany; ^2^ Humboldt‐University Rudower Ch 25 12489 Berlin Germany; ^3^ Rigaku Europe SE Hugenottenallee 167 63263 Neu‐Isenburg Germany

**Keywords:** electrocatalysis, high‐entropy metal organic framework, in situ/operando XAS studies, oxygen evolution reaction and mechanism, water oxidation

## Abstract

The integration of multiple elements in a high‐entropy state is crucial in the design of high‐performance, durable electrocatalysts. High‐entropy metal hydroxide organic frameworks (HE‐MHOFs) are synthesized under mild solvothermal conditions. This novel crystalline metal–organic framework (MOF) features a random, homogeneous distribution of cations within high‐entropy hydroxide layers. HE‐MHOF exhibits excellent electrocatalytic performance for the oxygen evolution reaction (OER), reaching a current density of 100 mA cm^−2^ at ≈1.64 V_RHE_, and demonstrates remarkable durability, maintaining a current density of 10 mA cm^−2^ for over 100 h. Notably, HE‐MHOF outperforms precious metal‐based electrocatalysts despite containing only ≈60% OER active metals. Ab initio calculations and operando X‐ray absorption spectroscopy (XAS) demonstrate that the high‐entropy catalyst contains active sites that facilitate a multifaceted OER mechanism. This study highlights the benefits of high‐entropy MOFs in developing noble metal‐free electrocatalysts, reducing reliance on precious metals, lowering metal loading (especially for Ni, Co, and Mn), and ultimately reducing costs for sustainable water electrolysis technologies.

## Graphical abstract

A high‐entropy metal hydroxide organic framework (HE‐MHOF) is synthesized, combining five transition metals in a single‐phase crystalline structure. The material exhibits superior electrocatalytic performance for the oxygen evolution reaction, rivaling precious metal‐based catalysts. Operando X‐ray absorption spectroscopy and ab initio calculations reveal synergistic catalytic sites and the underlying reaction mechanism, demonstrating the potential of high‐entropy materials for sustainable energy technologies.

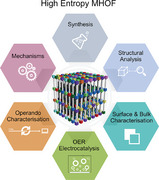

## 1 Introduction

Our dependence on fossil fuels, despite their detrimental impact on the environment, remains a pressing issue.^[^ ^1^, ^2^ ^]^ Therefore, it is crucial to develop alternative and sustainable energy technologies, such as water electrolysis for hydrogen fuel production. In this regard, the development of cost‐effective, noble metal‐free catalysts for water splitting is essential. Furthermore, meeting the pressing demand for catalyst stability poses a considerable obstacle to the progression of water electrolysis technology.^[^ ^3^ ^]^ High‐entropy materials (HEMs) have recently emerged as promising candidates for electrocatalysis.^[^ ^4^ ^]^ These materials have a complex composition, typically containing five or more equivalent elements and exist in a homogeneously mixed solid solution state while enhancing their thermodynamic stability. Their unique microstructure allows multiple properties to be optimized for their renowned “cocktail effect”.^[^ ^5^, ^6^ ^]^ In addition, the configurational entropy associated with the mixing of elements plays a key role in the synthesis of HEMs, counteracting the natural tendency for phase separation based on enthalpy preferences.^[^ ^7^ ^]^ This random mixing of elements leads to unique physical properties, for instance, improved specific strength,^[^ ^8^ ^]^ high‐temperature stability,^[^ ^9^ ^]^ corrosion resistance,^[^ ^10^ ^]^ ionic conductivities,^[^ ^11^, ^12^ ^]^ capacitive charge storage,^[^ ^13^ ^]^ ferroelectricity,^[^ ^14^ ^]^ and catalytic activity,^[^ ^4^, ^15^ ^]^ etc. These properties make HEMs invaluable across diverse industries, including aerospace, automotive, and energy sectors.^[^ ^16^ ^]^

Therefore, the need to achieve an optimal balance of properties also in electrocatalysts drives the current focus on HEMs for catalyst research. The reactivity of adsorbed species and the stability of intermediates are two significant parameters governing chemical reactions. In general, these two parameters work together to lower the activation barrier. Therefore, modifying the local structure and chemical environment of surface‐active sites in HEMs, ultimately accelerates the overall reaction on the high‐entropy surface.^[^ ^4^, ^17^ ^]^ However, the scarcity of operando characterization for HEMs hampers a comprehensive understanding of their mechanisms under working conditions. In particular, the reaction mechanism on high‐ entropy catalyst surfaces needs to be better understood before such materials can be selectively designed for targeted applications.

**Figure 1:**
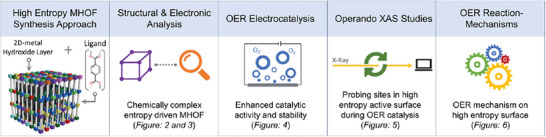
Schematic illustration depicting the high‐entropy metal hydroxide organic framework (HE‐MHOF).

In the water‐oxidation process, limitations, such as poor catalyst stability and a relatively higher thermodynamic activation barrier with sluggish reaction kinetics for the OER at the anode, hinder the overall water‐splitting reaction and thus affect the rate of hydrogen generation at the cathode.^[^ ^18^, ^19^ ^]^ These limitations call for developing highly active, cost‐effective, and durable electrocatalysts capable of effectively driving OER. In this context, high‐entropy layered hydroxides have attracted considerable interest in the field of electrocatalysis including OER due to the above‐mentioned advantages.^[^ ^20^, ^21^ ^]^ On the other hand, metal‐organic frameworks (MOFs) represent an important class of functional materials due to their structural diversification achieved by combining different metal centers and organic linkers.^[^ ^22^ ^]^ This unique flexibility creates an abundance of voids, making MOFs invaluable for various applications such as gas storage and separation,^[^ ^23^ ^]^ energy storage,^[^ ^24^ ^]^ catalysis,^[^ ^25^ ^]^ and many more. MOFs have emerged as a promising candidate for green hydrogen production via water electrolysis, due to their incredibly large surface area and porosity with a lower amount of metal loading compared to conventional materials.^[^ ^26^, ^27^ ^]^ Hydroxide‐based MOFs, referred to as metal hydroxide organic framework (MHOF), consisting of organic ligands with stronger π − π stacking energy have shown outstanding stability and promising catalytic activity for OER.^[^ ^28^, ^29^, ^30^ ^]^ Similarly to high‐entropy alloys (HEAs),^[^ ^7^ ^]^ oxides (HEOs)^[^ ^31^ ^]^ and ceramics (HECs),^[^ ^32^ ^]^ MOFs are anticipated to enter the domain of HEMs, with only a few recent examples demonstrating an embrace of the high‐entropy concept.^[^ ^12^, ^33^ ^]^ Thus, combining the design principles of high‐entropy layered hydroxides and MOFs, we have constructed a high‐entropy metal hydroxide organic framework (HE‐MHOF) (**Figure** **1**). This framework exhibits compositional diversity, enabling continuous and extended tuning of the catalyst for more efficient and durable OER activities. We investigated electronic and structural change of the high‐entropy surface during the electrocatalytic OER process using operando XAS.^[^ ^34^, ^35^, ^36^, ^37^ ^]^ These investigations are complemented by ab initio calculations, which facilitate a comprehensive understanding of the OER mechanism, elucidate the nature of synergistic catalytic sites and reaction pathways at the atomic level.

## 2 Results and Discussion

### 2.1 Synthesis, Structural, and Compositional Characterizations of HE‐MHOF

**Figure 2:**
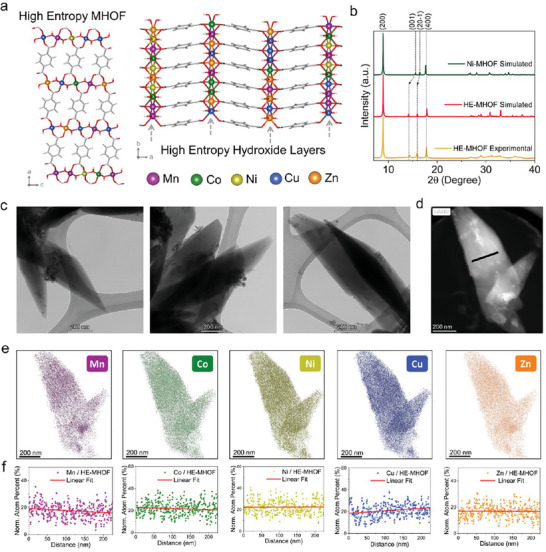
Structural and compositional analysis of HE‐MHOF. a) Perspective view of 3D HE‐MHOF structure and illustrating 2D layers of the high‐entropy metal hydroxide connected via organic linker‐terephthalic acid. b) Structural characterization by powder X‐ray diffraction of HE‐MHOF along with simulated patterns of high‐entropy MHOF and monometallic Ni‐MHOF, where 2θ is the angle between the transmitted and diffracted X‐ray beam. c) HR‐TEM Images of HE‐MHOF crystallites. d) HAADF image of HE‐MHOF single crystallite. e) Scanning Transmission Electron Microscopy (STEM) based elemental mapping of five different metals (Mn, Ni, Co, Cu, Zn) in a single crystallite of HE‐MHOF. f) Showing the distribution and atomic percentage of the five different metals along the black line drawn on the crystallite.

A crystalline high‐entropy hydroxide‐based MOF, designated as HE‐MHOF, was synthesized by reacting five different transition metal mixed hydroxides (Mn, Co, Ni, Cu, and Zn) with terephthalic acid as an organic linker under hydrothermal conditions for 48 h at 120 °C. The HE‐MHOF is isostructural to the monometallic Ni‐based MOF,^[^ ^38^ ^]^ but exhibits significant lattice distortion in the unit cell, demonstrating the compositional diversity achieved through the equimolar incorporation of five different metals (anticipated composition – Mn_0.2_Ni_0.2_Co_0.2_Cu_0.2_Zn_0.2_C_4_H_3_O_3_) in the high‐entropy approach. To elucidate the deconvoluted structural pattern, the special quasi‐random structure (SQS) approach^[^ ^39^ ^]^ was employed to simulate the chemical randomness in the HE‐MHOF (Figure S34, Supporting Information), using a 2 × 1 × 1 unit cell with 292 atoms (including 20 metal ions). The simulated surface of HE‐MHOF contains the 2D high‐entropy hydroxide layers, in which hydroxide (OH^−^) anions coordinate with three different metal centers and the metal centers attain a distorted octahedral geometry with six neighboring oxygen atoms from hydroxide anions (*ƞ* ^3^‐tridentate binding) and carboxylate groups (syn‐syn monodentate and bidentate bridging). These distorted‐octahedral metal centers are further connected in an edge‐to‐corner fashion in the (200) crystallographic plane separated by terephthalic acid molecules (Figure S1, Supporting Information). **Figure** **2**a illustrates the structure of HE‐MHOF, in which five equimolar metal cations form the high‐entropy hydroxide layers within a single‐phase MOF structure. These layers are uniformly and randomly mixed.

As anticipated, the simulated powder X‐ray diffraction pattern of HE‐MHOF similar to the one of Ni‐MHOF, e.g., the 1st and the 4th reflection are at a similar position confirming the similar interlayer spacing between hydroxide layers of HE‐ and Ni‐MHOF, corresponding to the (200) and (400) planes. In contrast, the 2nd and 3rd peaks are shifted in the high‐entropy‐simulated model due to the possible distortion caused by the incorporation of several metals in the hydroxide layers, resulting in a super cell (Figure 2b). Pawley refinement was performed on XRD data to refine the cell parameters of HE‐MHOF (Figure S3; Table S1, Supporting Information). The XRD data were indexed in the triclinic space group *P‐1*. Further, the previously mentioned simulated structure of the HE‐MHOF was used as the basis of the structural model for the Pawley refinement (Figure S34, Supporting Information). The average lattice parameters of the HE‐MHOF were determined to be a = 20.082(±1) Å, b = 3.164(±1) Å, c = 6.371(±1) Å, α = 90.50(±1), β = 95.46(±1), γ = 88.96(±1). Whereas the cell parameters of a similar Ni‐based MOF were reported as a = 19.8413(±4) Å, b = 3.31813(±6) Å, c = 6.26826(±13) Å, α = 90.00(±1), β = 96.5500(±11), γ = 90.00. (no. 985 792, Cambridge Crystallographic Data Center).^[^ ^38^ ^]^ To validate the single crystallite nature of HE‐MHOF, we performed 3D electron diffraction and calculated the lattice parameters (Table S10, Supporting Information), which were further compared with Ni/Co‐MHOF (Figures S37 and S38, Supporting Information). The recorded diffraction patterns of HE‐MOHF showed a resolution of up to 0.6 Å, confirming a single crystallite formation of HE‐MHOF (Figure S37, Supporting Information). The lattice parameters of HE‐MHOF closely resemble Ni/Co‐MHOF, with some deviations suggesting structural distortion due to the incorporation of five different metal ions in the crystal lattice.

Thermogravimetric analysis (TGA) revealed that the HE‐MHOF exhibited superior thermal stability in comparison to the monometallic Ni‐MHOF. The Ni‐MHOF experienced rapid weight loss and framework destruction below 250 °C, while the HE‐MHOF only showed a 5% weight loss up to 330 °C (Figure S4, Supporting Information). Thus, the high‐entropy approach in MHOF ensures improved thermal stability over a wider temperature range, as with other types of HEMs. The initial weight loss of ≈5% of HE‐MHOF can be attributed to removing water from the surface metal sites. This was substantiated by dynamic vapor sorption (DVS) measurements, which showed that HE‐MHOF experienced a 5% relative weight increase after exposure to 80% humidity (Figure S5, Supporting Information).

The crystallite morphologies and elemental compositions of the HE‐MHOF were analyzed using transmission electron microscopy (TEM) and energy dispersive spectroscopy (EDS). TEM images reveal the single crystalline nature of HE‐MHOF, while EDS measurements quantify metals in different crystallites (Figure 2c; Figure S6, Supporting Information). Our EDS results confirm the presence of five metals in nearly equimolar ratios in the chosen crystallites (Figure S7, Supporting Information). Elemental mapping further confirmed their distribution using high‐angle annular dark‐field scanning transmission electron microscopy (HAADF‐STEM), demonstrating a homogeneous distribution across a single crystallite (Figure 2d,e; Figure S8, Supporting Information). The relative quantification of Mn, Co, Ni, Cu, and Zn in the selected area (black line in Figure 2d), confirms the high‐entropy state of randomly mixed metals in hydroxide layer of HE‐MHOF. The near‐equal presence of all five metals, each constituting roughly ≈20% of the total metal centers within the selected area, reinforces this conclusion (Figure 2f). These results were corroborated by another quantitative measurement of the metals’ compositions in a bulk HE‐MHOF sample by inductively coupled plasma mass spectrometry (ICP‐MS) and revealed that HE‐MHOF contains metal percentages in a typical sample of Mn − 17% ± 3%, Co  −22%  ±  3%, Ni  − 22%  ±  3%, Cu  −22%  ± 3%, and Zn  − 17%  ± 3% (Table S9, Supporting Information). The observed quantities of metals from ICP‐MS analysis show near equimolar presence of metals in the bulk sample, as the quantified values overlap within the uncertainty. EDS and ICP‐MS analyses show that the HE‐MHOF has a vast compositional space resulting from the combination of multiple elements. The random mixing of elements may modify the local structure and chemical environment around the metal centers, possibly making surface active sites different in high‐entropy catalysts over conventional catalysts. Considering the random distribution, we simulated eight different surfaces by varying surface metal coordination fractions and compared their relative associated energies (Figure S34, Supporting Information). An apparent local structural distortion is observed for all the structures irrespective of their energetics, confirming the modification of the local structure. These distortions arise mainly due to the different stabilization of the M^2^⁺ oxidation state. A few significant factors contributing to this distortion are the tendency of metals to stabilize higher hydroxides and the Jahn–Teller effect because of the formal oxidation state (+2). However, these distortions are more pronounced at the surface, where atoms may have a lower coordination number and exhibit unsaturation than the bulk material. Such modifications are also seen in bulk, a direct consequence of the metal ion's *d*‐orbitals interacting with the local ligand field.

### 2.2 Electronic and Local Structure Characterizations of HE‐MHOF

**Figure 3:**
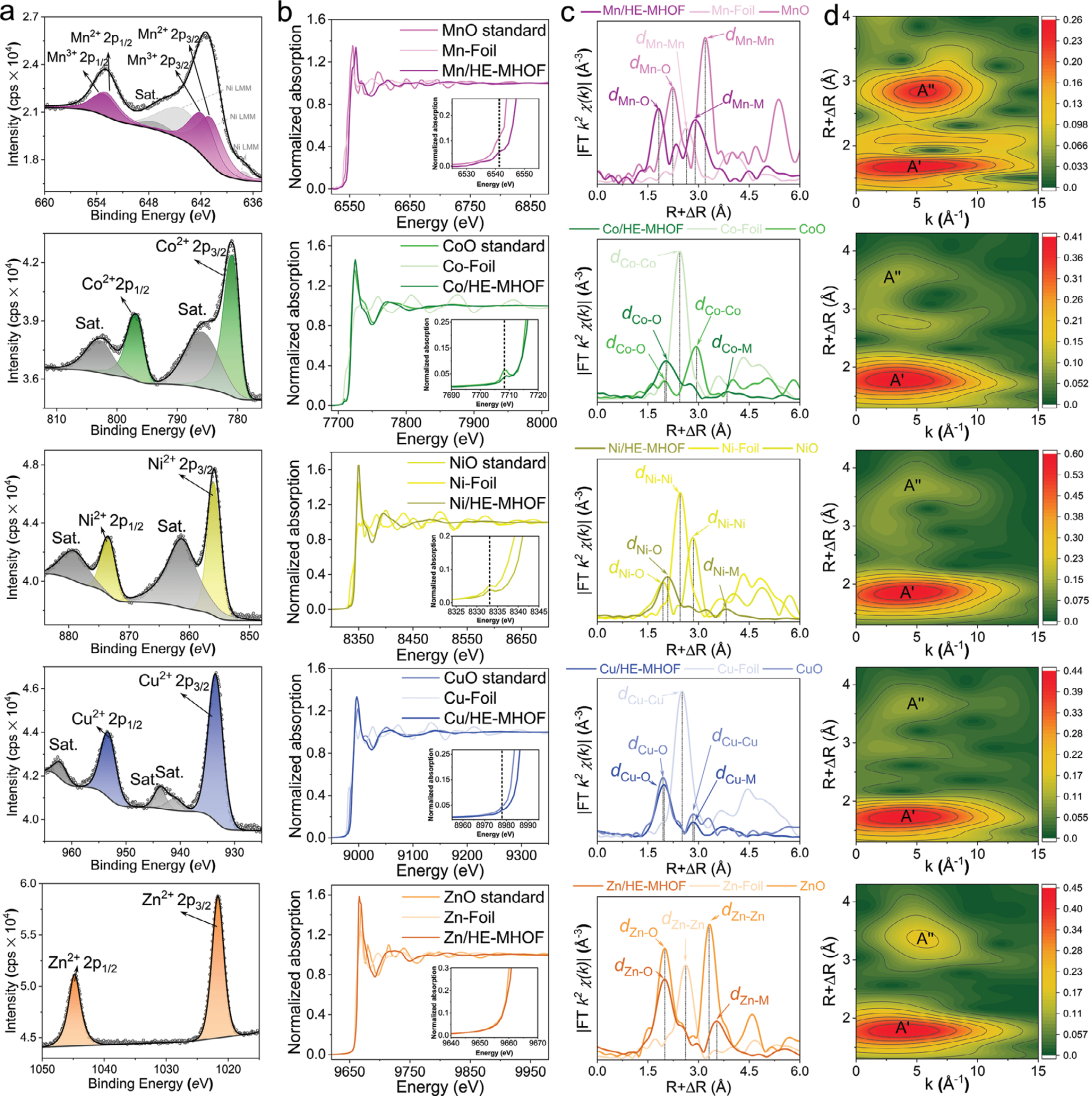
Electronic states and local structures of HE‐MHOF. a) X‐ray photoelectron Spectroscopy (XPS) spectra confirm the oxidation states of all five metals comprising HE‐MHOF. b) X‐ray absorption near edge structure (XANES) spectra features electronic transitions from the atomic K‐edge of all five transition metals (Mn, Co, Ni, Cu, and Zn) in HE‐MHOF, and the subplots represent the pre‐peak region of XANES spectra. c) Fourier‐transformed k^2^‐weighted extended X‐ray absorption fine structure (EXAFS) with phase shift depicting local structural environment surrounding each constituent metal with respective reference materials. d) Wavelet transformation of EXAFS spectra of each metal in HE‐MHOF. The intensity color scale depicts the magnitude of the wavelet transform (a.u.) ***Δ*** R denotes the phase shift.

The electronic states of the constituent metals on the surface were probed using X‐ray Photoelectron Spectroscopy (XPS). The analysis of survey XPS spectrum confirms the presence of all five metals (Figure S9, Supporting Information). A detailed XPS analysis of each metal element is given in **Figure** **3**a. The deconvolution of the Mn high‐resolution spectra confirms the presence of +2, and +3 oxidation states on the surface of HE‐MHOF. During the mixed hydroxide precipitation and MHOF formation steps, the oxidation of Mn^II^ to Mn^III^ may be caused by the dissolved oxygen in the solution.^[^ ^40^ ^]^ The Mn 2*p* spectrum displays the peak of Mn^II^ 2p_3/2_ at 640.7 *e*V, while for Mn^III^ 2*p*_3/2_ at 641.8 *e*V. The high‐resolution Co 2*p* spectrum shows peak at 780.7 *e*V for 2p_3/2_. The deconvolution of the Ni 2*p* spectrum shows peak at 855.8 eV for 2*p*_3/2_. Further, the Cu and Zn 2*p* spectrum shows peaks at 933.5 *e*V, and at 1021.1 *e*V for 2*p*_3/2,_ respectively (Figure 3a). The deconvolution of high resolution spectra confirms the presence of only +2 oxidation states for Co, Ni, Cu, Zn in HE‐MHOF.^[^ ^41^, ^42^, ^43^, ^44^ ^]^

XAS, which includes both X‐ray absorption near‐edge structure (XANES) and extended X‐ray absorption fine structure (EXAFS), was conducted to understand the local structure of the metals and their average electronic states in the bulk phase of HE‐MHOF.^[^ ^45^ ^]^ XANES data of Mn K‐edge of HE‐MHOF shows a higher energy absorption edge when compared to Mn K‐edge of MnO, which confirms the presence of Mn(III) in the HE‐MHOF (Figure 3b). As shown in Figure 3c, EXAFS spectra of the Mn K‐edge oscillation indicate the influence of the local environment of the Mn atoms in the HE‐MHOF. The first predominant peak at 1.8 Å is assigned to the Mn─O bond and the third peak at 2.9 Å to the Mn‐M _(neighboring)_ distance. The second peak at 2.3 Å is tentatively assigned to Mn^III^‐OH, which can form between surface/edge of Mn ions.^[^ ^40^, ^46^, ^47^ ^]^ The comparison of XANES spectrum between the K‐edge absorption energy of all other transition‐metals with their corresponding divalent oxides (such as CoO, NiO, CuO, ZnO), confirms their +2 oxidation state (Figure 3b; Table S2, Supporting Information). Thus, similar oxidation states were determined for the bulk phase HE‐MHOF sample as in surface‐sensitive XPS experiments.

The local structural environments of the other metals in HE‐MHOF were investigated from EXAFS data. The first predominant peak of Co atoms is located at an average distance of 2.0 Å, and which is assigned to average Co─O bond distance. In the case of Ni atoms, the average distance of the first scatterer (Ni─O average bond distance) from the excited atom is 2.1 Å, for Cu atoms this distance is 2.0 Å, and for Zn atoms it is 2.0 Å (Figure 3c). The EXAFS spectra of each metal present in the HE‐MHOF material are provided in the supporting material for the purpose of extracting quantitative information about the coordination environment of the absorbing metals, and to demonstrate the resemblance of the local structure in proximity to each metal with the simulated high‐entropy structure. (Figures S16–S25 and Tables S3–S7, Supporting Information). In addition, a wavelet transformation (WT) of the EXAFS spectra is provided as a convenient way to visualize the neighbor‐specific information and to compare the local atomic structure of all five metals in the HE‐MHOF at a glance (Figure 3d).^[^ ^35^ ^]^ The main feature in the WT‐EXAFS spectra is the neighboring O atom at a distance from 1.5 to 2.2 Å (feature A’). Scattering from neighboring metals, oxygen, and carbon atoms located outside of the first coordination sphere, contributes to feature A’’ at distance ranges between 2.2 and 4.0 Å. Thus, the overall structural and compositional analysis reveals the local‐structure surrounding all metal atoms dispersed homogeneously and collectively validates a compositionally diverse high‐entropy state in the HE‐MHOF.

### 2.3 Ab initio and Operando Studies of OER Active Sites

**Figure 4:**
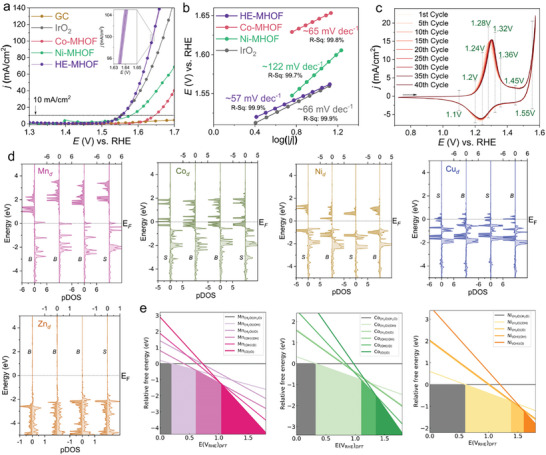
HE‐MHOF as catalyst: Electrochemical characterization and OER active sites. Electrochemical measurement of HE‐MHOF was performed in N_2_ saturated 1 mol L^−1^ NaOH solution using a glassy carbon rotating disk electrode under a fixed rotation of 1600 rpm. All the electrochemical measurements are represented at 85% solution resistance (R_u_) correction. a) Linear sweep voltammetry (LSV) measurement of HE‐MHOF was performed at 5 mV sec^−1^ under mentioned condition. The shaded band of the HE‐MHOF LSV trace correspond to the standard deviation of the counting statistics collected from three different HE‐MHOF synthesis batches. The horizontal line shows a current density of 10 mA cm^−2^.b) Tafel plots of HE‐MHOF, IrO_2_, and monometallic‐MHOFs c) Cyclic voltammetry (CV) measurement of HE‐MHOF was performed at 25 mV sec^−1^ scan rate, and under above mentioned conditions. d) Projected density of states (DOS) of Mn, Co, Ni, Cu, and Zn in HE‐MHOF. The Fermi level (E_F_) is denoted with dash lines and set to zero energy, and B or S refers to Bulk or Surface, respectively. e) Pourbaix diagram of Mn, Co, and Ni present on the surface of HE‐MHOF slab.

The OER activity of HE‐MHOF was evaluated in a conventional three‐electrode cell containing 1 mol L^−1^ NaOH solution at a low scan rate of 5 mV sec^−1^. All electrodes for the OER tests were prepared by depositing a homogeneous mixture of HE‐MHOF catalyst ink onto an electrochemically inert glassy carbon (GC) rotating disk electrode. This approach was chosen to investigate the inherent catalytic performance, rather than using other conductive metal substrates such as Ni or Cu foams.^[^ ^48^ ^]^ The HE‐MHOFs exhibit superior electrocatalytic oxygen evolution compared to both IrO_2_, a leading OER electrocatalyst, and monometallic (Ni, Co) MHOFs in a linear sweep voltammetry (LSV) measurement (**Figure** **4**a). Moreover, the presence of hetero‐metals and their random distribution in HE‐MHOF alter the electronic picture, synergistically enhancing its OER performance and making it novel compared to conventional OER catalysts. Similarly, in high‐entropy alloys, the “cocktail effect” is known to enhance the activity through the formation of unexpected synergies between different elements.^[^ ^49^, ^50^ ^]^ Insight into the mechanism of OER on the high‐entropy surface can be gained by evaluating the Tafel slope $b=(\frac{dE}{dlog|i|})$.^[^ ^51^, ^52^ ^]^ Here, the HE‐MHOF has a calculated Tafel slope of ≈57 mV dec^−1^ at the OER onset (Figure 4b). Later, the observed Tafel slope helps us to assign the probable rate limiting step and to elucidate the mechanistic sequence in OER.

To understand the catalytic activation and electrocatalytic performance of the HE‐MHOF surface, a HE‐MHOF surface is constructed with two adsorbed water molecules, stabilizing the octahedron around the metal center. Subsequently, an electronic structure calculation is performed for the best surface under consideration (Figure S34, Supporting Information). Our calculated average magnetic moment for Mn, Co, Ni, and Cu is 4.17 μ_*B*_, 2.62 μ_*B*_, 1.58 μ_*B*_ and 0.46 μ_*B*_, respectively, where Zn remains non‐magnetic, in the HE‐MHOF. Based on local magnetic moments, all the metal centers exhibit a high spin +2 oxidation state in the bulk and surface regime, supporting XANES and XPS measurements. However, a trivial case of Mn surface oxidation is possible due to oxygen in the atmosphere. This could be due to the stability of Mn^III/IV^ species forming after the oxidation of ^*^Mn(H_2_O)_2_ species present on the surface.^[^ ^53^ ^]^ To understand the chemical complexity that arises from the mixing of elements in HE‐MHOF, we plotted density of states for each individual atom. Figure 4d illustrates an atom‐resolved (and orbital‐resolved) density of states (DOS) for each transition metal in HE‐MHOF (surface and bulk). The strain induced by the metal‐ligand bond strength modifies the local structure around the metal center, ultimately modifying the electronic structure by removing the degeneracy of *d*‐orbitals among identical transition metals. In contrast to Zn, all the other metals have vacant *d*‐orbitals that are easily available for bonding in the +2 oxidation state. This makes the *d*‐orbitals in Zn more stable than those in other metals. However, despite a compositional disorder and modification of local electronic structure, we still observed a structured DOS for all transition metals.

Further, a cyclic voltammogram scan at the faradaic region was carried out, which clearly showed a partially reversible wave of redox active transition metals transforming from +2 to +3/+4 during pre‐redox, and vice versa upon reduction scan (Figure 4c). We anticipate that some redox active metal centers in alkaline solutions are more stabilized in a higher oxidation state, thereby hindering reversibility. The stability of the active sites on the surfaces of the HE‐MHOFs [^*^M‐(H_2_O)_2_] changes with applied potential at non‐equilibrium condition, which leads to a change in the oxidation state of the transition‐metals (M), present in their hydroxide form. To further understand the active species formation during pre‐redox process and to identify potential surface‐active sites in different regimes, DFT‐based surface Pourbaix diagrams were constructed for each OER active transition metal (Mn, Co, and Ni) using a slab model for the HE‐MHOF. According to the electronic structure calculations and surface Pourbaix diagrams (Figure 4e), the surface metals sites in the HE‐MHOF are covered by adsorbed H_2_O species (^*^H_2_O) at the DFT open circuit potential (from here onward referred as V_DFT,RHE_) suggesting most metals are present as +2 species. TGA and DVS measurement also support the presence of adsorbed water molecules (Figures S4 and S5, Supporting Information). Moreover, the deprotonation of ^*^Mn^II^‐(H_2_O)_2_ species into ^*^Mn^III^‐(OH)(H_2_O) species occurs at 0.21 V_DFT,RHE_ where further oxidation to ^*^Mn^IV^‐(OH)_2_ occurs around ≈0.62 V_DFT,RHE_ (Figure S35, Supporting Information). This suggests that the ^*^Mn‐(H_2_O)_2_ species can undergo oxidation to the Mn^IV^ species at a much lower potential compared to the OER onset point of 1.55 V_DFT,RHE_. This also supports the XPS and XANES measurements to validate the stability of Mn^III/IV^ species. The deprotonation of ^*^Co^II^‐(H_2_O)_2_ to ^*^Co^III^‐(OH)(H_2_O) occurs at ≈0.32 V_DFT,RHE_ in HE‐MHOF, representing a reduction of about ≈0.38 V_DFT,RHE_ compared to the Co atom in Co‐MHOF framework. On contrary, the deprotonation of ^*^Ni^II^‐(H_2_O)_2_ to ^*^Ni^III^‐(OH)(H_2_O) occurs at ≈0.62 V_DFT,RHE_ which is way lesser than Ni‐MHOF (at ≈1.22 V_DFT,RHE_) (Figure S35, Supporting Information). Besides, identifying potential surface‐active sites and to guide to in situ measurements, Pourbaix diagram also exhibits the stability of [^*^Ni(H_2_O)(O)] species formed after two step deprotonations, compared to the unary Ni‐MHOF which suggest the formation of [^*^Ni(OH)_2_] (Figure S36, Supporting Information). This shift in the redox potential for the Ni^III^ OER cycle was not observed for unary Ni‐MHOF. However, with increase in the applied potential, a tendency of forming Ni^IV^ species is always feasible, which could derive the kinetics differently compared to the Ni^III^. The following section further investigates the thermodynamic stability of these species using in situ/operando techniques.^[^ ^34^, ^35^ ^]^

**Figure 5:**
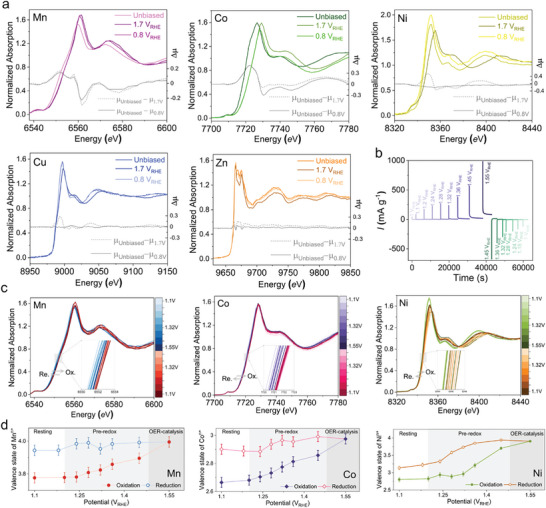
Operando XAS characterization of HE‐MHOF during OER. In situ/Operando X‐ray Absorption Spectroscopy (XAS) was employed to characterize the active sites of HE‐MHOF during oxygen evolution reaction (OER). a) K‐edge XANES spectra of all five transition metals (Mn, Co, Ni, Cu, Zn) in HE‐MHOF, showing K‐edge shifts during oxygen evolution reaction under applied potential. b,c) Deconvolution of the partially reversible cyclic voltammogram of HE‐MHOF via K‐edge XANES spectra of Mn, Co, Ni under different potentials. d) Change in Mn, Co, and Ni valance state as function of applied potential. (Valance state determination in Supplementary Method, Supporting Information for further details) Note that the OER current at the operando XAS cell electrode and rotating disk electrode is not same because of the different substrate and mass transfer limitations, nevertheless, the trend in the potential dependent current output is consistent. The determined average oxidation states are tentative, assuming that metal centers (e.g., Mn, Co, Ni) in the high‐entropy matrix exhibit similar energy shifts relative to oxidation states as observed in their respective reference materials. Further, the uncertainty is calculated by error propagation method for fitting error and standard‐foil measurement error from the beamline, but only on single oxidation‐reduction cycle (See Note S7, Supporting Information).

In order to reveal the nature of the active sites during OER, operando XAS was performed on the K‐edge absorptions of each of the constituent metals in HE‐MHOF, establishing the oxidation state changes during the OER from the evolution of the XANES spectra (Supplementary Methods, Supporting Information). The in situ cell was placed under constant potentials at 1.70 V_RHE_ for catalysing OER and at 0.80 V_RHE_ to understand the reversibility of the catalytic centers. **Figure** **5**a compares the potential‐dependent changes from unbiased state (at open circuit potential) by the difference of oxidized and reduced states of XANES spectra (Δμ) for all five different metals in HE‐ MHOF (Corresponding electrochemical measurements of the in situ cell is shown in Figure S28, Supporting Information). Interestingly, this comparison of XANES spectra unveil that Mn absorption edge had been shifted by ≈3.90 *e*V, changing oxidation states from Mn^II/III^ to Mn^IV^, but remains unchanged upon reduction (Figure 5a; Table S8, Supporting Information). In the case of Co atoms in HE‐MHOF, the K‐edge absorption was shifted 2.8 *e*V, may be connected to Co^II^ changing its oxidation state to Co^III^. During the reduction process, Co^III^ species was observed to be stabilized at the current state in 1 mol L^−1^ NaOH solution (Figure 5a; Table S8, Supporting Information). The spectra of the Ni atoms showed changes in the absorption edge energy of ≈2 *e*V, corresponding to an oxidation state change from Ni^II^ to Ni^III/IV^. At 0.8 V_RHE_, Ni had gone for 1 electron reduction followed by a change in K‐edge absorption energy value decreased by 2 *e*V, while stabilizing Ni^III^ in 1 mol L^−1^ NaOH (Figure 5a; Table S8, Supporting Information). However, we have not observed any K‐edge absorption energy shift upon oxidation or reduction for the remaining two metals such as Cu and Zn in HE‐MHOF (Figure 5a). Furthermore, to probe the pre‐redox region of the electrocatalysis, the K‐edge XAS spectra of Mn, Co, Ni were measured during a stepwise electrochemical cycling (Figure 5b). According to the spectral evolution, we can divide the oxidation scan into three stages: the resting stage from 1.10 V_RHE_ to 1.20 V_RHE_ (stage I), the pre‐redox stage from 1.20 V_RHE_ to 1.48 V_RHE_ (stage II), and the OER catalytic stage from 1.48 V_RHE_ to 1.55 V_RHE_ (stage III). These are evidenced by the electrocatalytic measurements showing negligible OER current up to the applied potential of 1.45 V (Figure 4c; Figure S29, Supporting Information). In stage I, the K‐edge XANES spectra of the Mn, Co, Ni at 1.1 V_RHE_ have a significant positive shift compared with K‐edge of the pristine sample, owing to the tendency of deprotonation of M^II^‐(H_2_O)_2_ to M^III^‐(OH)(H_2_O) in 1 mol L^−1^ NaOH solution from Pourbaix diagram (Figure 4e). With an applied potential larger than 1.20 V (stage II and III) the most obvious feature is that the metals’ K‐edges shift to higher energy (Figure 5c). The oxidation state of the Mn in our HE‐MHOF obtained to be varying up to +4 upon gradual increase of applied potential up to 1.55 V_RHE_, owing to stabilized Mn^IV^‐(O)(O) species from Mn Pourbaix diagram.^[^ ^54^ ^]^ At the Co K‐edge, the XANES spectra similarly shifted positively while changing oxidation state from +2 to +3 under catalytic potential of 1.55 V_RHE_. In accordance with the Co Pourbaix diagram, Co^III^‐(H_2_O)(OH) is confirmed as the active species in the pre‐redox state, and Co^III^ is identified as the reactant in the rate‐limiting step during the OER.^[^ ^55^, ^56^ ^]^ In contrast to the other two metals, Ni shows reversibility in K‐edge energy shift upon oxidation and reduction scan. The XANES spectra of Ni indicates the change of the oxidation state from +3 to +4 under OER potential of 1.55 V_RHE_, confirming that the ^*^Ni‐(H_2_O)(O) species (from Pourbaix diagram Figure 4e) stabilize during the OER process as a reactant state of the rate limiting step and show reversibility upon reduction at 1.1 V_RHE_ while stabilizing Ni^III^‐(HO)(H_2_O) (Figures 4, 5).^[^ ^57^, ^58^ ^]^

The structural evolution of active sites is also collaborated with the Fourier transform analysis of EXAFS (FT‐EXAFS) results (Figure S31, Supporting Information). In particular, the first M─O bond distance at ≈1.70 Å remains unchanged during OER catalysis at 1.55 V_RHE_ in comparison to the resting state at 1.1 V_RHE_ in 1 mol L^−1^ NaOH solution. This observation is consistent with the Pourbaix diagram of Mn, which suggests the formation of Mn‐(OH)(OH) and Mn(O)(O) at the resting and catalytic stages, respectively.^[^ ^59^ ^]^ The first M─O bond distance at ≈1.70 Å for Co and at ≈1.90 Å for Ni also remains unchanged, but peak intensity changes for Co and Ni at the catalytic stage (1.55 V_RHE_) in comparison to the resting state (1.1V_RHE_). This observation depicts the formation of new oxyhydroxides or oxide species like Co‐(OH)(O) or Ni‐(O) on surface during the OER catalysis^[^ ^47^, ^49^, ^50^, ^60^ ^]^ (Figure 4e). Additionally, the HE‐MHOF electrode used for operando studies was air‐dried and characterized by XRD post‐OER, confirming the retention of its pristine structure, as shown in Figure S32 (Supporting Information). Our operando measurements and ab initio simulations enable us to identify potential active species during pre‐redox and OER. This approach also helps us propose a plausible OER mechanism for the high‐entropy surface and confirm the stability of HE‐MHOF following OER catalysis.

### 2.4 Reaction‐Mechanism of OER on HE‐MHOF Surface

A high‐entropy structure offers a significant performance improvement compared to the monometallic MHOF, particularly in terms of onset potential and geometric current density. This presents an excellent opportunity to delve into the OER mechanism for each OER active metal site of HE‐MHOF. The onset overpotential (at 1 mA cm^−2^) for HE‐MHOF is ≈260 mV, comparable to the onset potential for IrO_2_ under similar conditions, whereas a current density of 100 mA cm^−2^ is achieved for the HE‐MHOF at higher overpotentials (≈0.41 V) and exhibits stability at 10 mA cm^−2^ geometric current density in 1 mol L^−1^ NaOH solution for more than 100 h at an overpotential of ≈0.3 V (Figure S27, Supporting Information). The HE‐MHOF offers comparable performance to IrO_2_ for the OER, thereby surpassing the limitations posed by the noble metal present in IrO_2_. On the other side, the overpotential at 10 mA cm^−2^ for monometallic Co‐MHOF, Ni‐MHOF are approximately ≈416 mV and ≈350 mV, which is way higher than the value for the HE‐MHOF (Figure 4a). To understand the kinetics of the OER, a Tafel slope for HE‐MHOF is calculated at the onset of OER, ≈57 mV dec^−1^. This value is somewhat comparable to those of IrO_2_ ≈66 mV dec^−1^ around and Co‐MHOF ≈65 mV dec^−1^. However, this value completely differs from Ni‐MHOF (≈122 mV dec^−1^) (Figure 4b). This observed ambiguity is attributed to the synergistic behavior resulting from the cooperativity of metals within the HE‐MHOF.

**Figure 6:**
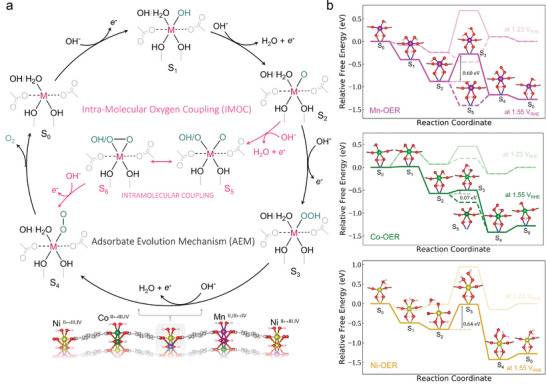
Reaction‐mechanism of OER on high‐entropy surface. a) Proposed adsorbate evolution mechanism (AEM) and intramolecular O─O coupling mechanism (IMOC) in HE‐MHOF, b) Free energy diagrams of the OER cycle for each OER active metal center (Mn, Co, and Ni) under consideration at required thermodynamic potential (1.23 V_RHE_) and potential considered for the calculation of the Tafel slope (1.55 V_RHE_). All possible states are represented by S_n_ symbols and the O─O coupling is represented by dashed lines in the free energy diagrams.

**Figure** **6** illustrates a free energy diagram based on Density functional theory (DFT) calculations that compares the OER energetics considering the adsorbate evolution mechanism (AEM) against the intramolecular O─O coupling (IMOC) pathway (Figure 6a). The mechanism for the adsorbate oxygen evolution reaction in MHOFs, involving the formation of ^*^OOH species, has already been reported.^[^ ^30^ ^]^ Previously, through our CV and LSV measurement, we observed that catalytic activation for OER active metals occurs at a much lower electrode potential for the HE‐MHOF. This is consistent with the Pourbaix diagram for the HE‐MHOF, where the presence of neighboring Mn or Co atoms lowers the pre‐redox potential of Ni. Our operando measurements also confirm the formation of M^III/IV^ hydroxides. From ab initio simulations, we observed that the HE‐MHOF surface is dominantly covered with the ^*^O (or ^*^OH) species at ≈1.55 V_DFT,RHE_. In accordance with the conventional AEM, the ^*^O─O coupling (peroxide formation; S_3_) is the rate‐determining step (RDS), leading to the formation of ^*^OOH [S_3_] species in the free energy diagram. For Mn and Ni, the barrier for the ^*^O─O coupling is found to be 0.60 and 0.64 *e*V, whereas Co has a much lower barrier of ≈0.07 *e*V (Figure 6b). On the contrary, the formation of ^*^Co‐(OH)_2_ [S_1_] necessitates a much higher potential, resulting in a flat surface in the free energy diagram (at 1.55 V_DFT,RHE_) compared to Ni‐S_1_ and Mn‐S_1_ species. This is directly related to the significant change in peak intensity observed in the FT‐EXAFS results for Co at 1.55 V_RHE_ (Figure S31, Supporting Information). Considering the ^*^OOH formations as an RDS, the deprotonation of ^*^OOH can occur through a chemical or electrochemical process, followed by the ^*^O_2_ desorption.

Here, it is noteworthy to assume that AEM only involves the formation of peroxide species as one of the intermediary steps in the OER, which is supported by our XANES and FT‐EXAFS measurements, clearly suggesting the formation of oxide as a reactive species (for Ni) during the OER in HE‐MHOF. In the context of Ni‐MHOF, the formation of double hydroxide species [Ni(OH)_2_] is favored over oxide species [Ni(H_2_O)(O)]. Subsequently, the ^*^O─^*^O coupling mechanism becomes a more viable pathway for the OER (Figures S35 and S36, Supporting Information). In previous reports, the attack of the intra‐nucleophilic hydroxyl group (as depicted in Figure 6) is reported as one of the pathways for intramolecular ^*^O─^*^O coupling within Co‐metal in Aza‐CMP.^[^ ^61^ ^]^ Due to the spatial constraints imposed by the terephthalic acid moiety and the nucleophilicity of hydroxyl ions, there arises a competition between intramolecular (resulting from the initial deprotonation of adsorbed water) and intermolecular hydroxyl (present in the solution) attack for oxygen–oxygen coupling. In a strongly alkaline solution, the deprotonation of the second hydroxyl (S_5_) becomes more favorable for Co and Mn with applied potential, resulting in a different pathway for the OER. Here, the intramolecular ^*^O─^*^O coupling is also possible for S_5_ species, as shown in Figure 6. This directly correlates with experimental observations, suggesting no significant change in the peak intensity, specifically for Mn. However, for Co, the initial formation of Co(OH_2_) necessitates a higher potential, causing a significant change in peak intensity (Figure S31, Supporting Information). The deprotonation of second 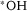 species requires much less potential compared to the formation of 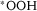 species. The intramolecular coupling between the oxide species favored by a hydroxyl attack resulting in the formation of M‐(O_2_)(OH) species [S_4_]. Later, the O_2_ desorption from metal site is found to be energy demanding step for Co rather than Mn. Several catalysts demonstrate OER with O_2_ desorption as one of the major factors in driving the kinetics of the reaction, regardless of the chosen mechanism.^[^ ^62^ ^]^ Ni and Co have shown ^*^O_2_ desorption as a rate‐limiting step with a binding energy of 0.10–0.15 *e*V, which could directly affect the kinetics concerning different potential regimes.

In correlating the observed kinetics, specifically the Tafel slope with the OER mechanism, we considered a few assumptions; a) all the OER active elements participates in driving the reaction, b) prior to the RDS, all reactions are in quasi‐equilibrium, proceeding rapidly in both forward and backward direction, and c) reactions occurring after the RDS only modifies the height of the current/overpotential curve not the shape of the current/overpotential curve. Now, the Tafel slope (*b*) can be related to the transfer coefficient by the expression; $b=\frac{\partial E}{\partial \log|i|}=\frac{2.303\mathrm{RT}}{\alpha F}$. [Tafel Analysis in Supplementary methods, Supporting Information] A theoretical model can be constructed to understand the observed kinetics (at 1.55 V_RHE_), considering the transfer coefficient (α  =  *n_p_* +  *n_r_* × β[  = 0.5 ]), which depends on the number of electrons transferred prior to the electrochemical reactions (*n_p_*) and at the RDS (*n_r_*) for multistep chemical reactions. A value of 0.5 and 1.0 for transfer coefficient is considered to correlate the observed kinetic in different potential regime (≈60 mV dec^−1^, at onset potential and ≈120 mV dec^−1^, at E > 1.7 V_RHE_). For Mn, considering the IMOC with ^*^O─^*^O coupling as OER mechanism, the formation of [Mn(O_2_)(OH)] requires hydroxyl attack step as electrochemical (*n_r_* = 1) reaction followed by the desorption of O_2_. As O_2_ desorption is always favored in the free energy diagram for Mn, a Tafel slope of ≈120 can by calculated for the Mn sites [α  =  0 + 1 ×  0.5]. Considering the Co as active OER site, with ^*^O─^*^O coupling as a favored mechanism, where the desorption of O_2_ can be considered as a non‐concentrated electron transfer process $\mathrm{Co}\left(O_{2}\right){\left(\mathrm{OH}\right)}^{-}\overset{R_{1}}{\to }\mathrm{Co}\left(O_{2}\right)\left(\mathrm{OH}\right)\overset{R_{2}}{\to }\mathrm{Co}\left(\mathrm{OH}\right)$. If *R_2_* considered as a RDS followed by 1*e* ^−^ transfer prior to the desorption, a transfer coefficient value of 1 can be assigned [α_*f*_ =  1 + 0 ×  0.5] resulting in a Tafel slope of ≈60. Similarly, for Ni as active OER sites, the formation of 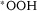 species is considered as RDS $\left[\mathrm{Ni}\left(H_{2}O\right)\left(O\right)\right]\overset{R_{1}}{\to }\mathrm{Ni}\left(H_{2}O\right)\left(\mathrm{OOH}\right)$. Followed by the deprotonation of Ni(H_2_O)(OOH) to Ni(H_2_O)(OO) and desorption of O_2_ as a rate limiting step like cobalt. If the deprotonation of 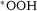 species occurs as a chemical step in alkaline conditions, followed by desorption of O_2_ and charge transfer as a non‐concentrated step, then a transfer coefficient value of 1 can be assigned [α  =  1 + 0 ×  0.5] resulting in a Tafel slope of ≈60. However, the synergistic behavior of transition metals in HE‐MHOF may lead to more complex intermediates and kinetics driven by intricate pathways during the OER. Future work integrating data from operando spectroscopy and ab initio calculations holds the potential to offer deeper mechanistic insights for HE‐MHOF, offering their functionalities beyond OER.

## 3 Conclusion

We present a strategic synthesis of a metal‐organic framework based on a high‐entropy (HE) hydroxide layer derived from a known Ni‐based metal hydroxide organic framework (Ni‐MHOF). Through structural and compositional analysis, we confirm the formation of a single crystallite of HE‐MHOF, where five different metals are uniformly distributed within the 2D high‐entropy hydroxide layer. The HE‐MHOF shows significant potential for the oxygen evolution reaction, achieving a current density of ≈100 mA cm^−2^ at a potential of ≈1.64 V_RHE_. This level of performance is comparable to precious metal‐based catalysts such as IrO_2_ (100 mA cm^−2^ at ≈1.67 V_RHE_). The HE‐MHOF maintains a geometric current density of 10 mA cm^−2^ beyond 100 h at an overpotential of ≈0.3 V, indicating the stability of the electrocatalyst. Through operando X‐ray absorption spectroscopy studies and Pourbaix plots, we observe the ability of the metals to reach higher valence states by oxidizing adsorbed water molecules, while also gaining insight into the various active oxygenated species present on the HE‐MHOF surface. Extended X‐ray absorption fine structure results and ab initio calculations reveal that the formation of a high‐entropy matrix induces local structural modifications around the metal center, which leads to the removal of *d*‐orbital degeneracy between identical metal atoms on the surface and in bulk, as observed in the density of states. These changes create a synergistic effect in the HE‐MHOF, enhancing its electrocatalytic activity. Our research suggests that ≈1.55 V_RHE_, Mn and Co engage in an intramolecular oxygen–oxygen coupling mechanism, whereas Ni in HE‐MHOF follows an adsorbate evolution mechanism, showing a difference compared to Ni in Ni‐MHOF due to a synergistic effect, which is supported by operando techniques and ab initio simulations. By continuing to explore HE‐MHOFs using ab initio methodologies and operando techniques, we could acquire deeper insights into the reaction kinetics between similar catalytic sites and competitive reaction mechanisms and how the local coordination environment affects catalytic properties in high entropy materials, paving the way for further optimization and scaling up chemical reactions controlled by the catalyst. This study highlights the significant potential of a high entropy matrix in MHOFs, leading to synergistic effects that pave the way for sustainable water electrolysis technologies and revolutionize the field of electrocatalysts in applications beyond water electrolysis.

## 4 Experimental Section

Detailed descriptions of all synthesis procedures, characterization methods, and computational methodologies are provided in the supporting information. In brief, the synthesis of a high‐entropy metal hydroxide organic framework (HE‐MHOF) containing five transition metals (Mn, Co, Ni, Cu, and Zn) is performed using a hydrothermal method. The material was extensively characterized using various techniques including powder X‐ray diffraction (PXRD), thermogravimetric analysis (TGA), transmission electron microscopy (TEM), energy‐dispersive X‐ray spectroscopy (EDS), X‐ray photoelectron spectroscopy (XPS), and X‐ray absorption spectroscopy (XAS), etc. Electrochemical measurements were performed using Biologic Potentiostat to evaluate the material's catalytic activity for the oxygen evolution reaction. In addition, Density functional theory (DFT) calculations were carried out using the Vienna Ab initio Simulation Package (VASP) to model the HE‐MHOF structure and investigate reaction mechanisms.

## Conflict of Interest

The authors declare no conflict of interest.

## Supporting information



Supporting Information

## Data Availability

The data that support the findings of this study are available from the corresponding author upon reasonable request.
