# Supplementary material for: Synergistic Catalytic Sites in High‐Entropy Metal Hydroxide Organic Framework for Oxygen Evolution Reaction
Source: Adv Mater. 2024 Nov 14;36(52):2408114. doi: 10.1002/adma.202408114 (PMC11681304; doi:10.1002/adma.202408114)
Supplement: Supplementary file 1 — Supporting Information [file ADMA-36-2408114-s001.pdf]

ADVANCED MATERIALS

Supporting Information

for *Adv. Mater.*, DOI 10.1002/adma.202408114

Synergistic Catalytic Sites in High-Entropy Metal Hydroxide Organic Framework for Oxygen Evolution Reaction

Arkendu Roy, Sourabh Kumar, Ana Guilherme Buzanich, Carsten Prinz, Emilia Götz, Anika Retzmann, Tilmann Hickel, Biswajit Bhattacharya* and Franziska Emmerling**

Supplementary Information

Synergistic Catalytic Sites in High-Entropy Metal Hydroxide Organic Framework for Oxygen Evolution Reaction

Arkendu Roy[1,2,4], Sourabh Kumar[1,4], Ana Guilherme Buzanich[1], Carsten Prinz[1], Emilia Götz[3], Anika Retzmann[1], Tilmann Hickel[1,5], Biswajit Bhattacharya[1,5], Franziska Emmerling[1,2,5]

1. Federal Institute of Materials Research and Testing (BAM), Richard-Willstätter-Str 11, 12489, Berlin, Germany

2. Humboldt-University, Rudower Ch 25, 12489, Berlin, Germany

3. Rigaku Europe SE, Hugenottenallee 167, 63263, Neu-Isenburg, Germany

4. These authors contributed equally

5. Corresponding authors

INDEX:

1.	Structural Illustration of HE-MHOF	page no. 3
2.	Powder X-ray Diffraction (PXRD)	page no. 4
3.	Thermogravimetric Analysis (TGA)	page no. 5
4.	DVS Measurement of HE-MHOF	page no. 6
5.	Transmission Electron Microscopy (TEM) Images of HE-MHOF	page no. 7
6.	Energy-Dispersive X-ray Spectroscopy (EDS) Data	page no. 8
7.	High-Angle Annular Dark-Field Scanning Transmission Electron Microscopy (HAADF-STEM) Image and Mapping	page no. 9
8.	X-ray Photoelectron Spectroscopy (XPS) Analysis	page no. 10-13
9.	X-ray Absorption Spectroscopy (XAS) Spectra: X-ray Absorption Near-Edge Structure (XANES) and Extended X-ray Absorption Fine Structure (EXAFS) of HE-MHOF	page no. 14-18
10.	Electrochemical Measurements	page no. 19-22
11.	Operando X-ray Absorption Spectroscopy (XAS) Spectra during OER electrocatalysis	page no. 23-25
12.	Surface Configurations of HE-MHOF	page no. 26
13.	*Ab-initio* Calculation on monometallic MHOFs	page no. 27-28
14.	Three-Dimensional Electron Diffraction (3D-ED)	Page no. 29-30
15.	Supplementary Methods	page no. 31-36
16.	Synthesis Procedure	page no. 37
17.	Supplementary Tables	page no. 38-46
18.	Supplementary Notes	Page no. 47-50
19.	References	page no. 51-52

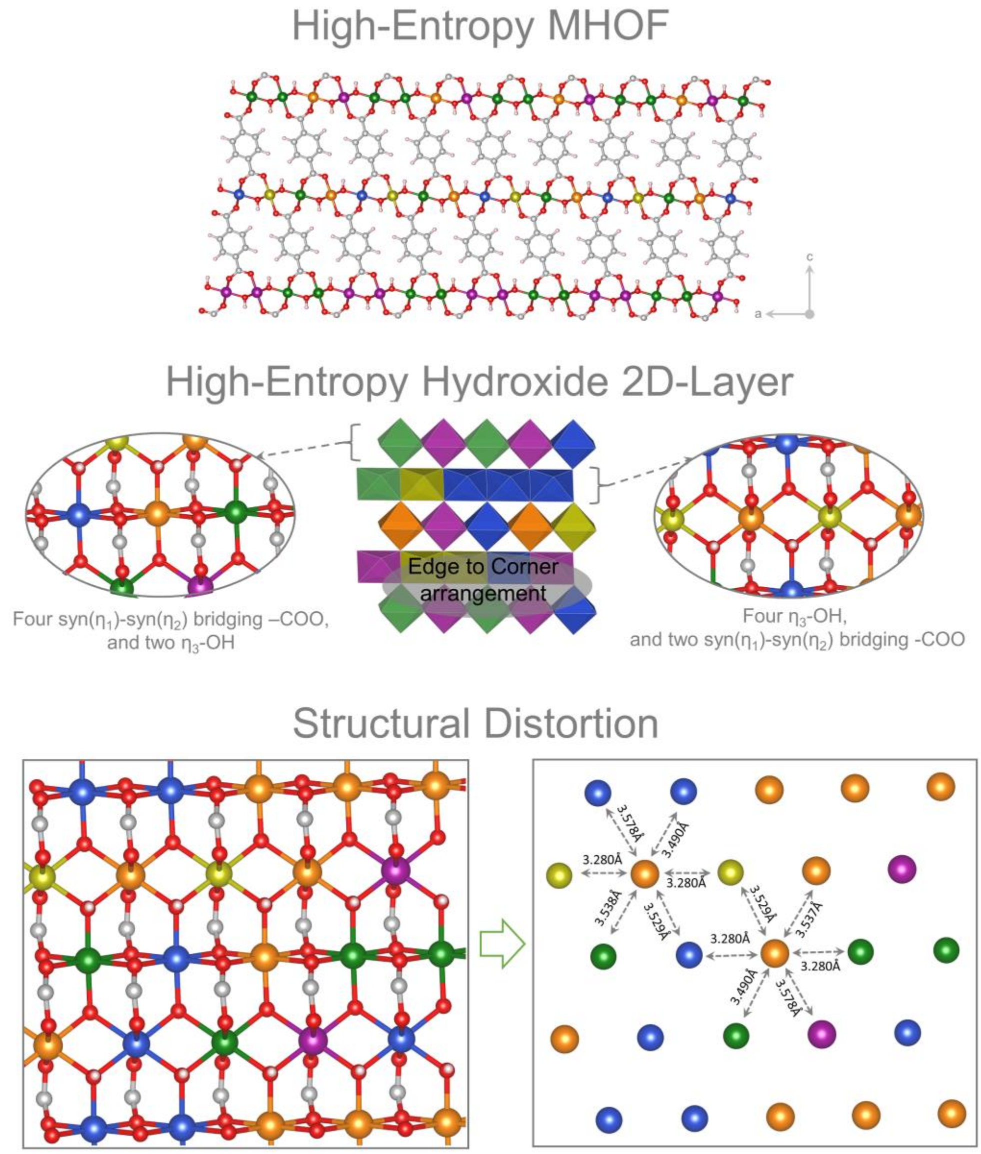

Figure S1. High-Entropy Metal hydroxide Framework (HE-MHOF) consists of five transition metals, such as Mn, Co, Ni, Cu, and Zn. The structure of HE-MHOF is viewed along the *b*-direction. In addition, the ‘edge to corner’ arrangement of metal octahedrons in a 2D high entropy hydroxide layer is shown. This 2D layer is exfoliated from a simulated HE-MHOF bulk structure (See computational methodologies) to show local lattice distortion (such as metal-metal distance) and structural randomness.

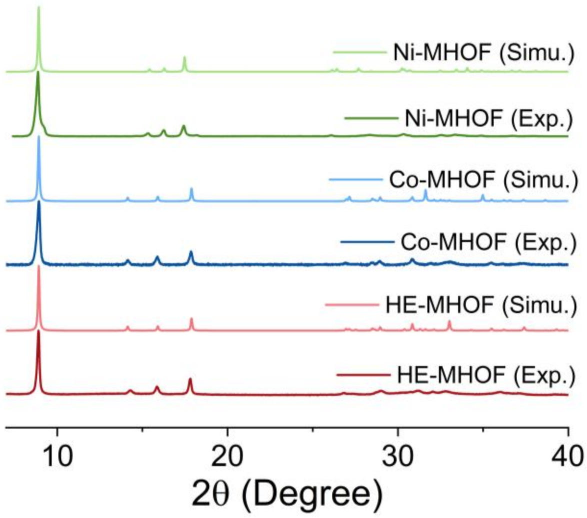

Figure S2. Powder X-ray diffraction (PXRD) patterns of high entropy metal hydroxide organic framework (HE-MHOF) and PXRD patterns of monometallic (Ni and Co), stacked with their respective simulated PXRD patterns.

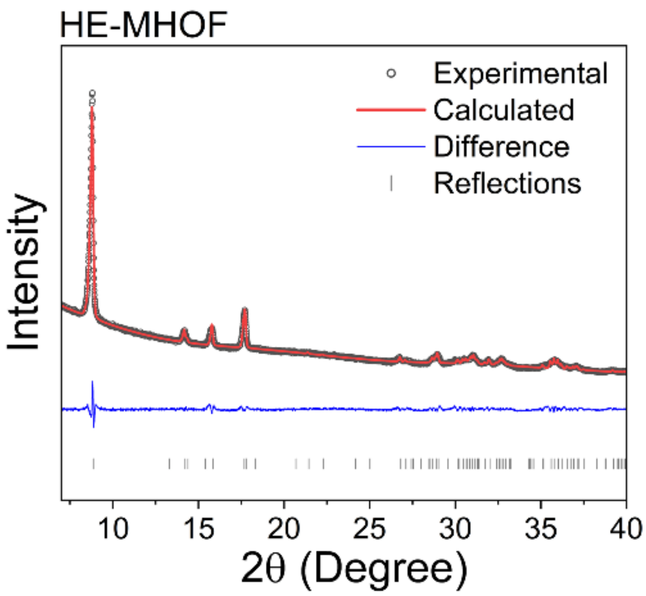

Figure S3. Pawley fit of PXRD patterns (λ = 1.5418 Å) for HE-MHOF, Red line: calculated pattern, black line: observed pattern, blue line: difference between calculated and observed patterns. The refined unit cells after the Pawley refinement are shown in the Table S1.

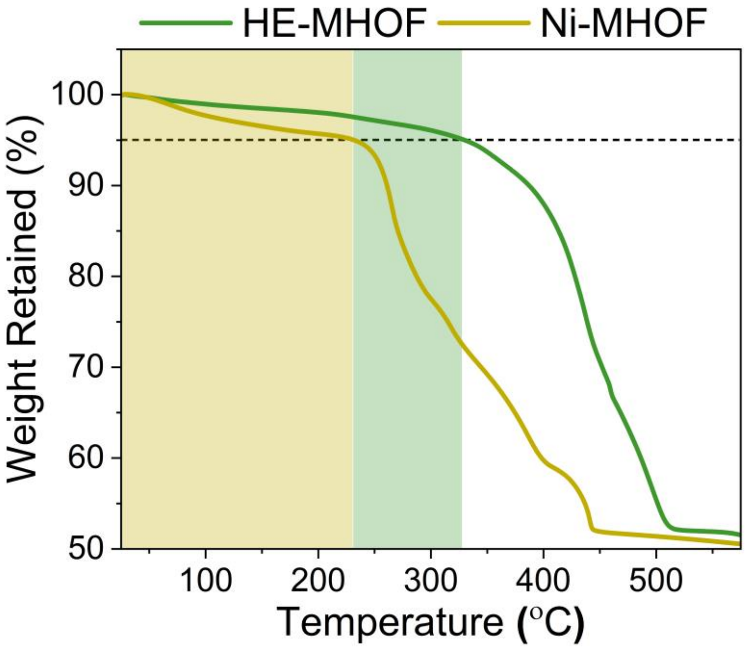

Figure S4. Thermogravimetric analysis (TGA) curves of the monometallic Ni-MHOF and HE-MHOF at pristine condition (heating rate 10ºC min^{-1} under N_2 flow). From the thermogravimetric analysis, it is observed that the HE-MHOF is stable at 330 ^oC within 5 % weight loss. Then, a gradual weight loss 52 % has been observed up 510 ^oC, substantiate decomposition of the framework structure, especially the organic part. In conclusion, HE-MHOF shows better thermal stability than monometallic Ni-MHOF.

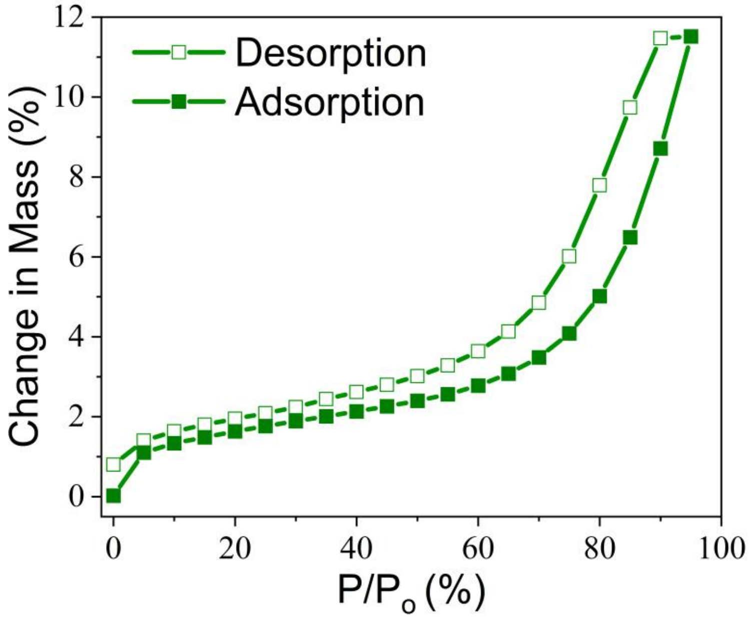

Figure S5. Dynamic vapor sorption (DVS) isotherms of HE-MHOF. DVS measurement was performed to substantiate the water uptake, which can be co-related with the initial weight loss in the TGA analysis of HE-MHOF.

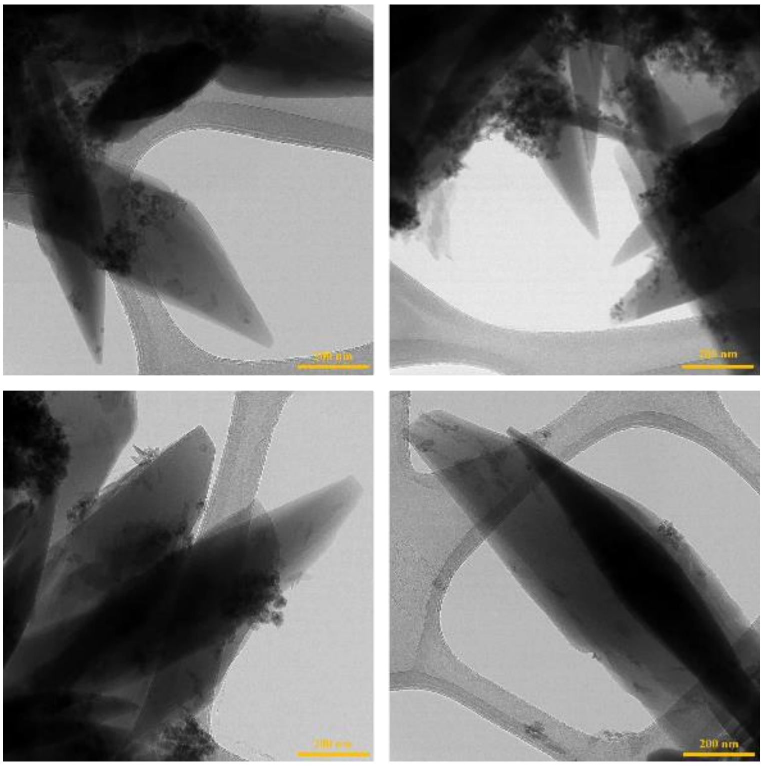

Figure S6. TEM Images of HE-MHOF crystallites. TEM images of HE-MHOF substantiate the single crystalline nature of entropy stabilized MOF and reveal the morphology of the crystallites.

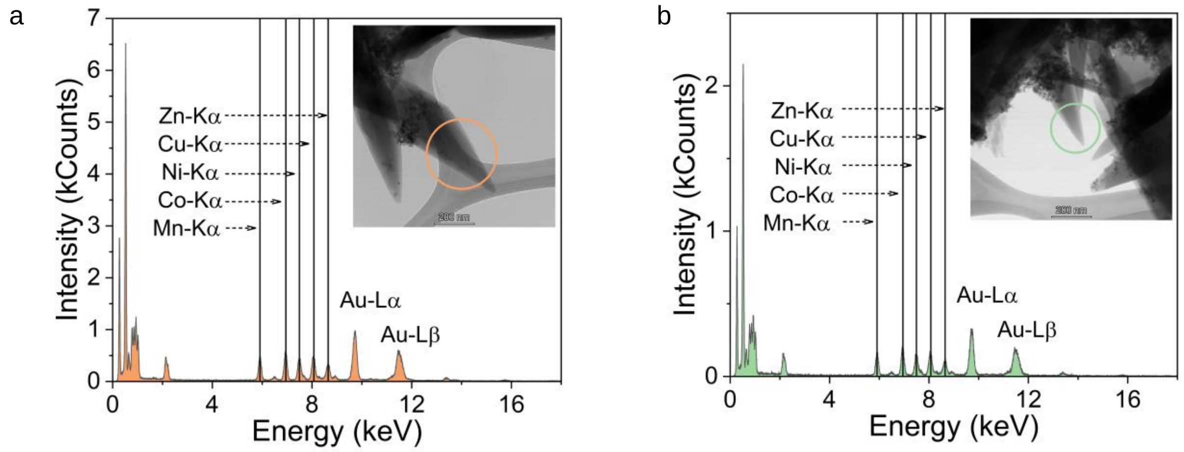

Figure S7. EDS data profile of HE-MHOF performed at the circled region on two different crystallites. In EDS data table, the presence of five different metals in atomic fraction percentage has been shown by considering Kα-line emission spectra of the transition metals. EDS data shows near homogeneous mixture of five different metals in a single crystallite sample of HE-MHOF in both cases.

EDS Table: **a**

Z	Element	Spectral Line	Atomic Fraction (%)	Atomic Error (%)	Fit Error (%)
25	Mn	Kα	18	1	0.7
27	Co	Kα	24	3	0.3
28	Ni	Kα	19	2	0.6
29	Cu	Kα	22	3	0.3
30	Zn	Kα	17	2	0.4

EDS Table: **b**

Z	Element	Spectral Line	Atomic Fraction (%)	Atomic Error (%)	Fit Error (%)
25	Mn	Kα	18	1	0.5
27	Co	Kα	23	3	0.2
28	Ni	Kα	19	2	0.2
29	Cu	Kα	22	3	0.3
30	Zn	Kα	18	2	0.3

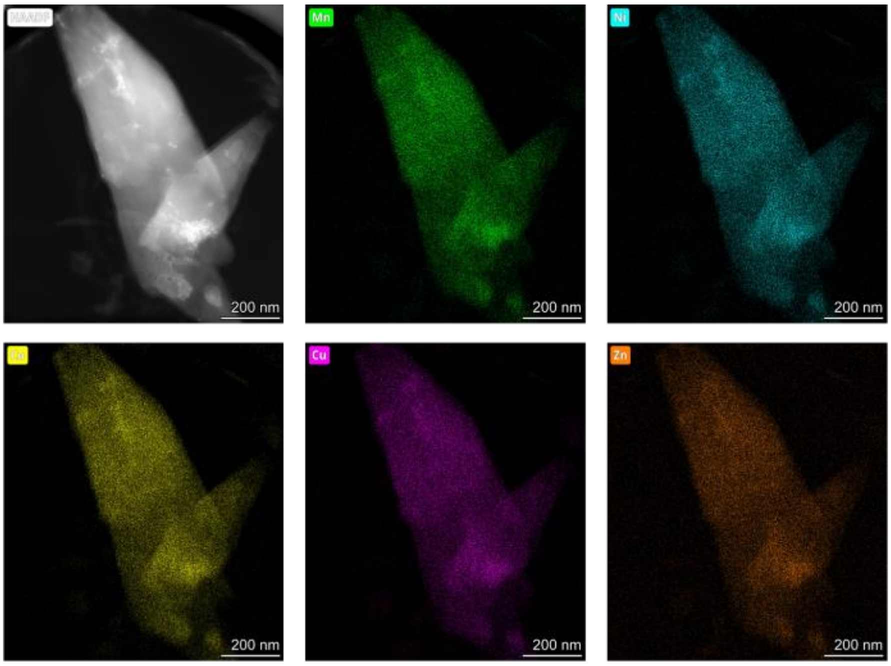

Figure S8. HAADF-STEM images and STEM-EDS mapping of the HE-MHOF. Evidence of five different metals present in same crystallite of HE-MHOF.

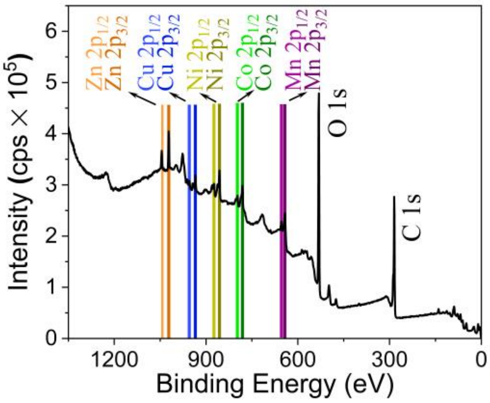

Figure S9. Survey Spectra XPS Analysis of HE-MHOF. The XPS survey spectrum obtained from HE-MHOF provides an insightful exploration of its elemental composition. Notably, this analysis discerns the presence of five distinct metals, enhancing the efficacy of surface characterization. The data strongly suggests that the surface state of HE-MHOF is characterized by a high entropy configuration, indicative of its potential to facilitate electrochemical reactions.

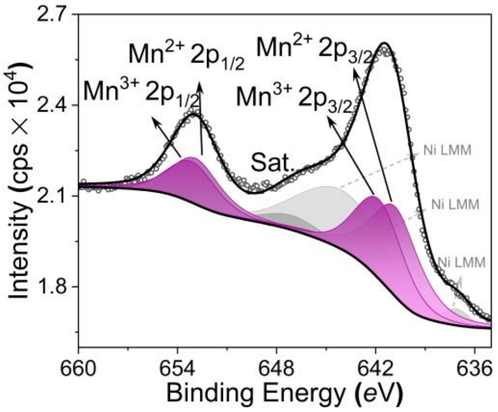

Figure S10. Deconvoluted Mn 2p XP spectrum of the HE-MHOF sample. XPS spectrum of Mn shows presence of Mn $^{2+}$ and Mn $^{3+}$ oxidation states in HE-MHOF.

Component	FWHM/eV		Binding energy/*e*V		Area CPS/*e*V	
	**Mn 2P$_{3/2}$**	**Mn 2p$_{1/2}$**	**Mn 2P$_{3/2}$**	**Mn 2p$_{1/2}$**	**Mn 2P$_{3/2}$**	**Mn 2p$_{1/2}$**
Mn^{2+}	3.50	3.50	640.70	652.50	13053.32	6658.04
Mn^{3+}	3.50	3.50	641.80	653.00	10714.51	5465.2
Mn Sat.	4.00		647.00		2037.73	
Ni LMM	6.46		643.96		13721.12	
Ni LMM	2.89		641.18		4689.36	
Ni LMM	1.85		637.01		1090.33	

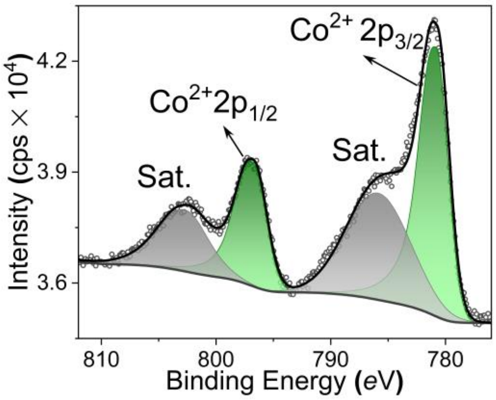

Figure S11. Deconvolution of the Co 2p X-ray Photoelectron Spectroscopy (XPS) spectrum from HE-MHOF. XPS spectra confirms that the oxidation state to be Co^{II}.

Component	FWHM / eV		Binding energy / eV		Area CPS/ eV	
	**Co 2p$_{3/2}$**	**Co 2p$_{1/2}$**	**Co 2p$_{3/2}$**	**Co 2p$_{1/2}$**	**Co 2p$_{3/2}$**	**Co 2p$_{1/2}$**
Co+2	2.41	2.68	780.70	796.60	25672.13	12618.62
Co 2P$_{3/2}$ Sat.	6.32		786.26		20390.54	
Co 2P$_{1/2}$ Sat.	4.93		802.60		9872.48	

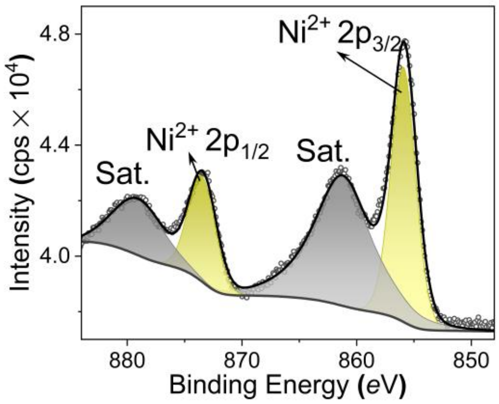

Figure S12. Deconvolution of the Ni 2p XPS spectrum derived from HE-MHOF. Ni 2p peak in the deconvoluted XP spectrum has significantly split spin-orbit components of ΔBE = 17.6 *e*V. The XPS spectra conclusively establish that Ni only present in HE-MHOF as Ni^{II}.

Component	FWHM / eV		Binding energy / eV		Area CPS/ eV	
	**Ni 2p$_{3/2}$**	**Ni 2p$_{1/2}$**	**Ni 2p$_{3/2}$**	**Ni 2p$_{1/2}$**	**Ni 2p$_{3/2}$**	**Ni 2p$_{1/2}$**
Ni +2	2.53	2.85	855.80	873.40	24267.70	11630.47
Ni 2p$_{3/2}$ Sat.	6.41		861.20		32636.54	
Ni 2p$_{1/2}$ Sat.	5.32		878.98		11286.68	

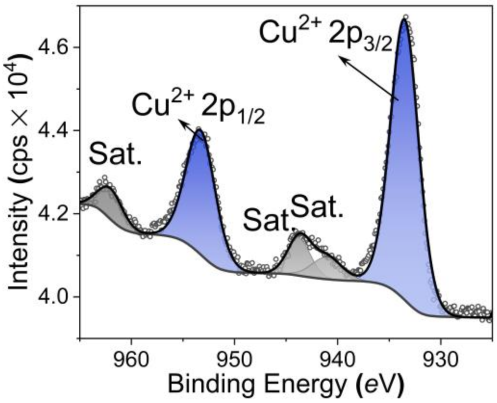

Figure S13. Deconvolution of the Cu 2p XPS spectrum derived from HE-MHOF. Cu 2p in the deconvoluted X-ray photoelectron Spectra (XPS) has Peak Separation 19.78 *e*V.

Component	FWHM/eV		Binding energy/eV		Area CPS/eV	
	Cu $2p_{3/2}$	Cu $2p_{1/2}$	Cu $2p_{3/2}$	Cu $2p_{1/2}$	Cu $2p_{3/2}$	Cu $2p_{1/2}$
Cu +2	3.31	3.5	933.5	953.28	23115.91	11178.27
Cu $2p_{3/2}$ Sat.	3.5		941.74		3872.68	
Cu $2p_{3/2}$ Sat.	1.94		943.91		1505.75	
Cu $2p_{1/2}$ Sat.	2.75		962.00		2080.44	

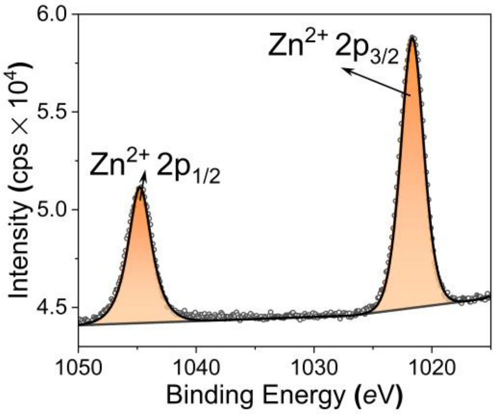

Figure S14. Deconvolution of the Zn 2p XPS spectrum derived from HE-MHOF. The XPS spectra confirms that the oxidation state of Zn in HE-MHOF is Zn^{II}.

Component	FWHM/eV		Binding energy/eV		Area CPS/eV	
	Zn $2p_{3/2}$	Zn $2p_{1/2}$	Zn $2p_{3/2}$	Zn $2p_{1/2}$	Zn $2p_{3/2}$	Zn $2p_{1/2}$
Zn +2	1.81	1.76	1021.07	1044.12	34948.87	17508.62

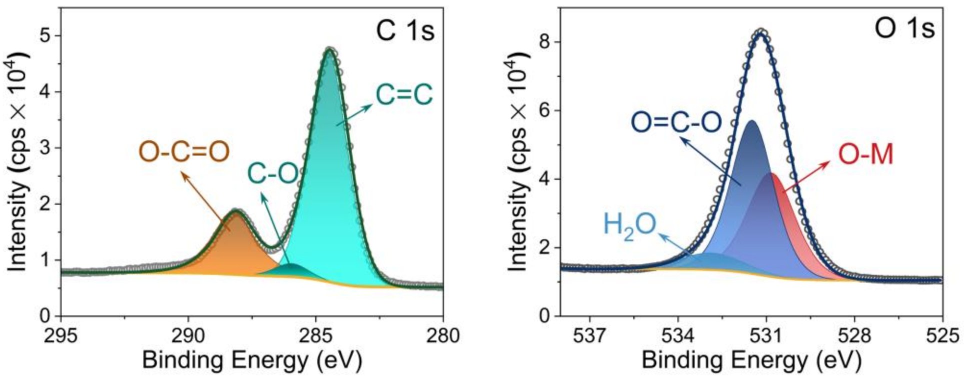

Figure S15. High-resolution XPS spectra for (left) C 1s and (right) O 1s of HE-MHOF

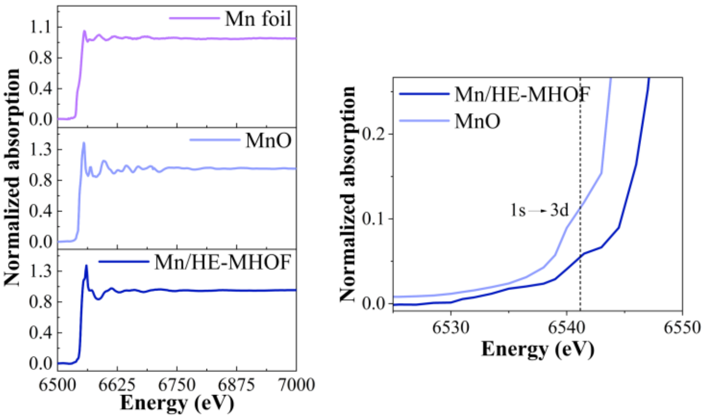

Figure S16. (Left) Mn K-edge XANES of Mn metal foil, MnO, and Mn/HE-MHOF. (Right) Magnified figures of pre-edge peaks of MnO, and Mn/HE-MHOF, respectively. There is significant edge shift between Mn K-edge of MnO and Mn/HE-MHOF. The higher energy Mn K-edge in HE-MHOF substantiates the presence of higher oxidation states of Mn, also supports the finding from high resolution XPS spectra of Mn in HE-MHOF.

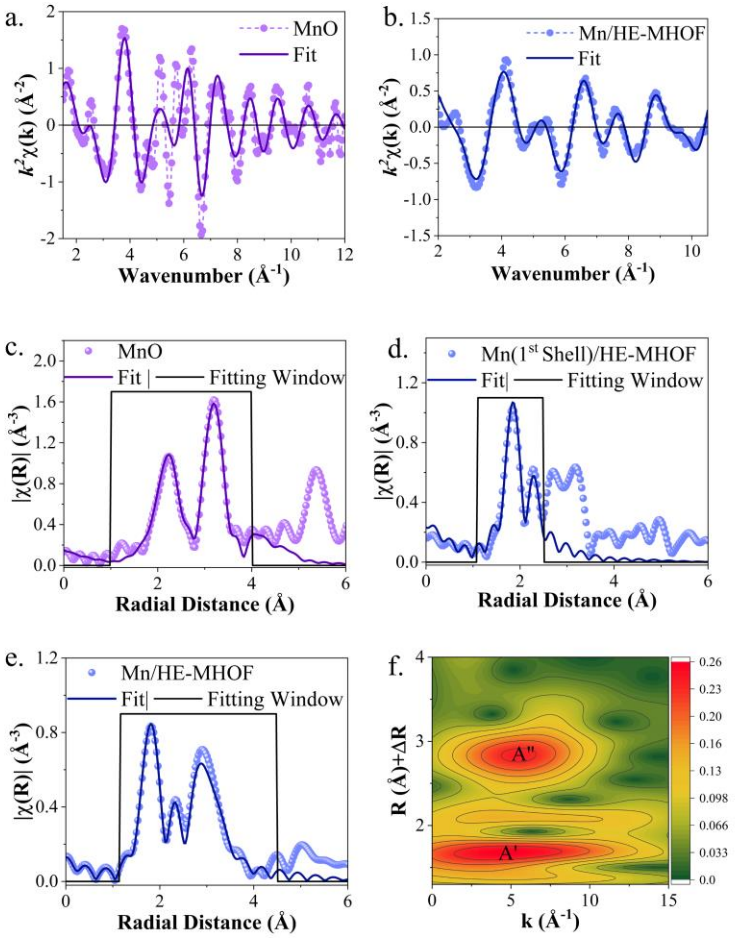

Figure S17. Mn K-edge EXAFS of (a) MnO, and (b) Mn/HE-MHOF. FT-XAFS analysis and fitting of (c) MnO (d) first coordination shell of Mn in HE-MHOF, (e) Mn/HE-MHOF, where black boxes indicate the fitting windows, and experimental and fittings are indicated as scattered and line plots, respectively. (f) Wavelet Transformation (WT) of Mn K-edge EXAFS for Mn/HE-MHOF. The intensity colour scale depicts the magnitude of the wavelet transform (a.u.). ΔR denotes the phase shift.

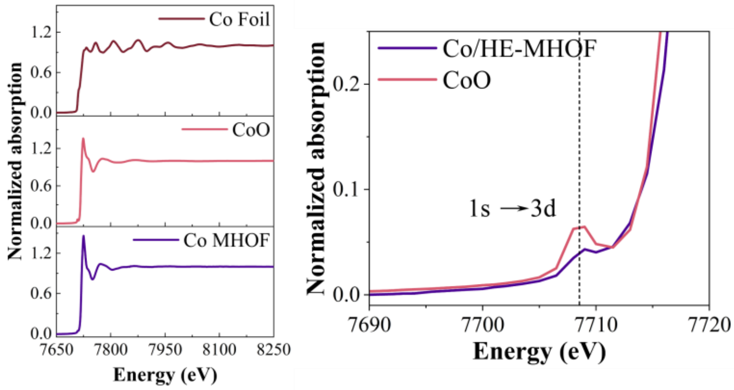

Figure S18. (Left) X-ray Absorption Near Edge Structure (XANES) spectra at the Co K-edge for different samples: Co metal foil, CoO, and Co/HE-MHOF. (Right) Magnified plots showcasing the pre-edge peaks corresponding to Co^{2+} in CoO and Co/HE-MHOF, respectively.

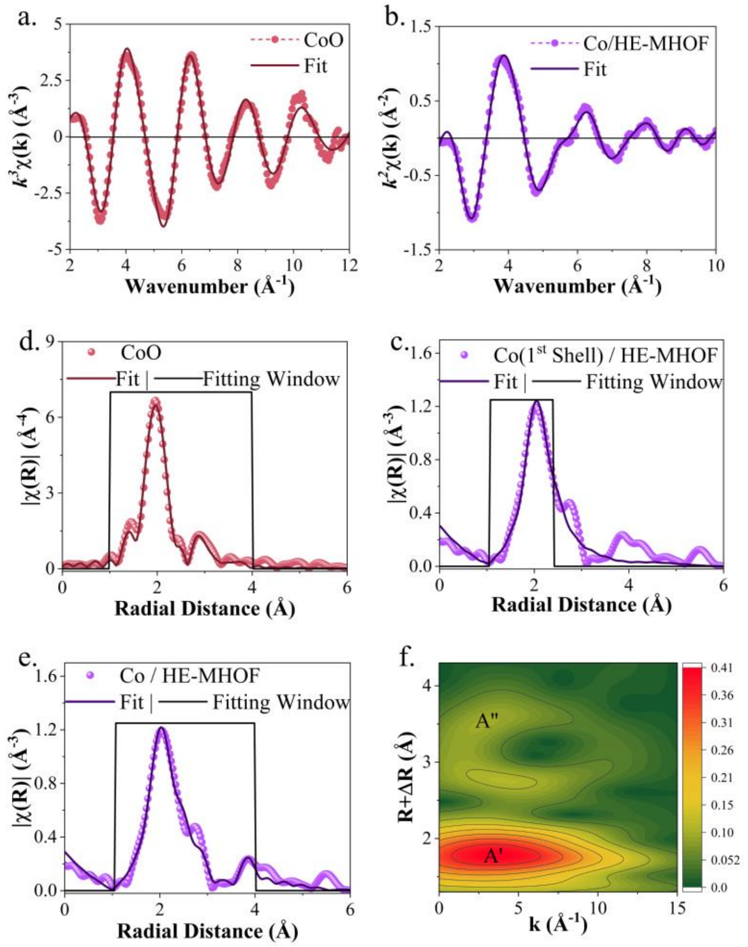

Figure S19. Extended X-ray Absorption Fine Structure (EXAFS) spectra at the Ni K-edge for (a) CoO, and (b) Co/HE-MHOF. (c) Fourier Transform (FT) analysis and fitting of (c) CoO, (d) the first coordination shell of Co atoms in HE-MHOF, and (e) Co/HE-MHOF. The regions used for fitting are denoted by black boxes, and the experimental and fitted data are represented by scattered and line plots, respectively. (f) Wavelet Transformation (WT) applied to the Co K-edge EXAFS data for Co/HE-MHOF. The intensity colour scale depicts the magnitude of the wavelet transform (a.u.). ΔR denotes the phase shift.

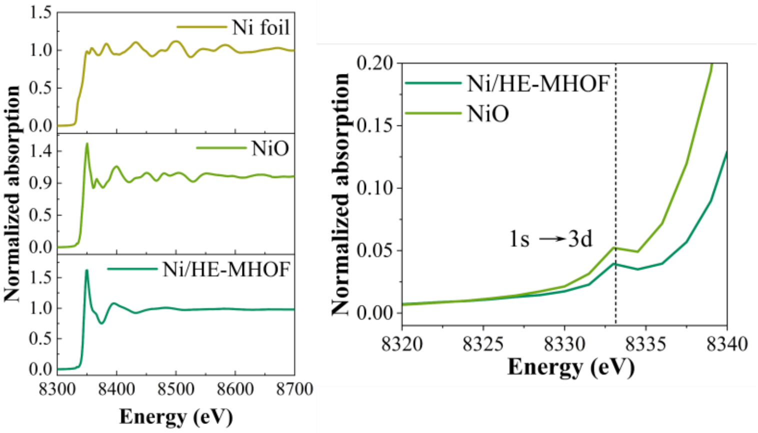

Figure S20. (Left) X-ray Absorption Near Edge Structure (XANES) spectra at the Ni K-edge for different samples: Ni metal foil, NiO, and Ni/HE-MHOF. (Right) Magnified plots showcasing the pre-edge peaks corresponding to Ni^{2+} in NiO and Ni/HE-MHOF, respectively.

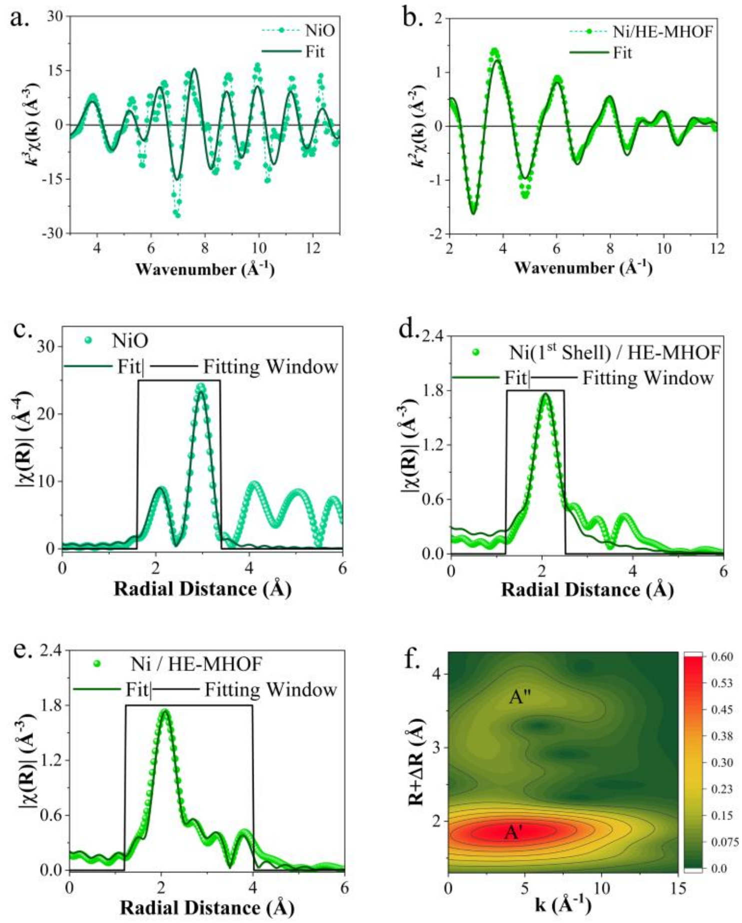

Figure S21. Extended X-ray Absorption Fine Structure (EXAFS) spectra at the Ni K-edge for (a) NiO, and (b) Ni/HE-MHOF. (c) Fourier Transform (FT) analysis and fitting of (c) NiO, (d) the first coordination shell of Ni atoms in HE-MHOF, and (e) Ni/HE-MHOF. The regions used for fitting are denoted by black boxes, and the experimental and fitted data are represented by scattered and line plots, respectively. (f) Wavelet Transformation (WT) applied to the Ni K-edge EXAFS data for Ni/HE-MHOF. The intensity colour scale depicts the magnitude of the wavelet transform (a.u.). ΔR denotes the phase shift.

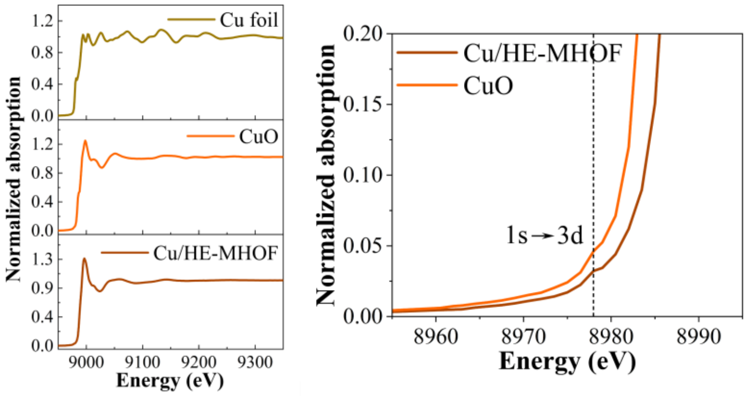

Figure S22. (Left) X-ray Absorption Near Edge Structure (XANES) spectra at the Cu K-edge for different samples: Cu metal foil, CuO, and Cu/HE-MHOF. (Right) Magnified plots showcasing the pre-edge peaks corresponding to Cu^{2+} in CuO and Cu/HE-MHOF, respectively.

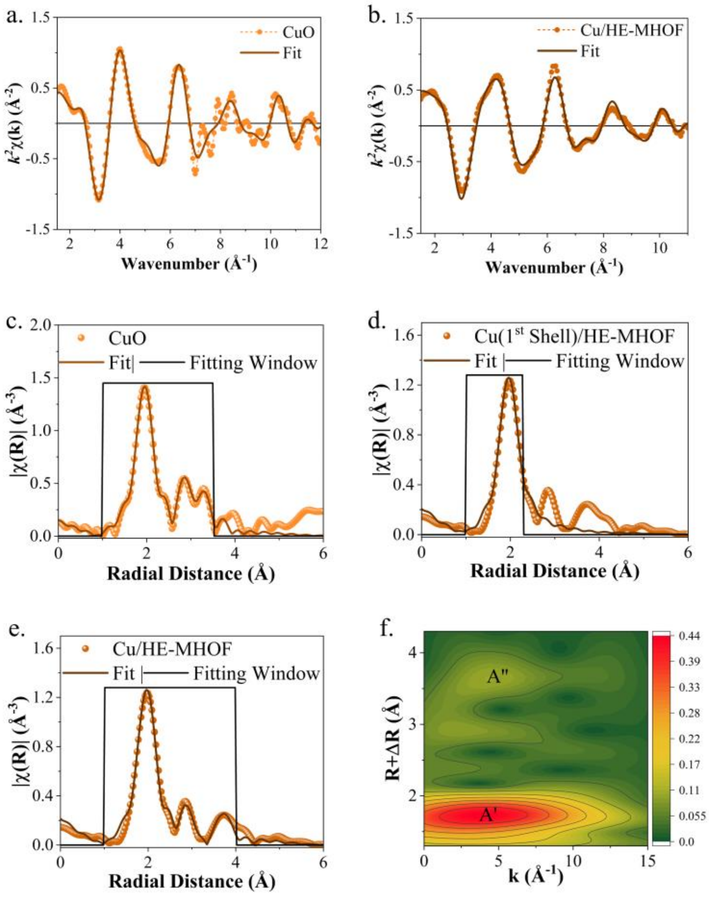

Figure S23. Extended X-ray Absorption Fine Structure (EXAFS) spectra at the Cu K-edge for (a) CuO, and (b) Cu/HE-MHOF. (c) Fourier Transform (FT) analysis and fitting of (c) CuO, (d) the first coordination shell of Cu atoms in HE-MHOF, and (e) Cu/HE-MHOF. The regions used for fitting are denoted by black boxes, and the experimental and fitted data are represented by scattered and line plots, respectively. (f) Wavelet Transformation (WT) applied to the Cu K-edge EXAFS data for Cu/HE-MHOF. The intensity colour scale depicts the magnitude of the wavelet transform (a.u.). ΔR denotes the phase shift.

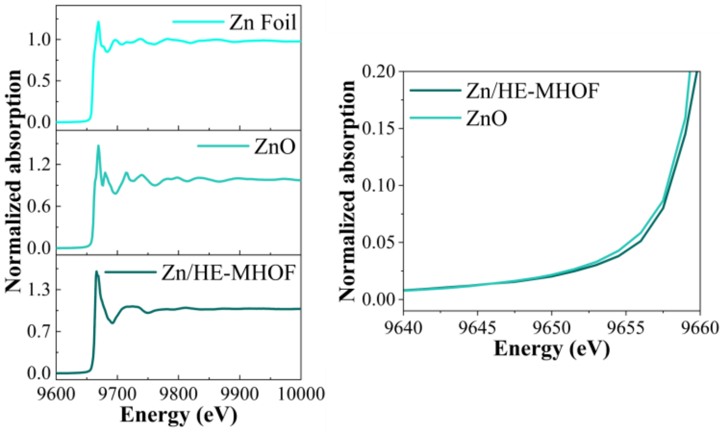

Figure S24. (Left) X-ray Absorption Near Edge Structure (XANES) spectra at the Zn K-edge for different samples: Zn metal foil, ZnO, and Zn/HE-MHOF. (Right) Magnified plots showcasing the pre-edge peaks corresponding to Zn^{2+} in ZnO and Zn/HE-MHOF, respectively.

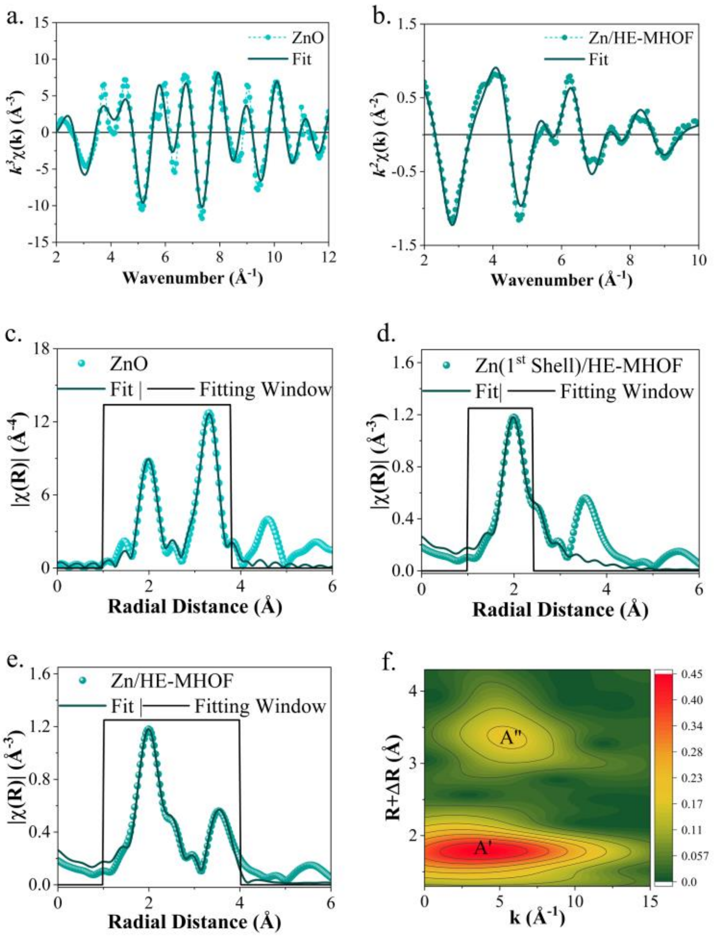

Figure S25. Extended X-ray Absorption Fine Structure (EXAFS) spectra at the Zn K-edge for (a) ZnO, and (b) Zn/HE-MHOF. (c) Fourier Transform (FT) analysis and fitting of (c) ZnO, (d) the first coordination shell of Zn atoms in HE-MHOF, and (e) Zn/HE-MHOF. The regions used for fitting are denoted by black boxes, and the experimental and fitted data are represented by scattered and line plots, respectively. (f) Wavelet Transformation (WT) applied to the Zn K-edge EXAFS data for Zn/HE-MHOF. The intensity colour scale depicts the magnitude of the wavelet transform (a.u.). ΔR denotes the phase shift.

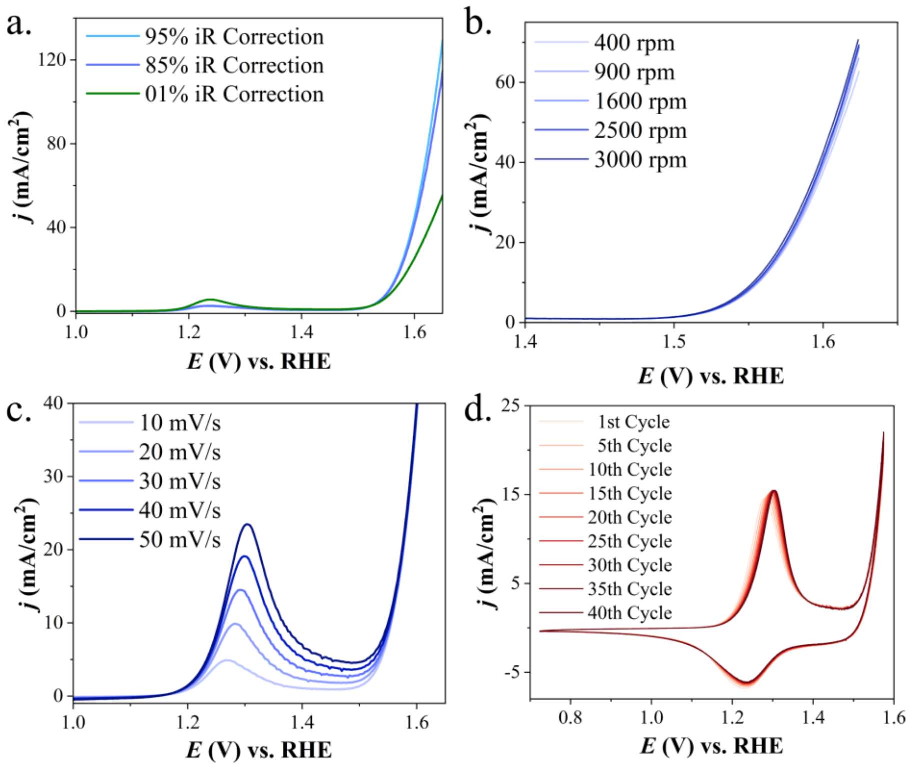

Figure S26. Polarization curves (10mV s^{-1}) of HE-MHOF in N_2 saturated 1 mol L^{-1} NaOH at 1600 rpm after different *iR* corrections. (b) Polarization curves (10mV s^{-1}) of HE-MHOF in N_2 saturated 1M NaOH at different electrode rotation rates. (c) Polarization curves of HE-MHOF in N_2 saturated 1 mol L^{-1} NaOH at 1600rpm under different scan rates. (d) Cyclic voltammogram of HE-MHOF in N_2 saturated 1 mol L^{-1} NaOH at 1600 rpm under 25 mV s^{-1} scan rate. Linear sweep Voltammetry (LSV) at 1 mol L^{-1} NaOH solution (pH = 14) in different electrode rotation substantiates that electrocatalytic oxygen evolution is not limited by mass diffusion of hydroxide ions to the electrode surface. Electrochemical Studies: cyclic voltammetry (CV) 25 mV sec^{-1} scan rate 1600 rpm of Faradaic Region in 1 mol L^{-1} NaOH medium. Depicted the quasi-reversible redox switching of the active metals in HE-MHOF.

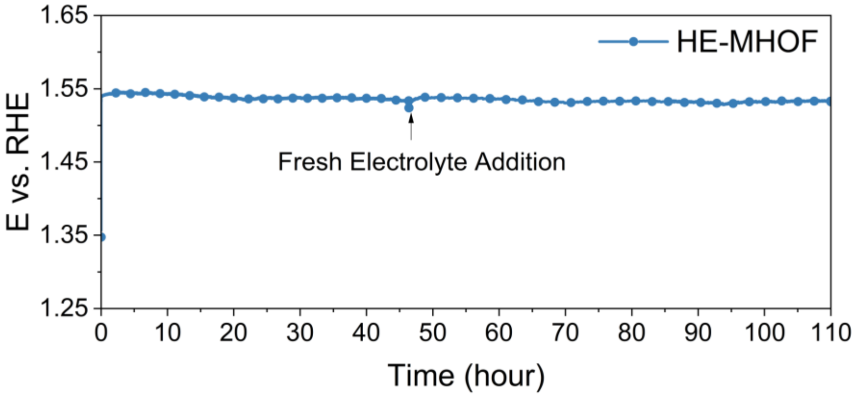

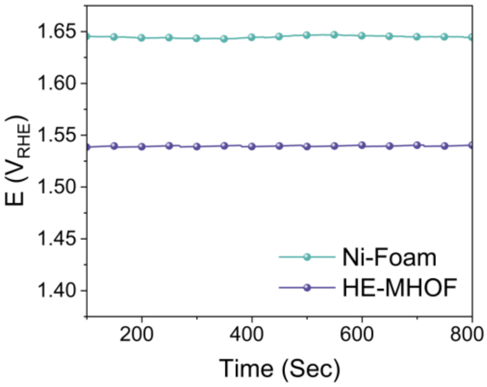

Figure S27. (top) Chrono-potentiometry stability test of HE-MHOF deposited on Ni foam substrate at a geometric current density 10 mA cm_{geo}^{-2} in 1 mol L^{-1} NaOH solution. The electrodes are prepared with standard catalyst ink consisting of HE-MHOF catalyst, 5 wt.% Nafion perfluorinated resin solution, and an additional volcan carbon black (Details in method section). (bottom) Comparison of OER activity (in terms of overpotential) between pure Ni-foam and HE-MHOF deposited substrate at a similar geometric current density of 10 mA cm^{-2} in 1 mol L^{-1} NaOH electrolyte solution.

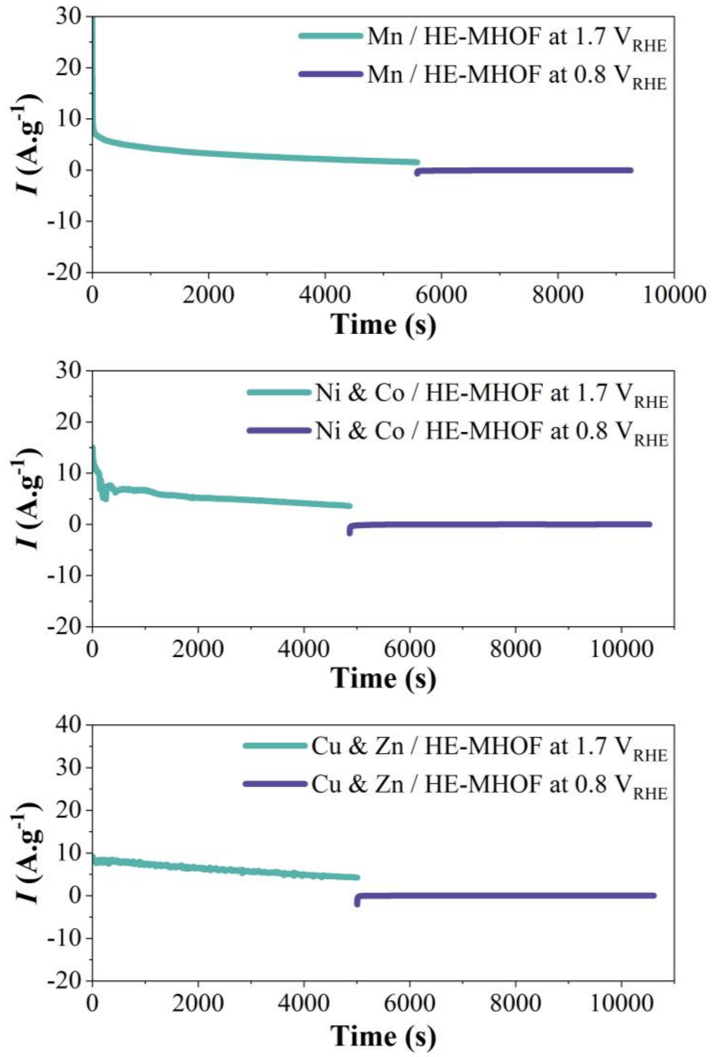

Figure S28. Chronoamperometric measurement on *in-situ* electrochemical cell to characterize the different metal centres in HE-MHOF during OER process. This setup offers the *in-situ*/operando XAS characterization between electrochemistry and spectroscopy, advancing our understanding in oxygen evolution reaction mechanism on high entropy surface.

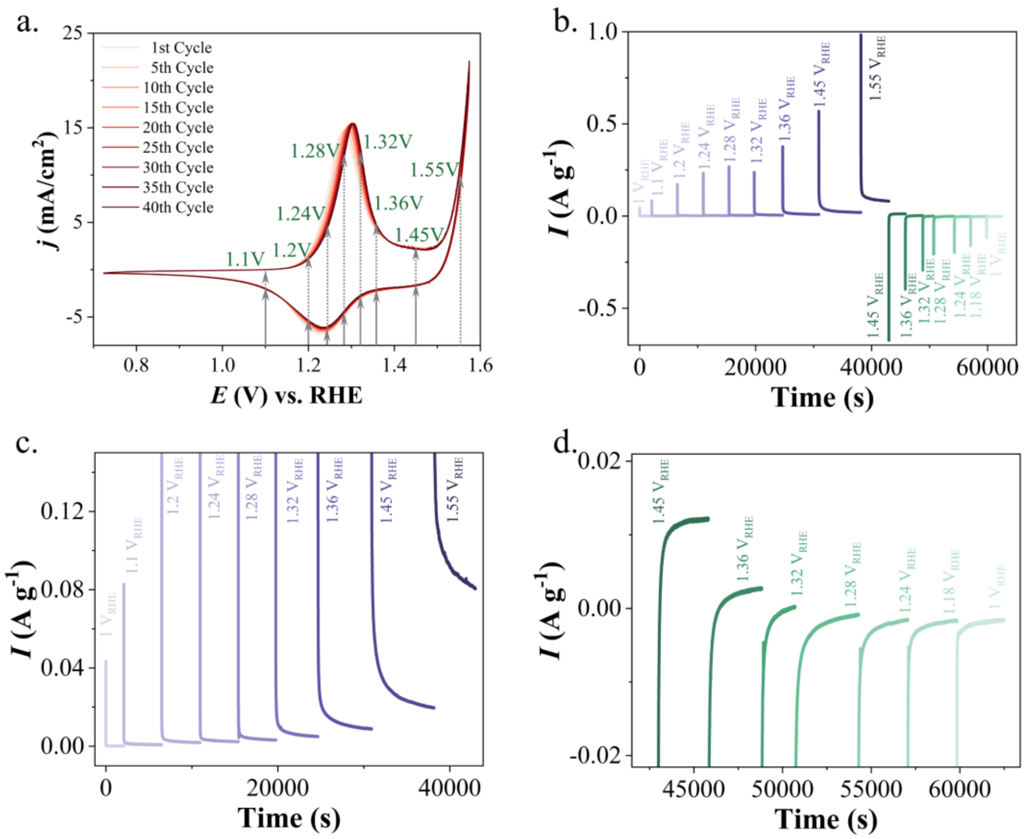

Figure S29. Chronoamperometric measurement on *in-situ* electrochemical cell to characterize the different metal centres in HE-MHOF along the faradaic redox region of the cyclic voltammogram. To resolve the partially reversible CV of HE-MHOF, *in-situ* XAS measurement had been employed for understanding the redox switching of the active metals such as Mn, Co, and Ni.

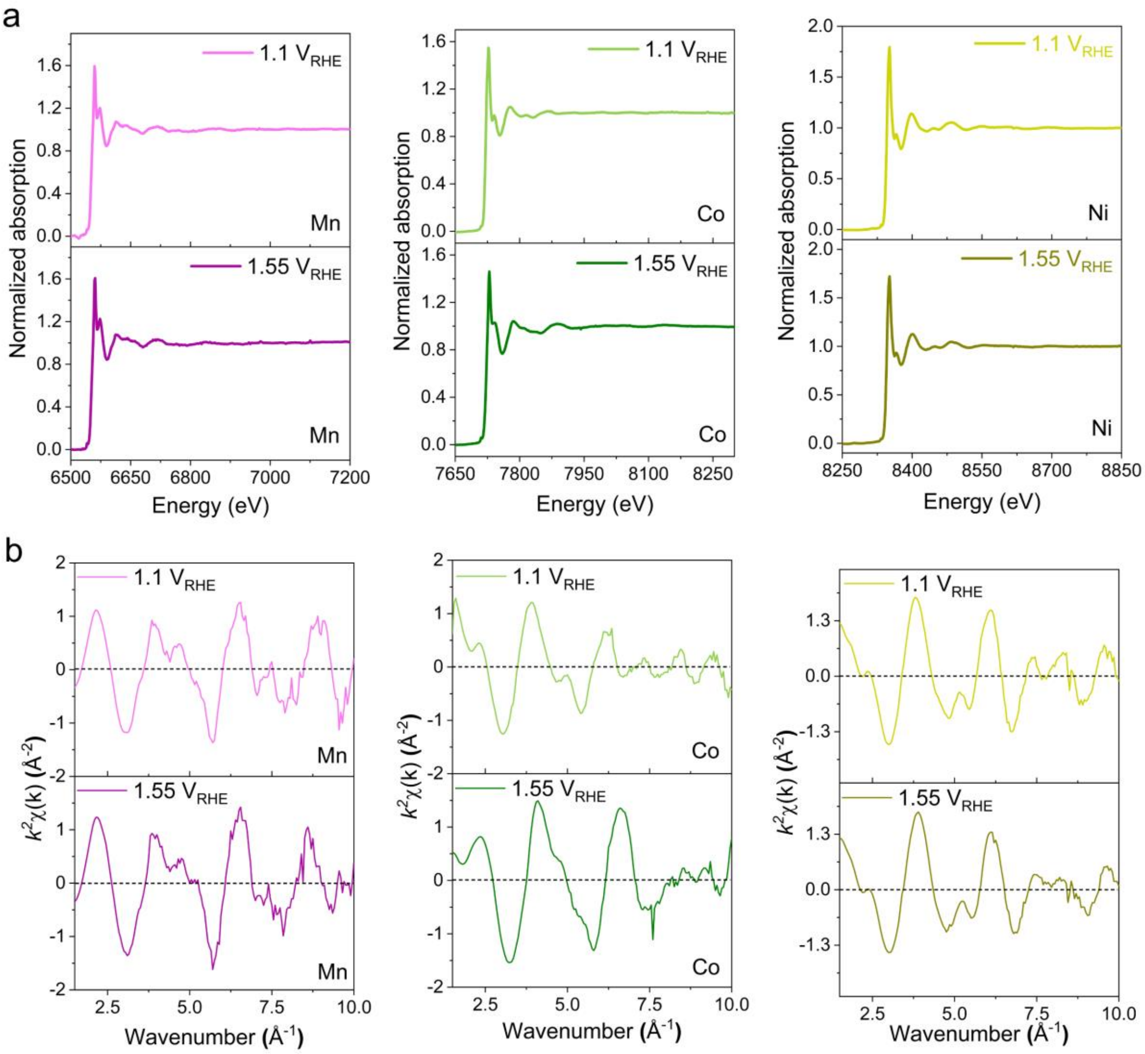

Figure S30. Operando Mn, Co, and Ni K-edges XAS characterization of HE-MHOF during OER process in 1M NaOH solution. (a) X-ray absorption spectra of Mn, Co, and Ni K-edges at the resting stage (1.1 V_{RHE}) and during OER process (1.55 V_{RHE}). (b) Mn, Co, and Ni K-edge extended x-ray absorption fine structure at the resting state (1.1 V_{RHE}) and during OER catalysis (1.55 V_{RHE}).

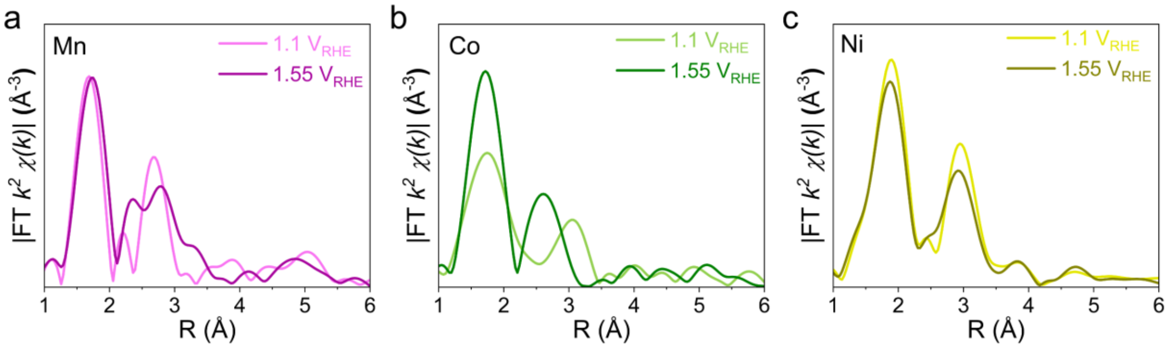

Figure S31. (a-c) Fourier transformed k^2-weighted extended x-ray absorption fine structure (FT-EXAFS) analysis of Mn, Co, and Ni K-edge of HE-MHOF recorded at different applied potential (resting stage -1.1V_{RHE}, and catalytic stage – 1.55V_{RHE}) in 1 mol L^{-1} NaOH solution.

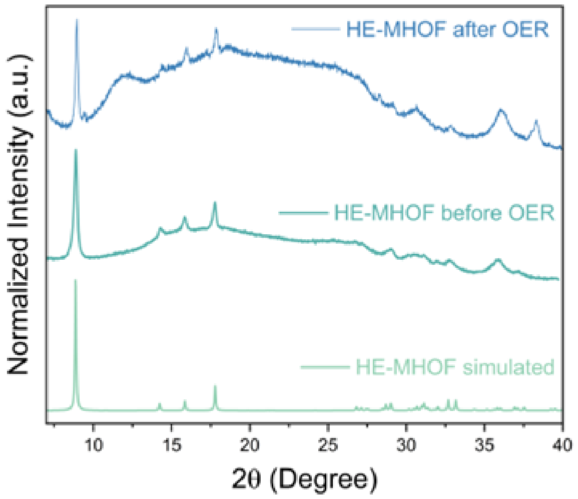

Figure S32. XRD pattern of HE-MHOF deposited on Kapton film before and after the OER process in operando XAS experiment. The XRD patterns are shown without background substruction and have contributions from the blank Kapton film.

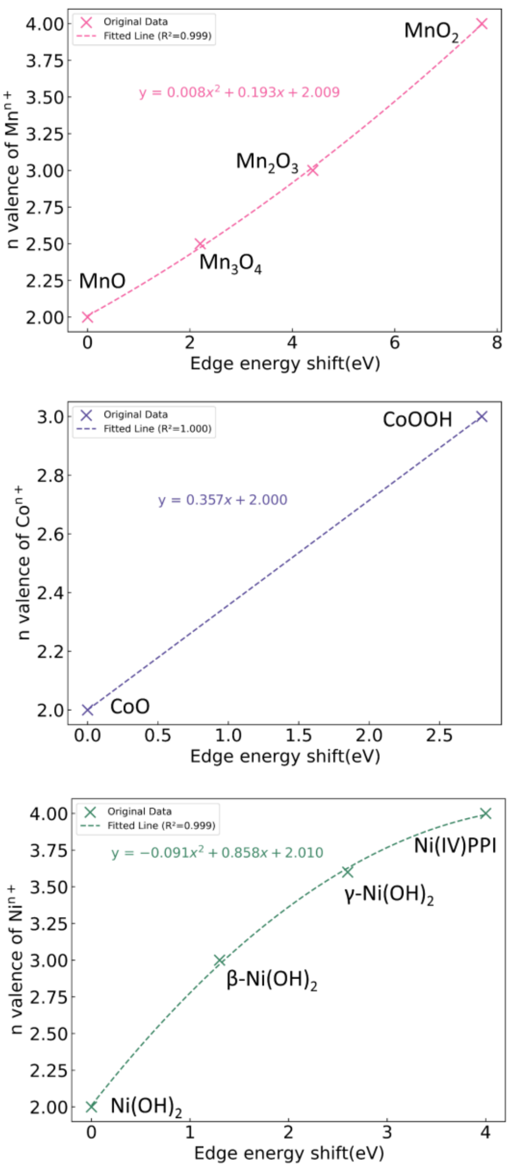

Figure S33. Valence state calibration of Mn, Co, and Ni. Chemical valence against the relative edge energy shift deduced from the reference materials. The reference Edge Energy shift data and corresponding valence state data has been fitted with a second order polynomial for Mn and Ni. In the case of Co, it has been fitted with linear fitting. The values of the edge energy of the reference materials are taken from the previous literatures and collaborated with standard database.[1–7]

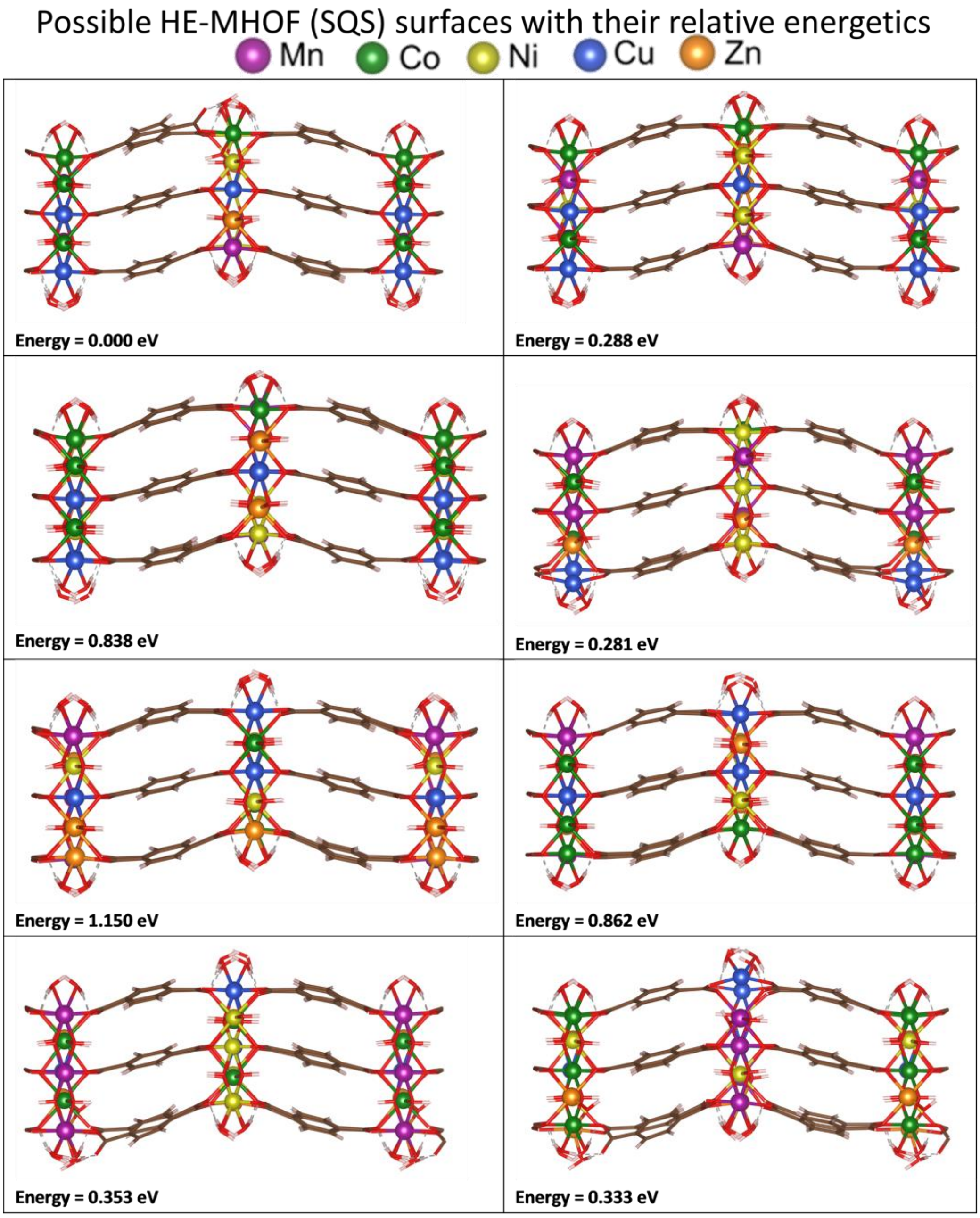

Figure S34. In our calculations, we constructed a surface based on Ni/Co-MHOF using a $2 \times 1 \times 1$ supercell consisting of 292 atoms. This supercell was simulated using sqs-generator[8] to achieve a target objective function of approximately "0". The configurations were limited to the eight bests (chosen randomly) to understand the pre-redox behaviour with changing environments around the transition metals (TMs). The relative energies of the structures are compared to the most stable structure, which is associated with 0 eV. The structural distortions are predominantly observed around the metal center, especially at the material's surface. These distortions arise from lower coordination, the Jahn-Teller effect, and the variation in the stability of oxidation states. These factors are more pronounced at the surface compared to the bulk material (Supplementary Methods; Computational Methodologies).

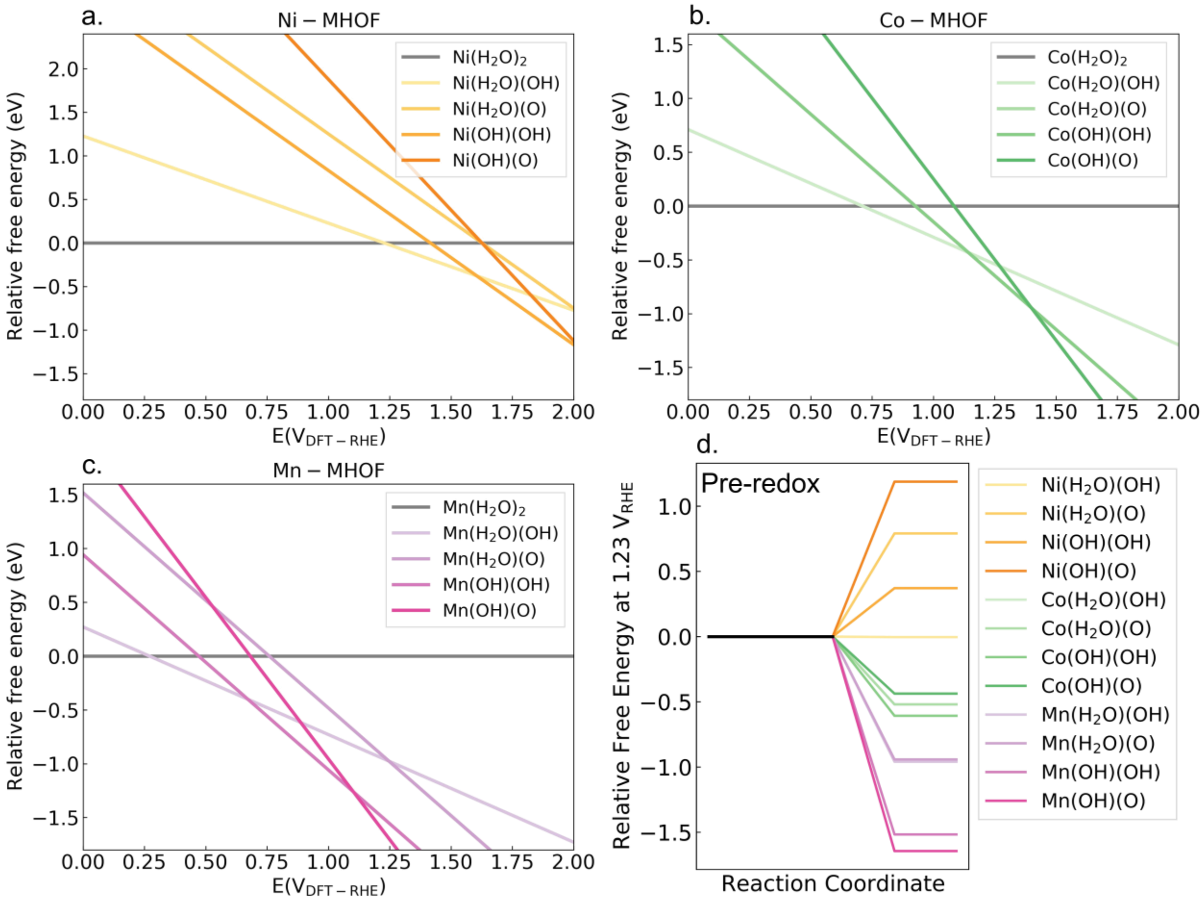

Figure S35. A Pourbaix diagram and pre-redox (at 1.23 V_{RHE}) of the relative free energy is constructed for the pristine M-MHOFs (TM = Mn, Co, and Ni) surfaces considering the most stable configuration and single coverage. The presence of Ni^{4+} hydroxide species forming on the surface of MHOFs can be seen easily but requires exceedingly high positive free energy (> 1.6 V_{RHE}) for their formation in comparison to other M-hydroxides. At > 1.0 V_{RHE}, we can observe that species such as Mn^{4+} and Co^{4+} remains stable compared to the Mn^{3+} and Co^{3+}. Though the energy barrier between Co^{4+}/Co^{3+} is relatively smaller compared to the Mn^{4+}/Mn^{3+} pair. This suggests the propensity of Mn^{IV}-hydroxides at the surface, whereas Ni^{II}-hydroxide is more stable compared to other Ni species.

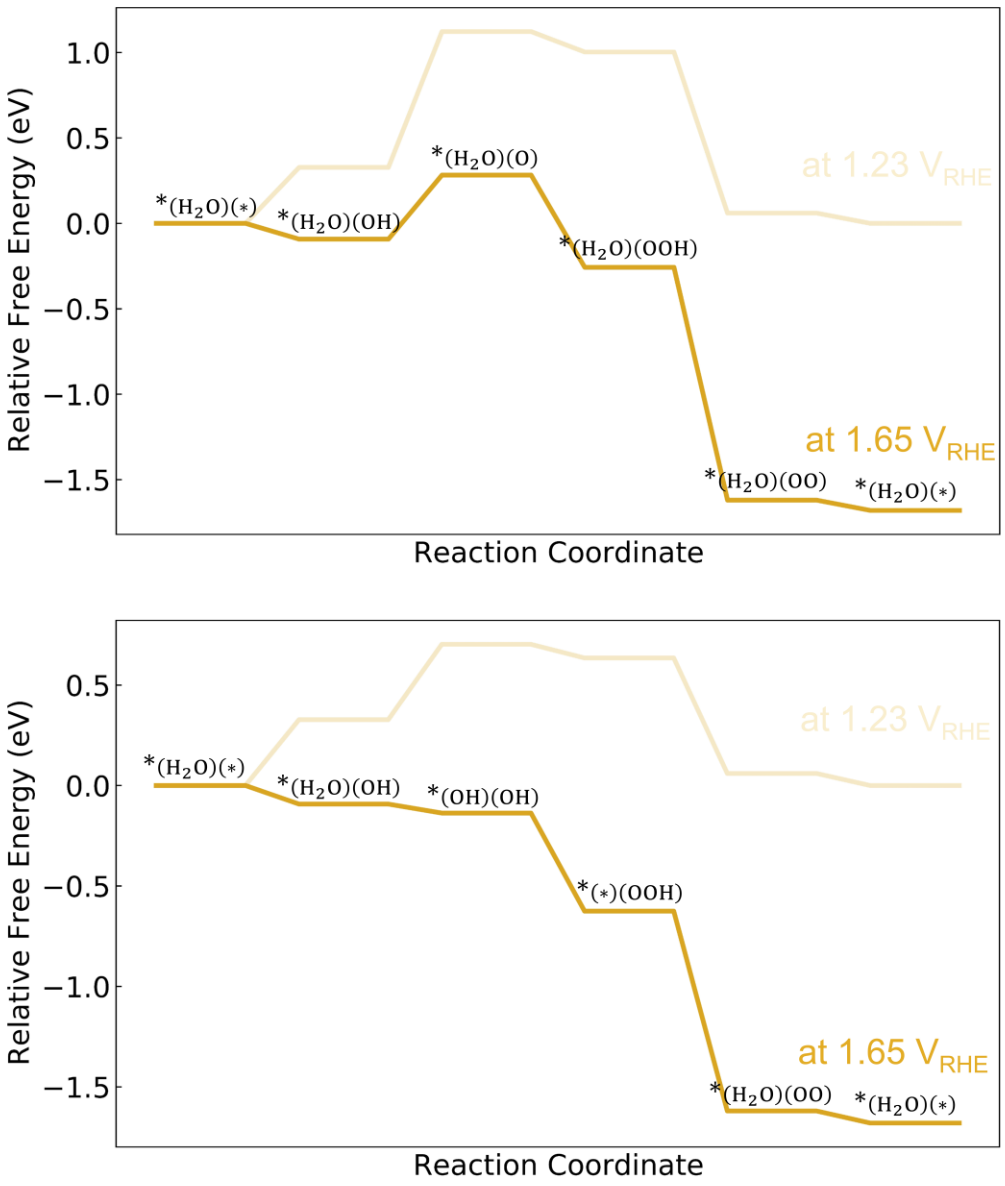

Figure S36. A free energy plot for the Ni-MHOF is constructed with potential mechanism such as Adsorbate evolution (top) vs. Intramolecular hydroxyl transfer (bottom) at thermodynamic potential (1.23 V_{RHE}) and at onset potential (1.65 V_{RHE}). The deprotonation of second water molecule to *OH (Bottom) is more favourable over further protonation of *OH (Top) to [*Ni(OH)O]. Hence, the deprotonation of second water molecule act as rate limiting step for the oxygen evolution reaction (OER), which results in the formation of [*Ni$(OH)_2$] species. The loss of proton assists the intramolecular coupling between *OH-*OH.

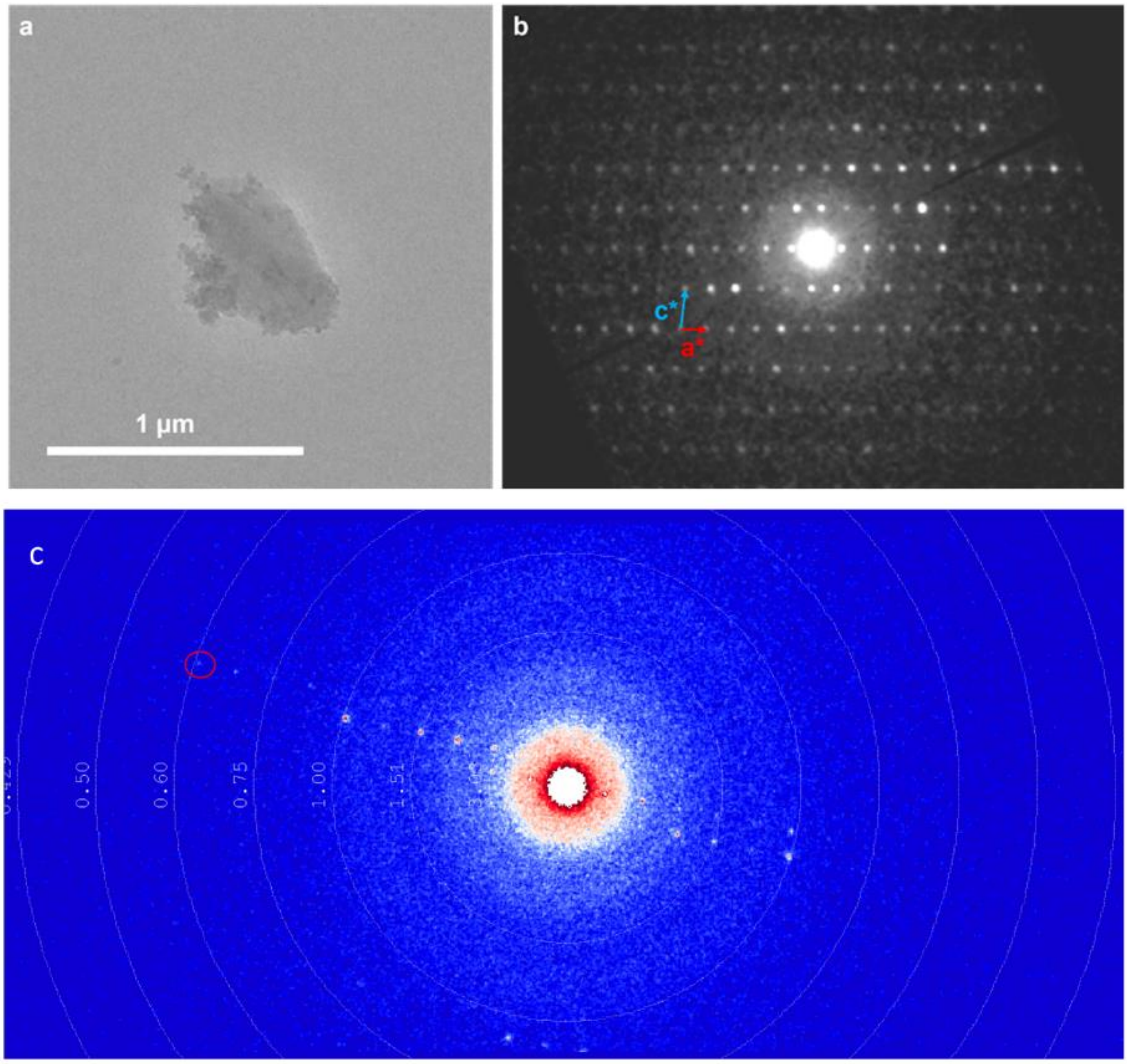

Figure S37. Electron diffraction of the HE-MHOF crystallite. a, Bright-field image of a HE-MHOF crystal. b, 0k0 zone cut out of the reconstructed three-dimensional reciprocal space indicating a high crystallinity of the sample. a*-axis shown in red, c*-axis in blue. c, HE-MHOF diffracting up to ~0.6 Å in a 3D ED measurement.

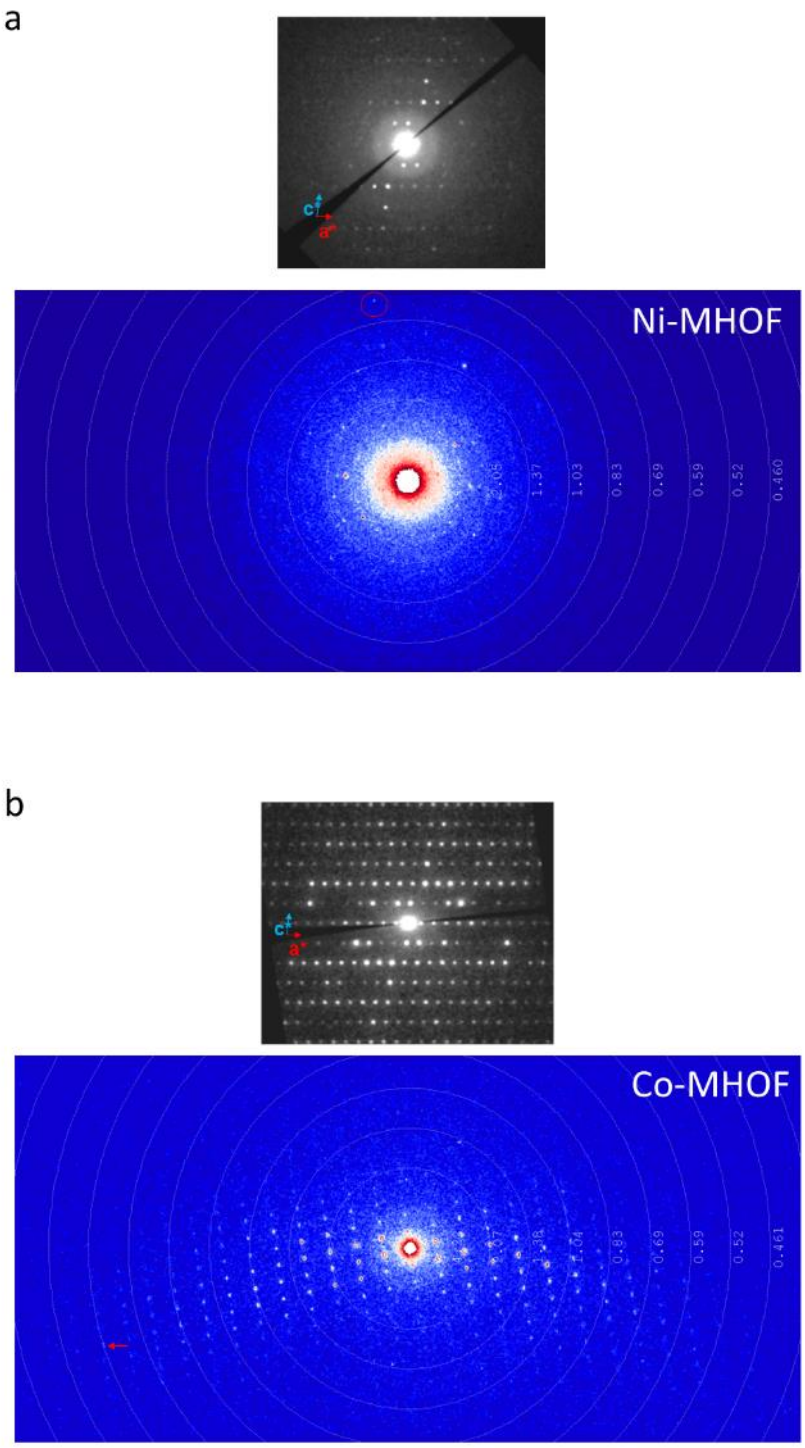

Figure S38. Electron diffraction of the Ni and Co-MHOF crystallite. (a) 0k0 zone cut out of the reconstructed three-dimensional reciprocal space indicating a high crystallinity of the Ni-MHOF. a*-axis shown in red, c*-axis in blue. and Ni-MHOF diffracting up to ~0.9 Å in a 3D ED measurement. (b) 0k0 zone cut out of the reconstructed three-dimensional reciprocal space indicating a high crystallinity of the Co-MHOF. a*-axis shown in red, c*-axis in blue. and Co-MHOF diffracting up to ~0.5 Å in a 3D ED measurement.

Supplementary Methods

Materials:

All the chemicals and solvents were commercially available and used without any further purifications. Metal chloride salts such as $MnCl_2$, $NiCl_2.6H_2O$, $CoCl_2.6H_2O$, $CuCl_2.6H_2O$, $ZnCl_2.4H_20$ are purchased from ABCR Chemicals, and Sigma Aldrich. Therephthalic acid (99+%) is purchased from thermos scientific Chemicals. IrO_2 (99.9 % - metal basis) was purchased from Premion, Thermoscientific. Solvents such as DMF, EtOH, MeOH, Acetone etc. were purchased from Carl Roth.

Powder X-ray Diffraction:

The samples into Borat capillaries with internal diameter 0.5mm. PXRD was carried out at room temperature using D8 Bruker Discover diffractometer (Bruker, AXS, Germany) equipped with a LYNXEYE XE-T detector equipped with a Cu-sealed tube (wavelength λ = 1.5418 Å) at 40kV and 40mA. The sample was measured from 5° to 40° at a step size of 0.009° (2θ). Pawley Refinement of the PXRD data was performed within the TOPAS V6 suite.[9] The High-Entropy Metal Hydroxide Organic Framework (HE-MHOF) was refined using Powder X-ray Diffraction (PXRD) to derive cell parameters. Initially, we selected the triclinic space group for this refinement, considering possible symmetry breaks from parent Ni-MHOF while forming an HE matrix. We began by refining the cell parameters based on those established for Ni-MHOF. This strategy proved effective in precisely determining the HE-MHOF's parameters. Once these parameters were refined experimentally, they were fixed to serve as a foundation for subsequent simulations.

Thermogravimetric Analysis:

TGA was performed on dry powders (~6 mg) using heat flux TGA-DSC 3+ (Mettler-Toledo). All measurements were carried out under a continuous nitrogen flow of 10ml min^{-1}. As a reference, an empty Aluminium-100µL crucible was used. The samples were heated with a heating rate of 10K min^{-1} from 25°C to 600°C. Subsequently, the samples were allowed to cool down under continuous nitrogen gas flow.

Dynamic Vapor Sorption Measurement:

DVS with water was carried out with a DVS Resolution Dual Vapor Gravimetric Sorption Analyser (Surface Measurement Systems). The measurement was performed on ~20 mg sample. To ensure a dry sample, a preheating step was included. The method dynamic vapor sorption (DVS) is applied to determine the amount of adsorbed vapor within a solid sample at constant temperature. To obtain an adsorption isotherm, the vapor pressure outside the sample will be stepwise increased. Thereby, it may take minutes up to hours until an equilibrium stage is reached for each step. During the whole process, the sample weight is gravimetrically monitored in dependence of the vapor pressure, which enables the determination of the adsorbed amount. In detail, the sorption isotherm is obtained by plotting the adsorbed mass (adsorbed mass related to the dry sample mass) in dependence of the relative pressure.

Transmission Electron Microscopy (TEM), Scanning Transmission Electron Microscopy (STEM), and Energy-Dispersive X-ray Spectroscopy (EDS):

Transmission electron microscopy (TEM) images were obtained using a Talos F200S Microscope (Thermo Fisher Scientific) operated at 200 kV. In this microscopy technique, a beam of electrons is transmitted through a specimen to form an image. A Ceta 16M camera (TEM mode) and a HAADF and BF detector (STEM mode; Scanning Transmission Electron Microscopy) were used to capture the images. The specimens were prepared by dropping 10 µL sample solutions (1 mg/mL in EtOH) onto a 3 mm gold grid (lacey, 400 mesh) and leaving them to air-dry at room temperature. A Super-X G2 detector equipped with two silicon drift detectors (SDD) was used during the TEM analysis for energy dispersive X-ray spectroscopy (EDS) to determine the elemental composition of the HE-MHOF with counting time of 60 seconds. EDS was also used for chemical characterization with compositional mapping in STEM mode. Velox software Version 3.3 is used for imaging and evaluation. (HAADF: High-angle annular dark-field) (BF: Bright field)

X-ray Photoelectron Spectroscopy (XPS):

XPS was measured on a K-Alpha™ by X-ray Photoelectron Spectrometer System (Thermo Scientific) with Hemispheric 180° dual focus analyzer with 128-channel detector. The X-ray monochromator used micro focused Al-Kα radiation. For the measurement, the powder samples were passed and loaded on carbon tape, then pasted onto sample holder for measurement. The data was collected with an X-ray spot size of 400 µm, 20 scans for the survey, and 50 scans for the regions. In high resolution XPS spectra the five transition- metals can have a form of spin-split into two spin-orbit peaks $2p_{1/2}$ and $2p_{3/2}$, in addition to that there are shake up satellite peaks denoted as '*sat.*'.

Inductively Coupled Plasma Mass Spectrometry (ICP-MS):

Consumables (50 mL tubes, pipette tips) were cleaned with diluted double-subboiled nitric acid (w = approx. 3 %) prior to analysis. Type I reagent-grade water (18.2 MΩ cm, HQ-water) was obtained from a Milli-Q Integral water purification system (Merck-Millipore, Darmstadt, Germany). Analytical reagent-grade nitric acid (w = 65 %, Merck-Millipore) was purified by double sub-boiling using a sub-boiling distillation system. Approx. 20 mg of the HE-MHOF-bulk-sample was dissolved in 2 mL of double-subboiled nitric acid (w = 65 %). The degraded HE-MHOF sample was diluted with HQ-water to a final volume of approx. 50 mL and weighted. ICP-MS measurement of the HE-MHOF-sample was performed in triplicates after the dilution by a factor of 1000 with diluted nitric acid (w = 2 %). Multi-elemental analyses (incl. Mn, Co, Ni, Cu and Zn) of the diluted HE-MHOF samples were performed using an ICP-MS (ICAP-Q, Thermo Scientific, Bremen, Germany) coupled to an ESI SC-4 DX FAST autosampler (Elemental Scientific, Omaha, USA). The ICP-MS instrument was optimized in STD-mode in a daily routine using a tuning solution (Thermo Scientific) to obtain maximum signal intensity while maintaining high stability and low oxide and double-charged rates.The multi-elemental quantification of the diluted the HE-MHOF-sample was accomplished by external calibration (9-point calibration ranging for Mn, Co, Ni and Cu from 0.1 ng mL^{-1} to 150 ng mL^{-1} and for Zn from 1 ng mL^{-1} to 1500 ng mL^{-1}) with standards prepared from a multielement stock solution (ICP multielement standard Merck VI, 30 Elements in nitric acid 10–1000 mg L^{-1}) including 1 ng mL^{-1} indium (prepared form an In stock solution, Merck-Millipore) as internal normalization standard. Furthermore, the performance of the multi-elemental analysis was monitored with in-house quality control solution (incl. Mn, Co, Ni, Cu and Zn) prepared in nitric acid (w = 2 %). Finally, the presence of each metal calculated in percentage $\mathrm{M}\ \% = \frac{\mathrm{M}}{\mathrm{Mn+Co+Ni+Cu+Zn}}$ from the determined metal mass fractions. The Limits of Detection (LOD) and Limits of Quantification (LOD) were calculated according to DIN ISO 11843-2. Therefore, the LOD is defined as 3 x standard deviation of the blank and the LOD is defined as 10 x standard deviation of the blank. Total combined uncertainties for multi-element analysis were calculated using a simplified Kragten approach.

**Parameter**	**ICAP-Q (Thermo Scientific)**
Sample introduction	Spray chamber
Nebulizer	PFA – 100 µL (Elemental Scientific)
Interface	Ni skimmer + sampler
RF power (W)	1550
Nebulizer gas (L min^{-1})	1.06
Auxillery gas (L min^{-1})	0.65
Isotopes	^{55}Mn, ^{59}Co, ^{60}Ni, ^{62}Ni ^{63}Cu, ^{65}Cu, ^{66}Zn, ^{67}Zn
Dwell time (s)	0.01
Sweeps	10
Runs	5

Electrochemical Measurements:

Electrodes for cyclic or linear voltammetry were prepared by drop-casting an ink containing IrO_2 or HE-MHOFs containing powders on a glassy carbon electrode. Typically, the catalyst ink was prepared with 5mg of the catalyst and 1mg of volcan carbon black in 980 µl water/ethanol (1:1) with 20 µl 5wt% Nafion solution, followed by sonication in an ultra-sonic water bath for 30 min. The glassy carbon electrode surface (0.07 cm^2) was drop casted with 25 µg cm$_{geo.}^{-2}$ of net catalyst loading. Furthermore, for the long term stability measurements, the nickel foam substrate (1 × 1 cm^2) was spray coated with the similar catalyst ink by using an air brash, in which the catalyst mass loading was quantified as 4 mg cm^{-2}. The electrochemical measurements were performed using a glass electrochemical cell with Hg/HgO reference electrode and Pt counter electrode in a rotating disk electrode set-up in N_2 saturated 1 mol L^{-1} NaOH (99.99% purity, Sigma-Aldrich) electrolyte. The potential

was controlled using a Biologic VSP-3e potentiostat. LSV and CV measurements were performed at a scan rate of 5 and 25 mV s^{-1}, respectively. Ohmic losses in the electrochemical measurements were corrected by subtracting the ohmic drop from the measured electrolyte resistance, which is determined by high frequency alternating current impedance, where 85% *iR*-corrected potentials are denoted as *E* (V). All measured potentials were converted to values relative to a RHE according to the equation: E (V) = E_{ref} + $E_{(ref\ versus\ RHE)}$, with $E_{(ref\ versus\ RHE)}$ the potential difference between the reference electrode and RHE.

X-ray Absorption Spectroscopy (XAS):

The K-edge X-ray absorption spectra of the transition metals of HE-MHOF were collected at the BAMline[10] of the BESSY-II (Berlin, Germany), operated by the Helmholtz-Zentrum Berlin of Materials and Energy. The incident x-ray beam was provided by a super bend magnet (7 T) source and subsequently energetically narrowed by two monochromators, a Double Multilayer Monochromator (DMM) and Double Crystal Monochromator (DCM). The DMM is used at a mirror to supress higher harmonics, and the DCM is equipped with Si (111) crystals which is used to fine scan the energy range with a resolution of $\Delta E/E=2x10^{-4}$. The measured current was 20 nA at ionisation chamber, this corresponds to the photon flux of 1.25×10^{11} photons/sec. The final beam size on the sample was 4 mm (horizontal) x 2 mm (vertical). The XAS measurements were performed transmission mode containing both X-ray absorption near edge structure (XANES) and extended X-ray absorption fine structure (EXAFS). Nitrogen-filled ionization chambers (1.5 bar, 5 cm, and 15 cm long) were used in transmission geometry. All metal foils (Mn, Co, Ni, Cu, Zn) were placed between the ionization chambers to calibrate the energy to the K-edge of the metals. The measurement protocol for both ex-situ and operando was the following: 10 eV steps until 20 eV before the edge, followed by 0.25 eV steps until 20 eV above the edge and 2 eV steps until 200 eV above the edge. From then on equidistant k-steps were taken (every 0.06 Å) until 16 Å. The acquired spectra were extracted, calibrated, and normalized using the IFFEFIT software package containing ATHENA and ARTEMIS software.[11] The Fourier Transformations are made in k-space and the resulting R-space is used for fitting with the model structures. Furthermore, the wavelet transformation of EXAFS spectra to resolve backscatters energy dependency in k-space. The software module for the calculation and graphical output of EXAFS wavelet analysis has been developed by Funke et al.[12,13]

Operando measurements were performed either using an *in-situ* Spectro-Electrochemical flow cell purchased from redox.me or an upgraded version of the latter including slight modifications self-made flow cell. The working electrode were prepared by manually spraying a HE-MHOF catalyst ink on pieces of glassy carbon electrode or of Kapton foil with a layer of conductive carbon (DuPont Kapton 200RS100) and additionally sputter-coated in-house with a ~100nm layer of gold surrounding the area on which the catalyst was coated. For the XAS measurements in transmission mode, the catalyst concentration was increased in the ink, and was set to 8 mg/mL_{ink}. The catalyst was further sprayed on the gold coated Kapton substrate with a high loading of ~6 mg/cm^2. In the case of electrodes prepared for the fluorescence measurements, the catalyst loading on the glassy carbon substrate was set to only of ~0.4 mg/cm^2. All the measurements were performed in 1M NaOH solution. White an Ag/AgCl electrode was used as the reference electrode, and platinum wire was used as counter electrode. Further, the flow of the electrolyte was controlled by a syringe pump operating at 0.4 mL/min, which also help to reduce the accumulation of the O_2 bubbles along the beam path. Finally, XAS measurements were acquired which performing a chronoamperometric technique at different applied potential.

Three-Dimensional Electron Diffraction (3D ED):

Three-dimensional electron diffraction data (3D ED) was measured on a Rigaku XtaLAB Synergy-ED with a Rigaku HyPix-ED detector.[14,15] Samples were prepared by grinding the material slightly between two class plates and depositing the powder on lacey carbon TEM grids. Using a Gatan Elsa (Model 698) cryo-transfer sample holder, the samples were measured at 175K with a wavelength of 0.0251 Å. The total electron dose during dataset acquisition was 0.29 – 0.60 e/A^2 per dataset. The recorded diffraction patterns indicate a single crystalline nature of the HE-MHOF, Ni-MHOF and Co-MHOF. After reconstructing the three-dimensional reciprocal space with CrysAlisPro,[16] the unit cells were determined and averaged between the grains of the respective samples.

Computational Methodologies:

First-principles calculations are performed within the projector-augmented wave (PAW) methods as implemented in the Vienna *ab initio* simulation package (VASP)[17,18] by using the generalized gradient approximation (GGA) of Perdew-Burke-Ernzerhof (PBE).[19,20] A plane wave cut-off of 500 *e*V is used for the truncation of wave functions in our calculations. A **k** point grid of $4 \times 8 \times 1$ is used for the structural optimization of unary MOFs structures. The conjugate gradient algorithm is used for ion and cell relaxation. An energy convergence criterion is set to 10^{-06} *e*V during structural optimization and 10^{-08} *e*V for the calculation of total energies and electronic structure. During the structural optimization, the forces convergence

criterion is set to 0.01 $eV/Å$ for individual atoms. The HE-MHOF structure is modelled with the special quasi-random structure generator code (sqsgenerator)[21], and a Γ-point **k** point grid is used to investigate relative energetics. To model a HE-MHOF structure, we used a $2 \times 5 \times 2$ unit cell (using lattice parameters obtained from Pawley refinement) containing 880 atoms (with 80 metals in equimolar ratio). This structure is relaxed using a Γ-point k mesh, and simulated XRD is compared with experimental XRD. An SQS surface was subsequently constructed using a $2 \times 1 \times 1$ supercell comprising 292 atoms. The Brillouin zone is sampled by a Γ-centered $2 \times 1 \times 1$ **k** point grid during the surface calculations. The simulation cell size was chosen such that a and b are greater than 12 Å, and a vacuum region of more than 15 Å was included to prevent interactions between periodic images. However, a better structure with a larger unit cell model is always possible purely based on the SQS model. Still, after DFT relaxations, local relaxation might yield an energetically more favourable structure. Hence, more configurations are needed for the investigation of relative energetics (eight chosen based on objective function). The selection of structure is based on the minimization of the objective function ($\mathcal{O}$), which is defined as

$$\mathcal{O}(\sigma) = \sum_i w^i \, \Sigma_{\xi,n} P_{\xi n} |\alpha_{\xi n}^{\sim i} - \alpha_{\xi n}^{i}(\sigma)| \qquad (1.1)$$

where $\alpha(\sigma)$ is defined as Warren-Cowley Short Range Order (SRO) parameter for some given σ configuration for some coordination sphere 'i', ξ and n represents the two species with their corresponding mole fractions in the sample. Similarly, P and w define pair weights (used to differentiate bonds) and shell weights (account for coordination shells) parameters used for tuning against the target objective ($\alpha^{\sim i}$).[21] The value of $\alpha(\sigma)$ signifies clustering ($\alpha(\sigma)>0$), ordering ($\alpha(\sigma)<0$) and randomness ($\alpha(\sigma)\approx 0$) in a crystal structure. Here, the target objective value of "0" is chosen to govern the randomness in the considered sample space.

The free energy change for an electrochemical reaction is calculated by the following expression:

$$\Delta G = \Delta E_{DFT} + \Delta E_{ZPE} - T\Delta S + \Delta G_U \qquad (1.2)$$

Where, ΔE_{DFT} is the energy change (for reactant → product) for an electrochemical reaction, T represents the temperature (298.15 K) and ΔS represents the entropy contribution, ΔE_{ZPE} represents change in zero-point energy to the free energy for the reaction and ΔG_U represents the over-potential of the reaction. The phonon frequency calculation utilizing the finite difference approach computes the adjustments to the DFT energies, incorporating the zero-point energy correction and entropy correction at a temperature of 298.15 K.

Tafel slope analysis:

From the discussion by Bockris,[22,23] assuming a multi-step electrochemical reaction:

$$A + e^- \leftrightarrow B \text{ (step 1)}$$

$$B + e^- \leftrightarrow C \text{ (step 2)}$$

$$\ldots$$

$$\tau \{I + e^- \leftrightarrow J \text{ (step } n_i)\}$$

#RDS, repeating τ times

$$\ldots$$

$$X + e^- \leftrightarrow Y \text{ (step } n_n)$$

We assume quasi-equilibrium condition, the initial steps prior to the RDS happens very rapidly in both forward and backward directions, and the RDS is assumed to be dominated the reaction rate. The tafel slope can be derived as:

$$\frac{2.303RT}{\alpha F} = \frac{60}{\alpha} \text{ (mV/dec)} \qquad (2.1)$$

$$\alpha = \frac{n_p}{\nu} + n_r\beta \qquad (2.2)$$

where F is Faraday constant (96485 C/mol), R is the ideal gas constant (8.314 J/K/mol), T is the temperature, n_p is the number of electrons transferred before the RDS, n_r is the number of electrons transferred in the RDS, ($\nu = 1$) in case of OER represents the number of times RDS occurs for completion of one full OER cycle, β is the symmetry coefficient of the RDS, for a multi-step reaction. Usually, symmetry coefficient is ascribed to an arbitrary value of 0.5, both experimentally and theoretically.[24,25]

Valance State Determination:

Mn, Co, Ni K-edge positions of HE-MHOF from the resting stage (1.1 V_{RHE}) to catalytic stage (1.55 V_{RHE}) at 1M NaOH was determined and correlated to their average oxidation states from the comparison to reference compounds presented in Figure S33. K-edge positions of each transition metals were obtained by the integral method.[26] An average edge energy can be defined as:

$$E_{edge} = \frac{1}{\mu_1 - \mu_2} \int_{\mu_1}^{\mu_2} E(\mu)\, d\mu \qquad (3.1)$$

In the equation 3.1, the inverse function $E(\mu)$ of the XANES spectres, $\mu(E)$, is integrated. The same value of E_{edge}can be obtained by:

$$E_{edge} = E(\mu_1) + \frac{1}{\mu_1 - \mu_2} \int_{E(\mu_1)}^{E(\mu_2)} \mu_2 - \mu(E)\, dE \qquad (3.2)$$

For the experimental XANES spectres, the function $\mu(E)$ is a discrete, and generally non-monotonic function. Consequently, the integration limits of the inverse function, $E(\mu_1)$ and $E(\mu_2)$, are not uniquely defined. Therefore, numerical integration is performed by using a function $\hat{\mu}(E)$, defined as:

$$\hat{\mu}(E) = \begin{cases} \mu_1, & \text{for } \mu(E) < \mu_1 \\ \mu_2, & \text{for } \mu(E) > \mu_2 \\ \mu(E), & \text{else} \end{cases} \qquad (3.3)$$

So, for all the energy points with the interval $[E_1, E_2]$:

$$E_{edge} = E_1 + \frac{1}{\mu_1 - \mu_2} \int_{E_1}^{E_2} \mu_2 - \hat{\mu}(E)\, dE \qquad (3.4)$$

The calculated E_{edge} value has no dependence on the boundary conditions, $E_1$and E_2, if they are chosen in a sensible way, where $\mu(E_1) < \mu_1$ and $\mu(E_2) > \mu_2$. However, the E_{edge} value directly depends on the $\mu_1$and μ_2 parameters. The value of $\mu_1$was chosen to ensure that the magnitude of the pre-edge feature does not affect the calculated E_{edge} value, and the value of μ_2 was chosen to ensure that the determined edge position is not strongly affected by the magnitude of the white-line intensity. So, the $\mu(E)$ is following the maximum of the rising part of the edge jump.

OER reaction mechanism:

Under the acidic conditions, the overall oxygen evolution reaction can be well described by the below mechanism,[27–29]

$2H_2O(l) \rightarrow O_2(g) + 4H^+ + 4e^-; \Delta G_0 = 4.92\, eV$ (4.1)

And the possible reaction mechanism is shown below.

$H_2O\,(l) + * \rightarrow {}^*OH + H^+ + e^-$ (4.2)

${}^*OH \rightarrow {}^*O + H^+ + e^-$ (4.3)

$H_2O\,(l) + {}^*O \rightarrow {}^*OOH + H^+ + e^-$ (4.4)

${}^*OOH \rightarrow * + O_2\,(g) + H^+ + e^-$ (4.5)

where * represents the surface site for catalysis, and *g* (and *l*) refers as gas (and liquid) phase. The species adsorbed on the active site of surface are represented by the $^*A$ (A = OH, O, and OOH). The above mechanism involves the four-proton transfer for catalyzing the oxygen evolution reaction and based on the Computational Hydrogen Electrode approach. [30] Here, the calculation of Gibbs free energy for the pair $(H^+ - e^-)$ is calculated by assuming the equilibrium $\frac{1}{2}H_2 \rightleftharpoons H^+ + e^-$ at standard conditions ($T = 298.15$ K, $p_{H_2} = 1\, bar$ and $pH = 0$). Although, the reactions involving somewhat different acidic medium ($pH \neq 0$) and alkaline conditions involves correction to free energy due to change in the pH and formation of OH^- ions in the reaction's mechanism (shown below).

$* + OH^- \rightarrow OH^* + e^-$ *R*1 (4.6)

${}^*OH + OH^- \rightarrow {}^*O + H_2O\,(l) + e^-$ *R*2 (4.7)

${}^*O + OH^- \rightarrow {}^*OOH + e^-$ *R*3 (4.8)

${}^*OOH + OH^- \rightarrow * + O_2(g) + H_2O\,(l) + e^-$ *R*4 (4.9)

Whereas the overall reaction is given by

$$4OH^- \rightarrow O_2\ (g) + 2H_2O\ (l) + 4e^- \quad (4.10)$$

Therefore, the determinization of the free energy of OH^- ions become crucial to investigate the oxygen evolution reaction in the alkaline conditions. Now, the Gibbs free energy equation for the above OER mechanism in alkaline conditions follows the below mentioned equations:

$$\Delta G_{R1} = \mu_{*OH} - \mu_{*} - (\mu_{OH^-} - \mu_{e^-}) \quad (4.11)$$

$$\Delta G_{R2} = \mu_{*O} + \mu_{H_2O(l)} - \mu_{*OH^-} - (\mu_{OH} - \mu_{e^-}) \quad (4.12)$$

$$\Delta G_{R3} = \mu_{*OOH} - \mu_{*O} - (\mu_{OH^-} - \mu_{e^-}) \quad (4.13)$$

$$\Delta G_{R4} = \mu_{O_2(g)} + \mu_{*} + \mu_{H_2O\ (l)} - \mu_{*OOH} - (\mu_{OH^-} - \mu_{e^-}) \quad (4.14)$$

Where μ simply represents the chemical potential for the individual species (s) involves in the reaction mechanism and can be represented by the following relation:

$$\mu_s = E_{DFT,s} + E_{ZPE,s} - T \times S_s \quad (4.15)$$

Where, E_{DFT} represents the total energy (DFT), E_{ZPE} represents the zero-point energy and S (and T) represents the entropy (and temperature) contribution. As the overall total free energy for water oxidation reaction is $4.92\ eV$ (ΔG_0).[31] The chemical potential for all the intermediate species involves in the OER mechanism ($R1$-$R4$) can be calculated directly from DFT calculations apart from H_2O (*l*), O_2 (*g*) and $[OH^- + e^-]$ pair. As, we can describe the equilibrium conditions for H^+ and OH^- pair at any pH by the below relation:

$$H_2O\ (l) \rightleftharpoons H^+ + OH^- \quad (4.16)$$

Or their chemical potential simply relates to the following expression:

$$\mu_{H_2O\ (l)} = \mu_{H^+} + \mu_{OH^-} \quad (4.17)$$

Which on further simplification (by adding and subtracting the μ_{e^-}) gives

$$\mu_{OH^-} - \mu_{e^-} = \mu_{H_2O\ (l)} - (\mu_{H^+} + \mu_{e^-}) \text{ or as per CHE} \quad (4.18a)$$

$$\mu_{OH^-} - \mu_{e^-} = \mu_{H_2O\ (l)} - \mu_{\frac{1}{2}H_2} \quad (4.18b)$$

Now, the chemical potential of H_2O (*l*) and O_2 (*g*) are needed to be describe the ΔG_{Tot} $(= \Delta G_{R1} + \Delta G_{R2} + \Delta G_{R3} + \Delta G_{R4})$ for the overall OER. For O_2 (*g*), we can simply assume the chemical potential for oxygen gas. For the H_2O molecule, it becomes problematic to accurately describe the chemical potential because of the expression 4.1. To further simplify and equate the above relation, the chemical potential for H_2O can be calculated in the presence of solvent condition which leads to the following relation:

$$\mu_{H_2O\ (l)} = E_{DFT,H_2O\ (l)} + E_{ZPE,H_2O\ (g)} - T \times S_{H_2O\ (g)} \quad (4.19)$$

Where, the $E_{DFT,H_2O\ (l)}$ is calculated with solvent correction ($E_{DFT,H_2O\ (g)} + E_{Sol,H_2O}$).

Now, the chemical potential for H_2 (*g*) as per CHE approach is describes as $H^+ + e^- = 1/2H_2$. We use the approach of reversible hydrogen electrode as our reference electrode to describe the equilibrium of H^+ ions with respect to H_2 (*g*) under the condition of p_{H_2} = 1 bar and T = 298.15 K but under different *pH* conditions compared to the actual experimental conditions (where, *pH* = 0). So, the chemical potential for H^+ ions is,

$$\mu_{H^+} = \mu_{H^+}^{RHE} \quad (4.20)$$

And the chemical potential for the e^- is expressed as

$$\mu_{e^-} = \mu_{e^-}^{RHE} - eU_{RHE} \quad (4.21)$$

Now, U_{RHE} is the potential of reference electrode related to RHE. Hence, the expression for chemical potential of OH^- and e^- reduces to the following expression:

$$\mu_{OH^-} - \mu_{e^-} = \mu_{H_2O\ (l)} - (\mu_{H^+}^{RHE} + \mu_{e^-}^{RHE} - eU_{RHE}) \quad (4.22a)$$

$$= \mu_{H_2O\ (l)} - (\mu_{\frac{1}{2}H_2} - eU_{RHE}) \quad (4.22b)$$

Synthesis Procedures:

Synthesis of $M_{mix}(OH)_2$ (Mmix = Mn, Ni, Co, Cu, Zn). In a typical synthesis, all the metal chloride salts of equimolar amount (0.5 mmol each) were dissolved in 10 ml of water and separately doubled stoichiometric amount of NaOH was dissolved in 10 ml of water, followed by mixing the solutions and starring for 1 hr at room temperature. Afterward, the precipitate was filtered by buchner funnel and vacuum pump. In addition, washing the residue several times with water, at the same time checking the pH of the filtrate solution with pH papers until the pH of the filtrate reaches to neutral pH. Finally, the product was dried under vacuum.

Synthesis of HE-MHOF The HE-MHOF was synthesized form mixed metal hydroxide $M_{mix}(OH)_2$ (M_{mix} = Mn, Ni, Co, Cu, Zn) and the terephthalic acid linker under a conventional hydrothermal condition. For Instance, freshly prepared $M_{mix}(OH)_2$ precursor (0.3 mmol) and terephthalic acid (0.3 mmol) was added in *N,N*-dimethylformamide (6ml), water (4ml), EtOH (2ml) in 20 ml Teflon caped high pressure vessel and the reaction was carried out at 120° C for 48 hr. After cooling down to room temperature, the product was collected by centrifugation and further washed with DMF (2 times) and methanol (2 times) and further dried at the room temperature.

Synthesis of monometallic-MHOF The monometallic MHOFs was synthesized by following a previously reported protocol. [6] Freshly prepared Ni-hydroxide (0.5 mmol) and terephthalic acid (0.5 mmol) was added to 16 ml DMF, 1 ml water, 1ml Ethanol solutions mixture and the reaction was carried out in a solvothermal procedure. The product was further washed with DMF three times and EtOH (one time) and collected as mentioned in the previous synthesis method. Afterward, the all the MHOFs was characterized and used for the electrochemical measurements.

Table S1

Crystallographic details and Pawley Refinement parameters of High Entropy MHOF (HE-MHOF). PXRD patterns and calculated patterns shown in Figure S3

Property	HE-MHOF
Chemical formula	$C_4H_3Mn_{0.2}Co_{0.2}Ni_{0.2}Cu_{0.2}Zn_{0.2}O_3$
Formula weight	159.36
Crystal System	Triclinic
Space Group	*P-1*
a (Å)	20.082(1)
b (Å)	3.164(1)
c (Å)	6.371(1)
α (º)	90.50(1)
β (º)	95.46(1)
γ (º)	88.96(1)
Z	2
Temperature (K)	298(2)
X-ray wavelength (Å)	1.5418
2θ range (º)	7-40
R_p	1.07
R_{wp}	1.44
R_{exp}	0.85
GOF	1.68

Table S2

Sample	XANES Energy Range (*e*V)	Edge Position (E_o) (*e*V)	White Line Intensity (a.u.)	Normalized XANES Spectrum
Mn/HE-MHOF	6400-7200	6546	1.66	Figure S16
Mn/MnO	6400-7200	6544.5	1.85	Figure S16
Co/HE-MHOF	7600-7800	7718.0	1.46	Figure S18
Co/CoO	7600-7800	7717.5	1.36	Figure S18
Ni/HE-MHOF	8200-8400	8344.5	1.65	Figure S20
Ni/NiO	8200-8400	8344.2	1.45	Figure S20
Cu/HE-MHOF	8850-9050	8987	1.33	Figure S22
Cu/CuO	8850-9050	8983.5	1.21	Figure S22
Zn/HE-MHOF	9550-9750	9663.5	1.58	Figure S24
Zn/ZnO	9550-9750	9662	1.5	Figure S24

Table S3

Measured reference material and HE-MHOF samples were fitted with MnO (*cubic*) from Materials Project[32] and simulated HE-MHOF structure (Figure S34), respectively. Results from Mn K-edge EXAFS fitted parameters, including scattering path, coordination number (N), interatomic distance (R), inter atomic distance from the model (R_{eff}), Debey Waller factor (σ^2), amplitude reduction factor ($S_O{}^2$), energy shift parameter (ΔE), and R-factor, are given below. Mn K-edge first-shell EXAFS of HE-MHOF was fitted with the fixed amplitude reduction factor ($S_O{}^2$), where $S_O{}^2$ was obtained from the reference material MnO. Finally, the total Mn-K edge EXAFS fit of HE-MHOF was also performed with fixed $S_O{}^2$ and N from the first shell fit. Parentheses indicate a standard deviation of the last digit of fitted parameters, and the fixed parameters in the fitting are underlined. The Hanning window type, with dk = 1 is chosen to perform the Fourier transformation over the selected k-space range between 2 to 12 Å^{-1} for MnO, and 2 to 11 Å^{-1} for HE-MHOF, respectively. The fit to the model structure was performed between 1 and 4 Å for MnO. The fit to the HE-MHOF model structure was performed between 1.3 and 2.5 Å for the first coordination sphere, and between 1.3 and 4.5 Å for the additional coordination spheres. Δ in scattering path denotes obtuse triangle type. (Fitted plots in Figure S17)

Sample	***Scattering path***	***N***	***R(Å)***	***R_{eff}(Å)***	***σ^2 (Å^2)***	***$S_O{}^2$***	***ΔE (eV)***	***R-factor***
	Mn-O	6	2.20(1)	2.22	0.007			
MnO	Mn-Mn	8	3.14(1)	3.14	0.007	0.7(0.07)	3.3(0.8)	0.017
	Mn-O	8	3.79(4)	3.83	0.015			
Mn/HE-MHOF (1st Mn sphere)	Mn-O_α	3.5	1.91(3)	1.94	0.002(2)	0.7	1.7(4.3)	0.24
	Mn-O_β	2.5	2.25(5)	2.25	0.002(2)			
	Mn-O_1	1	1.87(2)	1.94	0.008(1)			
	Mn-O_2	3	1.94(2)	2.02	0.008(1)			
	Mn-O_3	1	2.16(2)	2.20	0.003(2)			
Mn/HE-MHOF	Mn-O_4	1	2.21(2)	2.25	0.003(2)	0.7	2(1.92)	0.035
	Mn-C_1	2	2.86(2)	3.00	0.007(1)			
	Mn-O,$C_{(\Delta)}$	4	3.02(2)	3.15	0.007(1)			
	Mn-M	3	3.30(2)	3.37	0.006(1)			

Table S4

Measured reference material and HE-MHOF samples were fitted with CoO (*cubic*) from Materials Project[32] and simulated HE-MHOF structure (Figure S34), respectively. Results from Co K-edge EXAFS fitted parameters, including scattering path, coordination number (N), interatomic distance (R), inter atomic distance from the model (R_{eff}), Debey Waller factor (σ^2), amplitude reduction factor (S_O^2), energy shift parameter (ΔE), and R-factor, are given below. Co K-edge first-shell EXAFS of HE-MHOF was fitted with the fixed amplitude reduction factor (S_O^2), where S_O^2 was obtained from the reference material CoO. Finally, the total Co-K edge EXAFS fit of HE-MHOF was also performed with fixed S_O^2 and N from the first shell fit. Parentheses indicate a standard deviation of the last digit of fitted parameters, and the fixed parameters in the fitting are underlined. The Hanning window type, with dk = 1 is chosen to perform the Fourier transformation over the selected k-space range between 2 to 12 Å^{-1} for CoO, and 2 to 10 Å^{-1} for HE-MHOF, respectively. The fit to the model structure was performed between 1 and 4 Å for CoO. The fit to the HE-MHOF model structure was performed between 1 and 2.5 Å for the first coordination sphere, and between 1 and 4 Å for the additional coordination spheres. Δ in scattering path denotes obtuse triangle type. (Fitted plots in Figure S19)

Sample	***Scattering Path***	***N***	***R(Å)***	***R_{eff}(Å)***	***σ^2 (Å^2)***	***S_o^2***	***ΔE (eV)***	***R-factor***
CoO	Co-O	<u>1</u>	1.93(1)	1.90	0.007	1.0(1)	-1.3(1)	0.012
	Co-O	<u>3</u>	1.96(1)	1.96	0.007			
	Co-Co	<u>6</u>	3.05(3)	3.16	0.044			
	Co-Co	<u>6</u>	3.09(3)	3.20	0.044			
Co/HE-MHOF (1st Shell)	Co-O_α	4(1)	2.01(2)	1.98	0.011(2)	<u>1</u>	5.7(1.3)	0.022
	Co-O_β	2(1)	2.26(2)	2.14	0.011(2)			
Co/HE-MHOF	Co-O_α	<u>4</u>	2.01(1)	1.98	0.011(1)	<u>1</u>	3.84(1.3)	0.021
	Co-O_β	<u>2</u>	2.18(1)	2.14	0.011(1)			
	Co-C_1	<u>2</u>	3.10(8)	2.98	0.040(2)			
	Co-C_2	<u>2</u>	3.21(8)	3.10	0.040(2)			
	Co-C_3	<u>2</u>	2.92(8)	3.04	0.040(2)			
	Co-C_4	<u>2</u>	3.73(4)	3.89	0.040(2)			
	Co-O,$C_{(\Delta)\,1}$	<u>4</u>	3.24(8)	3.26	0.040(2)			
	Co-M_1	<u>2</u>	3.27(3)	3.28	0.005(2)			
	Co-M_2	<u>2</u>	3.27(3)	3.28	0.005(2)			
	Co-M_3	<u>2</u>	3.50(3)	3.51	0.005(2)			
	Co-O_1	<u>2</u>	3.51(3)	3.49	0.011(1)			
	Co-O_2	<u>2</u>	3.58(3)	3.56	0.011(1)			
	Co-O_3	<u>2</u>	3.64(3)	3.80	0.011(1)			
	Co-O_4	<u>4</u>	3.74(4)	3.90	0.011(1)			

Table S5

Measured reference material and HE-MHOF samples were fitted with NiO (*cubic*) from Materials Project[32] and simulated HE-MHOF structure (Figure S34), respectively. Results from Ni K-edge EXAFS fitted parameters, including scattering path, coordination number (N), interatomic distance (R), inter atomic distance from the model (R_{eff}), Debey Waller factor (σ^2), amplitude reduction factor ($S_O{}^2$), energy shift parameter (ΔE), and R-factor, are given below. Ni K-edge first-shell EXAFS of HE-MHOF was fitted with the fixed amplitude reduction factor ($S_O{}^2$), where $S_O{}^2$ was obtained from the reference material NiO. Finally, the total Ni-K edge EXAFS fit of HE-MHOF was also performed with fixed $S_O{}^2$ and N from the first shell fit. Parentheses indicate a standard deviation of the last digit of fitted parameters, and the fixed parameters in the fitting are underlined. The Hanning window type, with dk = 1 is chosen to perform the Fourier transformation over the selected k-space range between 3 to 13 $Å^{-1}$ for NiO, and 2 to 12 $Å^{-1}$ for HE-MHOF, respectively. The fit to the model structure was performed between 1.5 and 3.4 Å for NiO. The fit to the HE-MHOF model structure was performed between 1.3 and 2.5 Å for the first coordination sphere, and between 1.3 and 4 Å for the additional coordination spheres. Δ in scattering path denotes obtuse triangle type. (Fitted plots in Figure S21)

Sample	*Path*	*N*	*R(Å)*	*R_{eff}(Å)*	*σ^2 ($Å^2$)*	*$S_o{}^2$*	*ΔE (eV)*	*R-factor*
NiO	Ni-O	<u>6</u>	2.077(9)	2.09	0.006(1)	0.85(5)	-2.2(8)	0.012
	Ni-Ni	<u>12</u>	2.955(5)	2.96	0.006(1)			
Ni/HE-MHOF (1st Shell)	Ni-O_α	4(1)	2.03(2)	2.04	0.007(2)	<u>0.85</u>	2.5(1.2)	0.010
	Ni-O_β	2(1)	2.12(2)	2.12	0.007(2)			
Ni/HE-MHOF	Ni-O_α	<u>4</u>	2.04(1)	2.04	0.003(1)	<u>0.85</u>	2.7(0.6)	0.021
	Ni-O_β	<u>2</u>	2.13(1)	2.12	0.003(1)			
	Ni-C_1	<u>2</u>	2.96(6)	2.98	0.020(1)			
	Ni-C_2	<u>2</u>	3.03(6)	3.09	0.020(1)			
	Ni-C_3	<u>2</u>	3.14(6)	2.92	0.020(1)			
	Ni-C_4	<u>2</u>	3.98(2)	3.89	0.020(1)			
	Ni-O,$C_{(\Delta)\,1}$	<u>4</u>	3.25(6)	3.24	0.020(1)			
	Ni-O,$C_{(\Delta)\,2}$	<u>4</u>	3.28(6)	3.27	0.020(1)			
	Ni-M_1	<u>2</u>	3.28(6)	3.28	0.011(2)			
	Ni-M_2	<u>2</u>	3.53(3)	3.53	0.011(2)			
	Ni-M_3	<u>2</u>	3.56(3)	3.56	0.011(2)			
	Ni-O_1	<u>2</u>	3.51(3)	3.51	0.003(1)			
	Ni-O_2	<u>2</u>	3.58(3)	3.58	0.003(1)			
	Ni-O_3	<u>4</u>	3.80(3)	3.80	0.003(1)			
	Ni-O_4	<u>2</u>	3.92(3)	9.92	0.003(1)			

Table S6

Measured reference material and HE-MHOF samples were fitted with CuO (*monoclinic*) from Materials Project[32] and simulated HE-MHOF structure (Figure S34), respectively. Results from Cu K-edge EXAFS fitted parameters, including scattering path, coordination number (N), interatomic distance (R), inter atomic distance from the model (R_{eff}), Debey Waller factor (σ^2), amplitude reduction factor (S_O^2), energy shift parameter (ΔE), and R-factor, are given below. Cu K-edge first-shell EXAFS of HE-MHOF was fitted with the fixed amplitude reduction factor (S_O^2), where S_O^2 was obtained from the reference material CuO. Finally, the total Cu-K edge EXAFS fit of HE-MHOF was also performed with fixed S_O^2 and N from the first shell fit. Parentheses indicate a standard deviation of the last digit of fitted parameters, and the fixed parameters in the fitting are underlined. The Hanning window type, with dk = 1 is chosen to perform the Fourier transformation over the selected k-space range between 1.5 to 12 Å^{-1} for CuO, and 1.5 to 11 Å^{-1} for HE-MHOF, respectively. The fit to the model structure was performed between 1 and 3.5 Å for CuO. The fit to the HE-MHOF model structure was performed between 1 and 2.2 Å for the first coordination sphere, and between 1 and 4 Å for the additional coordination spheres. (Δ in scattering path denotes obtuse triangle type.) (Fitted plots in Figure S23)

Sample	*Path*	*N*	*R(Å)*	*R_{eff}(Å)*	*σ^2 (Å^2)*	*S_o^2*	*ΔE (eV)*	*R-factor*
CuO	Cu-O	4	1.95(1)	1.95	0.003(1)	0.65(4)	0.051(0.5)	0.008
	Cu-Cu	4	2.91(1)	2.93	0.011(2)			
	Cu-Cu	6	3.16(4)	3.27	0.022(7)			
	Cu-O	4	3.53(2)	3.52	0.003(1)			
	Cu-O	2	3.41(2)	3.40	0.003(1)			
	Cu-Cu	2	3.42(2)	3.41	0.003(1)			
Cu/HE-MHOF (1st Shell)	Cu-O_{α}	4.8 (0.6)	1.96(3)	1.94	0.006(2)	0.65	1.09(1.4)	0.014
Cu/HE-MHOF	Cu-O_{α}	4.8	1.96(1)	1.94	0.006(1)	0.65	2.18(1)	0.016
	Cu-C_1	2	2.99(2)	2.92	0.003(3)			
	Cu-C_2	2	3.02(2)	2.95	0.003(3)			
	Cu-C_3	2	3.15(2)	3.08	0.003(3)			
	Cu-C_4	2	3.79(4)	3.89	0.003(3)			
	Cu-O,$C_{(\Delta)1}$	4	3.24(2)	3.24	0.004(2)			
	Cu-O,$C_{(\Delta)2}$	4	3.27(2)	3.27	0.004(2)			
	Cu-M_1	2	3.32(2)	3.31	0.005(2)			
	Cu-M_2	2	3.47(2)	3.50	0.005(2)			
	Cu-M_3	2	3.69(2)	3.58	0.005(2)			
	Cu-O_1	2	3.48(2)	3.51	0.006(1)			
	Cu-O_2	2	3.55(2)	3.58	0.006(1)			
	Cu-O_3	2	3.69(4)	3.80	0.006(1)			
	Cu-O_4	2	3.82(4)	3.92	0.006(1)			

Table S7

Measured reference material and HE-MHOF samples were fitted with ZnO (*cubic*) from Materials Project[32] and simulated HE-MHOF structure (Figure S34), respectively. Results from Zn K-edge EXAFS fitted parameters, including scattering path, coordination number (N), interatomic distance (R), inter atomic distance from the model (R_{eff}), Debey Waller factor (σ^2), amplitude reduction factor ($S_O{}^2$), energy shift parameter (ΔE), and R-factor, are given below. Zn K-edge first-shell EXAFS of HE-MHOF was fitted with the fixed amplitude reduction factor ($S_O{}^2$), where $S_O{}^2$ was obtained from the reference material ZnO. Finally, the total Zn-K edge EXAFS fit of HE-MHOF was also performed with fixed $S_O{}^2$ and N from the first shell fit. Parentheses indicate a standard deviation of the last digit of fitted parameters, and the fixed parameters in the fitting are underlined. The Hanning window type, with dk = 1 is chosen to perform the Fourier transformation over the selected k-space range between 2 to 12 Å^{-1} for ZnO, and 2 to 10 Å^{-1} for HE-MHOF, respectively. The fit to the model structure was performed between 1 and 4 Å for ZnO. The fit to the HE-MHOF model structure was performed between 1 and 2.4 Å for the first coordination sphere, and between 1 and 4 Å for the additional coordination spheres. (Fitted plots in Figure S25)

Sample	*Path*	*N*	*R(Å)*	*R_{eff}(Å)*	*σ^2 (Å^2)*	*$S_o{}^2$*	*ΔE (eV)*	*R-factor*
ZnO	Zn-O	4	1.96(1)	1.97	0.004(1)	0.81(6)	3.7(8)	0.011
	Zn-Zn	12	3.227(8)	3.22	0.009(1)			
	Zn-O	12	3.74(3)	3.77	0.009(3)			
Zn/HE-MHOF (1st Shell)	Zn-O$_\alpha$	4.6(0.3)	1.99(1)	1.98	0.006(1)	0.81	5.5(0.5)	0.0014
	Zn-O$_\beta$	1.8(0.3)	2.21(1)	2.19	0.006(1)			
Zn/HE-MHOF	Zn-O$_\alpha$	4	2.00(1)	1.98	0.004(1)	0.81	6.8(0.8)	0.012
	Zn-O$_\beta$	2	2.22(1)	2.19	0.004(1)			
	Zn-C$_1$	2	3.25(8)	2.97	0.045(2)			
	Zn-C$_2$	2	3.29(8)	3.00	0.045(2)			
	Zn-C$_3$	2	3.38(8)	3.09	0.045(1)			
	Zn-C$_4$	2	3.97(2)	3.89	0.045(1)			
	Zn-M$_1$	2	3.4(8)	3.28	0.012(4)			
	Zn-M$_2$	2	3.48(2)	3.50	0.012(4)			
	Zn-M$_3$	2	3.6(2)	3.49	0.014(4)			
	Zn-O$_1$	2	3.41(2)	3.52	0.004(1)			
	Zn-O$_2$	2	3.42(2)	3.51	0.004(1)			
	Zn-O$_3$	4	3.89(3)	3.80	0.004(1)			
	Zn-O$_4$	2	4.01(3)	3.92	0.004(1)			

Table S8

The table showcases the dynamic evolution of X-ray Absorption Near-Edge Structure (XANES) parameters during an electrochemical process. The data is presented for various applied potentials, revealing changes in pre-edge peak height, edge position, and white line intensity. This *in-situ* XANES analysis of the five different metals (Mn, Co, Ni, Cu, Zn) of HE-MHOF offers valuable real-time information about the electrocatalyst during Oxygen Evolution Reaction, contributing to a deeper understanding of its electronic and structural dynamics.

Sample	Potential (V_{RHE})	XANES Energy Range (*e*V)	Pre-edge Peak Position (*e*V)	Pre-edge Peak height (a.u.)	Edge Position (E_o) (*e*V)	White Line Intensity (a.u.)	Normalized XANES Spectrum
Mn/HE-MHOF	Unbiased	6400-6600	6540.2	0.05	6552.1	1.67	Figure: 5a
Mn/HE-MHOF	1.7V	6400-6600	6541.5	0.08	6553.6	1.67	Figure: 5a
Mn/HE-MHOF	0.8V	6400-6600	6541.5	0.09	6553.5	1.54	Figure: 5a
Co/HE-MHOF	Unbiased	7600-7800	7708.7	0.05	7718	1.6	Figure: 5a
Co/HE-MHOF	1.7V	7600-7800	7709.3.3	0.06	7720.8	1.6	Figure: 5a
Co/HE-MHOF	0.8V	7600-7800	7708.7	0.05	7720.8	1.48	Figure: 5a
Ni/HE-MHOF	Unbiased	8200-8400	8334.5	0.11	8346.3	1.8	Figure: 5a
Ni/HE-MHOF	1.7V	8200-8400	8335.3	0.19	8348.3	1.7	Figure: 5a
Ni/HE-MHOF	0.8V	8200-8400	8334.5	0.20	8346.3	2.00	Figure: 5a
Cu/HE-MHOF	Unbiased	8850-9050	--	--	8987	1.56	Figure: 5a
Cu/HE-MHOF	1.7V	8850-9050	--	--	8987	1.44	Figure: 5a
Cu/HE-MHOF	0.8V	8850-9050	--	--	8987	1.56	Figure: 5a
Zn/HE-MHOF	Unbiased	9550-9750	--	--	9663.5	1.51	Figure: 5a
Zn/HE-MHOF	1.7V	9550-9750	--	--	9663.5	1.51	Figure: 5a
Zn/HE-MHOF	0.8V	9550-9750	--	--	9663.5	1.57	Figure: 5a

Table S9

ICP-MS data of HE-MHOF by degrading in concentrated HNO_3. Data is given with relative measurement uncertainty of U_{rel} (k=2) resulting in the relative errors of 15 % for each constituent metals Mn, Co, Ni, Cu, Zn. LOD – limit of detection, LOQ – limit of quantification.

Analyte	Mn(%)	ΔMn %	Co(%)	ΔCo %	Ni(%)	ΔNi %	Cu(%)	ΔCu %	Zn(%)	ΔZn %
HE-MHOF	17	3	22	3	22	3	22	3	17	3
Analyte	Mn(mg g^{-1})	ΔMn (mg g^{-1})	Co (mg g^{-1})	ΔCo(mg g^{-1})	Ni (mg g^{-1})	ΔNi(mg g^{-1})	Cu (mg g^{-1})	ΔCu (mg g^{-1})	Zn (mg g^{-1})	ΔZn (mg g^{-1})
HE-MHOF	62	9	78	12	80	12	80	12	63	9

	Mn(mg g^{-1})	Co(mg g^{-1})	Ni(mg g^{-1})	Cu(mg g^{-1})	Zn(mg g^{-1})
LOD	0.012	0.002	0.006	0.004	0.24
LOQ	0.047	0.007	0.033	0.019	0.85

Table S10

Unit cell parameters of the HE-MHOF, Co-MOF and Ni-MOF. The parameters were determined by three-dimensional electron diffraction (3D ED) and averaged over all measured grains. The biggest differences in axis length and angle size are the *a*-axis and the *β*-angle between HE-MHOF and monometallic (Ni/Co)-MHOF.

**Cell Parameters**	**HE-MHOF**	**Co-MHOF**	**Ni-MHOF**
a **(Å)**	19.95(6)	19.85(8)	19.747(9)
b **(Å)**	3.29(2)	3.271(5)	3.27(3)
c **(Å)**	6.23(2)	6.26(2)	6.25(3)
α **(º)**	90.7(7)	89.9(1)	90.0(9)
β **(º)**	97.0(2)	96.1(2)	96.3(1)
γ **(º)**	89.3(7)	89.7(2)	89.1(3)
**V($Å^3$)**	406(3)	403(1)	400(3)

Note S1. The presence of smaller particles in the HR-TEM images and their effect on OER.

The first and second HR-TEM images reveal nano-sized particles deposited on larger HE-MHOF particles, likely formed during the sonication process, and deposited during drop-casting on the TEM grid (**Figure 2c, S6**). However, the third and fourth images do not show these smaller particles. Though there remains a possibility of smaller particles to exist, even so, the ratio of such small particles to the actual crystallite is insignificant. Similarly, the absence of additional peaks in the PXRD pattern indicative of metal oxide or hydroxide suggests they are HE-MHOF particles. The possible existence of smaller HE-MHOF particles can't be discarded, which may have more active sites or better onset for electrocatalysis. Nevertheless, that only sums up a small contribution to the macroscopic contribution of the HE-MHOF structure.

Note S2. Selection of metals for high entropy matrix

<u>Initial choice of metals:</u> Manganese, which is bigger than Ni and Co—recognized for their OER capabilities—may be suitable for OER despite its lesser electrochemical stability in the +2 oxidation state than other *$M(OH)_2$. However, Mn can introduce structural distortions within the parent MHOF lattice. To mitigate this, we incorporate Cu and Zn, which are electrochemically inactive for OER and smaller but remain stable in the +2 oxidation state. These metals help stabilize the MHOF matrix and address potential defects that metals like Mn and Co introduce.

<u>Initial electrochemical assessments:</u> Our initial electrochemical measurements (**Figure S39a**) for 20% metal-doping in Ni-MHOF show improvements for Ni-Mn, Ni-Zn and Ni-Co MOFs for OER catalysis (better onset and current density) compared to Ni-MHOF. This signifies how doping these metals can improve the electrocatalytic performance of Ni-MHOF by simply altering the electronic picture of the Ni compound. However, Ni-Cu suppresses electrocatalysis performance. The choice of Zn in place of Cu becomes preferential to stabilize the +2 Ni matrix.

<u>Initial stability analysis:</u> Our TGA analyses observations (**Figure S39b**) indicate significant differences from our electrochemical measurements. While doping with other metals enhances the electrochemical performance without altering stability, the Ni-Cu structure exhibits a notable improvement in stability. Specifically, the Ni-Cu structure demonstrates enhanced thermal stability across an extended temperature range compared to other Ni-M structures. This improved stability likely stems from the more robust bonds copper forms with oxygen in the structure, which are crucial for the high entropy (HE) matrix.

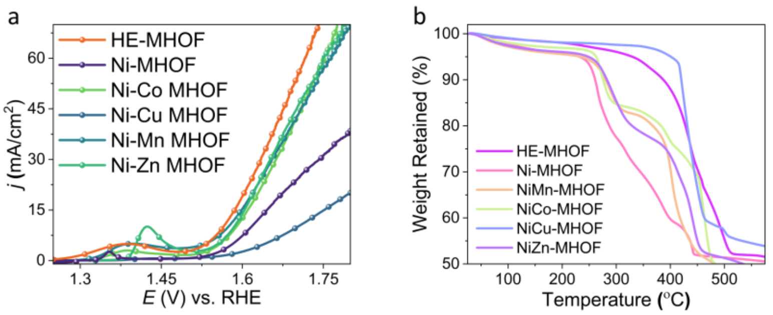

Figure S39. Comparison of OER performance among Ni-MHOF, Bimetallic (M doped ~20%)-MHOFs, HE-MHOF in 0.1 mol L^{-1} NaOH solution under the rotation speed of 1600 rpm on glassy carbon disk electrode. (b) Thermogravimetric analysis (TGA) of the same variants of MHOFs.

Note S3. Assessment of Possible X-ray beam induced effects

X-ray beam damage presents a significant risk during X-ray Absorption Spectroscopy (XAS), particularly in electrolyte-containing systems, where beam-induced radiolysis and sample degradation can influence experimental results. In solutions (i.e. electrolyte media) this becomes even more probable due to the higher cross-section upon X-ray interaction. Local heat induces air bubbles, which are known as radiolysis, that are known to happen in milliseconds.33 Recognizing the sensitivity of XAS to these effects, we have implemented several strategies to mitigate potential beam-induced damage in both our in-situ and ex-situ experiments. Explanation follows:

<u>Experimental Strategies to Minimize Beam-Induced Damage:</u>

To address the risks associated with prolonged X-ray exposure, particularly in electrolyte media, we designed an experimental setup that includes a closed-loop electrolyte flow system, and a bottom-to-top direction. This system prevents the formation of air bubbles, which were observed in earlier setups utilizing static electrolyte systems. The dynamic electrolyte flow also reduces the likelihood of localized radiolysis. A high-resolution camera monitors the cell during exposure, and so far, we have

not observed any significant colour changes on the Kapton film or sample degradation. These visual indicators, along with the consistence of the XANES spectra collected during our experiments, suggest that the system remains intact during irradiation. Additionally, the electrode preparation and cell modifications maintain the integrity of catalyst layers under varying potentials, which are particularly prone to accelerated electrochemical reactions and increased susceptibility to X-ray beam interactions.

Monitoring and Risk Assessment:

Beam-induced damage can manifest itself in several ways, including chemical changes (e.g., oxidation or reduction), structural modifications (e.g., bond breaking or new phase formation). To mitigate these risks, we used a highly monochromatic X-ray beam for all measurements and limited the acquisition time in ex-situ / in-situ experiments to minimal exposure while still acquiring high-quality data. Continuous monitoring of XANES spectra during in-situ experiments allows us to detect any anomalies indicative of beam damage. For example, we routinely check for abnormalities such as shallow absorption edges or undefined peak structures, both of which are classic signs of radiation damage. The absence of these effects in our data suggests that the precautions taken, including real-time monitoring of X-ray flux and careful calibration against a reference foil, have been effective in minimizing beam-induced alterations.

Data Integrity and Spectral Stability

In previous studies, beam damage has often been detected by broadening of the absorption edge, with shifts greater than 10 eV being a common indicator of sample degradation[34]. In contrast, our results show no such broadening or irregular features, both in ex-situ and in-situ measurements. The XANES spectra remained stable across multiple scans, further confirming that our cell design and monitoring strategies successfully minimize beam damage effects. Additionally, in response to concerns regarding beamtime limitations and their potential to mask damage over extended exposures, we have incorporated a critical comparison of XANES spectra in Figure S40, restricted to the same energy range as Figure 5. This comparison enhances the clarity of our data analysis and helps identify any potential beam-induced effects by visual comparison.

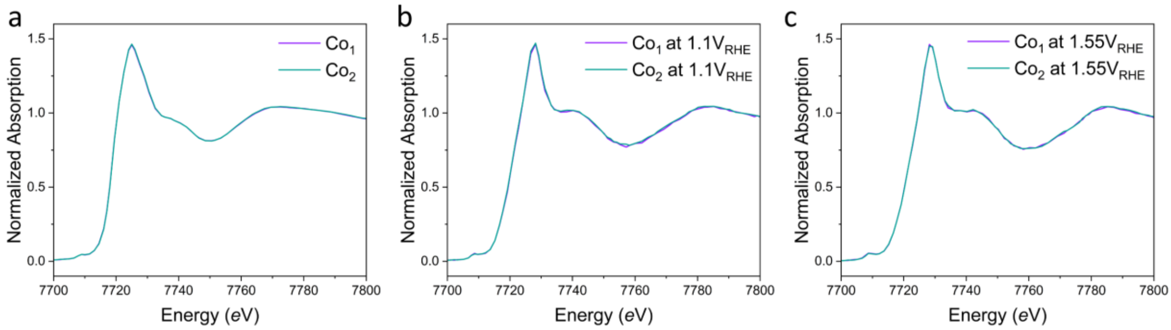

Figure S40. Comparison of Co K-edge XAS spectra of HE-MHOF collected in consecutive repetitions, indicating no evidence of beam damage. (a) Ex-situ Co XANES of HE-MHOF, (b) In-situ/operando Co XANES at 1.1 V_RHE, (c) In-situ/operando Co XANES at 1.55 V_RHE.

Through a combination of a closed-loop electrolyte system, continuous real-time monitoring, careful X-ray flux calibration, and reduced acquisition times, we have successfully minimized the risk of beam-induced damage. The stability of our XANES spectra, along with the absence of visible artifacts commonly associated with radiation damage, indicates that our experimental design effectively protects the integrity of our samples during X-ray irradiation. Nevertheless, we remain vigilant in assessing these effects and will continue to refine our protocols to mitigate any potential beam-induced phenomena.

Note S4. Clarification on samples of HE-MHOF and electrocatalytic performance

We would like to clarify that the high-entropy metal-organic framework (HE-MHOF) was synthesised multiple batches for all experiments presented in the manuscript. Furthermore, we have recorded electrocatalysis performance of three different batches and average activity with standard deviation have been shown in LSV trace of Figure 4a. This ensures that our results are not dependent on a single synthesis event but are representative of our synthesis process.

Note S5. Determination of formal oxidation states by operando XANES spectra

Initially, we used XANES spectra of the five different metals in HE-MHOF to estimate oxidation states by comparison with their respective divalent oxides. However, determining precise oxidation states in complex materials like HE-MHOF is challenging due to the sensitivity of the K-edge to both oxidation states and the bonding environment with neighbouring elements. Recognizing this limitation, we supplemented our XANES analysis with XPS data to further validate the oxidation states. For determining the edge position in the XANES spectra, we initially used the conventional approach of identifying the inflection point via its derivative. However, this method can be insufficient for capturing subtle energy shifts. Therefore, we employed the integral method (Valance state determination in method section), which provides a more precise determination of edge positions, especially when analysing narrow potential ranges (Figure 5d).

We acknowledge that two different classes of procedures are available: (1) Linear Combination Fitting (LCF) of XANES spectra with reference spectra is a possible method for quantitatively assigning formal oxidation states, but it presents significant challenges in the context of our high-entropy material, HE-MHOF; (2) purely mathematical tools such as Principal Component Analysis (PCA) or even a better variant so-called Non-negative Matrix Factorization (NMF) to extract the number of components that compose the final XANES spectrum. Although NMF is a promising mathematical tool, in this case it can still be challenging in the context for decomposing complex XANES spectra into meaningful components.

Calibration Issues: Accurate determination of oxidation states typically depends on comparison with representative reference compounds. However, finding suitable reference materials with well-defined oxidation states that closely resemble the complex environment of HE-MHOF is challenging. This issue is exacerbated by matrix effects, where the high-entropy nature of the material can significantly influence the edge position, complicating calibration.

Chemical Environment: The absorption edge is sensitive not only to oxidation state but also to the chemical environment, including factors such as coordination number, bond length, and ligand identity. These variations can shift the edge position, making it difficult to compare HE-MHOF directly with standard reference spectra during operando experiments.

Multiple Oxidation States: HE-MHOF exhibits multiple oxidation states during the Oxygen Evolution Reaction (OER), making it challenging to isolate and accurately quantify the contribution of each oxidation state to the XANES spectrum at specific applied potentials. Additionally, while LCF is a useful tool, it may overfit the data based on the input spectra provided, potentially leading to inaccurate interpretations without a detailed understanding of which oxidation states are stable under certain conditions.

Given these complexities, we recognize that determining precise formal oxidation states using LCF or NMF may not yield fully accurate results for HE-MHOF.

Note S6. Comparison of Ex-situ and operando XAS data

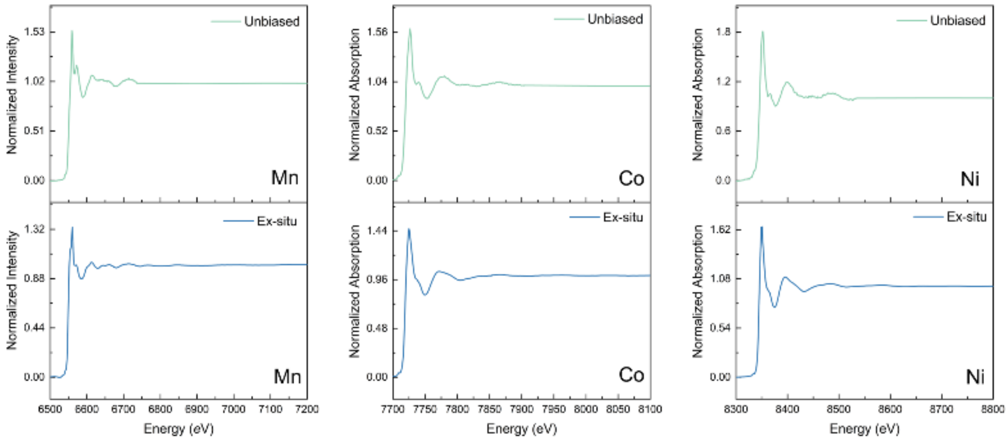

Figure S41. Comparison of ex-situ and operando XAS spectra of Mn, Co, Ni collected in fluorescence mode (unbiased) and transmission mode (ex-situ).

A direct comparison between the ex-situ and operando XAS spectra is challenging due to the significant differences. This discrepancy is primarily attributed to the formation of a double layer and the adsorption of intermediates on the catalytic sites during operando conditions, as previously reported in the literature[35]. These factors can significantly influence the local electronic environment of the metal centres, leading to shifts in the absorption edges and changes in spectral features.

Note S7. Self-critical analysis of present in-situ/operando XAS data

We acknowledge the concern about the limited availability of in-situ/operando XAS measurements due to the long-term planning required for beamtime allocation. While further experiments are challenging at this stage, we emphasize that our primary focus was the synthesis and characterization of a novel multi-metallic (e.g. five metals) high-entropy hydroxide-based single-crystalline MOF structure. In general, the structural and electronic analyses of HE-MHOF provide a comprehensive understanding this new class of materials.

Electrocatalytic Activity and Data Reproducibility:

We have carried out extensive experiments to confirm the reproducibility of the electrocatalytic activity of our material. The data from repeated experiments strengthen our conclusions about its promising application in the electrocatalytic oxygen evolution reaction (OER).

OER Mechanistic Insights:

To gain a deeper understanding of the OER mechanism on the high-entropy surface of our material, we performed operando XAS during OER electrocatalysis. These measurements provided information about the oxidation states of the catalytic centres by correlating them with reference materials. Additionally, we constructed Pourbaix diagrams and potential energy surface diagrams using ab-initio calculations to identify possible active species and their stabilization on the surface. These combined results improve our understanding of the OER reaction-mechanism at the onset potential.

Impact of non-reversibility or non-repeatability effect on conclusion:

We agree that the higher number of oxidation-reduction cycles (remains same up to the 40th cycle in figure 4c) or higher applied potential range could influence the stabilisation of other active oxide/oxyhydroxide or other species. This can be a reason for changes in the Tafel slope at higher overpotentials. However, we believe that this does not invalidate our results obtained during the first cycle up to the onset potential (1.55 V_{RHE}). Our proposed mechanism serves as an initial step toward a comprehensive understanding of the OER reaction-mechanism at the on-set potential on the high-entropy surfaces. An extensive study on mechanistic understanding to find the bigger picture on high entropy surface is still an open challenge. We have revised the manuscript to explicitly state that the XAS data presented is from one particular cycle, acknowledging the uncertainty in oxidation state determination is calculated from the fitting and measurement uncertainty at the beamline, not by the repeatability of the measurements.

Self-Critical Analysis and Acknowledgment of Limitations:

We recognize that determining oxidation states by comparing K-edge energies of XANES with pure metal oxides/hydroxides may have limitations. The electronic environment of a catalytic site can be significantly modified by its surroundings in a high-entropy material, making such comparisons is tentative. Currently, we lack a more accurate method to determine precise oxidation state from XANES spectra of the catalytic centres in high-entropy surface.

References

1. Nam, K.-W., Kim, M. G. & Kim, K.-B. *In Situ* Mn K-edge X-ray Absorption Spectroscopy Studies of Electrodeposited Manganese Oxide Films for Electrochemical Capacitors. *J. Phys. Chem. C* **111**, 749–758 (2007).

2. Wang, D. *et al. In Situ* X-ray Absorption Near-Edge Structure Study of Advanced NiFe(OH) $_x$ Electrocatalyst on Carbon Paper for Water Oxidation. *J. Phys. Chem. C* **119**, 19573–19583 (2015).

3. Gorlin, Y. *et al.* In Situ X-ray Absorption Spectroscopy Investigation of a Bifunctional Manganese Oxide Catalyst with High Activity for Electrochemical Water Oxidation and Oxygen Reduction. *J. Am. Chem. Soc.* **135**, 8525–8534 (2013).

4. Moysiadou, A., Lee, S., Hsu, C.-S., Chen, H. M. & Hu, X. Mechanism of Oxygen Evolution Catalyzed by Cobalt Oxyhydroxide: Cobalt Superoxide Species as a Key Intermediate and Dioxygen Release as a Rate-Determining Step. *J. Am. Chem. Soc.* **142**, 11901–11914 (2020).

5. Schröder, J. *et al.* Tracking the Dynamics of a Ag-MnO $_x$ Oxygen Reduction Catalyst Using In Situ and Operando X-ray Absorption Near-Edge Spectroscopy. *ACS Energy Lett.* **8**, 2962–2969 (2023).

6. Zhao, S. *et al.* Ultrathin metal–organic framework nanosheets for electrocatalytic oxygen evolution. *Nat. Energy* **1**, 16184 (2016).

7. International X-ray Absorption Society.

8. Gehringer, D., Friák, M. & Holec, D. Models of configurationally-complex alloys made simple. *Comput. Phys. Commun.* **286**, 108664 (2023).

9. Perl, J., Shin, J., Schümann, J., Faddegon, B. & Paganetti, H. TOPAS: An innovative proton Monte Carlo platform for research and clinical applications. *Med. Phys.* **39**, 6818–6837 (2012).

10. Riesemeier, H. *et al.* Layout and first XRF applications of the BAM *line* at BESSY II: Layout and XRF applications of the BAM *line* at BESSY II. *X-Ray Spectrom.* **34**, 160–163 (2005).

11. Ravel, B. & Newville, M. *ATHENA* , *ARTEMIS* , *HEPHAESTUS* : data analysis for X-ray absorption spectroscopy using *IFEFFIT*. *J. Synchrotron Radiat.* **12**, 537–541 (2005).

12. Funke, H., Chukalina, M. & Scheinost, A. C. A new *FEFF* -based wavelet for EXAFS data analysis. *J. Synchrotron Radiat.* **14**, 426–432 (2007).

13. Xia, Z., Zhang, H., Shen, K., Qu, Y. & Jiang, Z. Wavelet analysis of extended X-ray absorption fine structure data: Theory, application. *Phys. B Condens. Matter* **542**, 12–19 (2018).

14. Ito, S. *et al.* Structure determination of small molecule compounds by an electron diffractometer for 3D ED/MicroED. *CrystEngComm* **23**, 8622–8630 (2021).

15. Truong, K.-N. *et al.* Making the Most of 3D Electron Diffraction: Best Practices to Handle a New Tool. *Symmetry* **15**, 1555 (2023).

16. Rigaku Oxford Diffraction CrysAlisPro Software System, Rigaku Corporation: Wroclaw, Poland, 2023.

17. Kresse, G. & Hafner, J. *Ab initio* molecular dynamics for liquid metals. *Phys. Rev. B* **47**, 558–561 (1993).

18. Kresse, G. & Hafner, J. *Ab initio* molecular-dynamics simulation of the liquid-metal–amorphous-semiconductor transition in germanium. *Phys. Rev. B* **49**, 14251–14269 (1994).

19. Perdew, J. P., Burke, K. & Ernzerhof, M. Generalized Gradient Approximation Made Simple. *Phys. Rev. Lett.* **77**, 3865–3868 (1996).

20. Perdew, J. P., Burke, K. & Ernzerhof, M. Generalized Gradient Approximation Made Simple [Phys. Rev. Lett. 77, 3865 (1996)]. *Phys. Rev. Lett.* **78**, 1396–1396 (1997).

21. Gehringer, D., Friák, M. & Holec, D. Models of configurationally-complex alloys made simple. *Comput. Phys. Commun.* **286**, 108664 (2023).

22. Bockris, J. O. & Kita, H. Analysis of Galvanostatic Transients and Application to the Iron Electrode Reaction. *J. Electrochem. Soc.* **108**, 676 (1961).

23. Bockris, J. O. & Reddy, A. K. N. *Modern Electrochemistry: An Introduction to an Interdisciplinary Area Volume 1*. (Springer US, Boston, MA, 1970). doi:10.1007/978-1-4615-8600-5.

24. Mefford, J. T., Zhao, Z., Bajdich, M. & Chueh, W. C. Interpreting Tafel behavior of consecutive electrochemical reactions through combined thermodynamic and steady state microkinetic approaches. *Energy Environ. Sci.* **13**, 622–634 (2020).

25. Shinagawa, T., Garcia-Esparza, A. T. & Takanabe, K. Insight on Tafel slopes from a microkinetic analysis of aqueous electrocatalysis for energy conversion. *Sci. Rep.* **5**, 13801 (2015).

26. Dau, H., Liebisch, P. & Haumann, M. X-ray absorption spectroscopy to analyze nuclear geometry and electronic structure of biological metal centers?potential and questions examined with special focus on the tetra-nuclear manganese complex of oxygenic photosynthesis. *Anal. Bioanal. Chem.* **376**, 562–583 (2003).

27. Nørskov, J. K., Studt, F., Abild-Pedersen, F. & Bligaard, T. *Fundamental Concepts in Heterogeneous Catalysis*. (Wiley, 2014). doi:10.1002/9781118892114.

28. Man, I. C. *et al.* Universality in Oxygen Evolution Electrocatalysis on Oxide Surfaces. *ChemCatChem* **3**, 1159–1165 (2011).

29. Rossmeisl, J., Qu, Z.-W., Zhu, H., Kroes, G.-J. & Nørskov, J. K. Electrolysis of water on oxide surfaces. *J. Electroanal. Chem.* **607**, 83–89 (2007).

30. *Handbook of Materials Modeling: Applications: Current and Emerging Materials*. (Springer International Publishing, Cham, 2020). doi:10.1007/978-3-319-44680-6.

31. Nørskov, J. K. *et al.* Origin of the Overpotential for Oxygen Reduction at a Fuel-Cell Cathode. *J. Phys. Chem. B* **108**, 17886–17892 (2004).

32. Jain, A. *et al.* Commentary: The Materials Project: A materials genome approach to accelerating materials innovation. *APL Mater.* **1**, 011002 (2013).

33. Loh, Z.-H. *et al.* Observation of the fastest chemical processes in the radiolysis of water. *Science* **367**, 179–182 (2020).

34. Zabilska, A. *et al.* Beware of beam damage under reaction conditions: X-ray induced photochemical reduction of supported VO_x catalysts during *in situ* XAS experiments. *Phys. Chem. Chem. Phys.* **24**, 21916–21926 (2022).

35. Gomes, B. F. *et al.* Following Adsorbed Intermediates on a Platinum Gas Diffusion Electrode in H_3PO_3-Containing Electrolytes Using In Situ X-ray Absorption Spectroscopy. *ACS Catal.* **12**, 11472–11484 (2022).
